# Antenatal magnesium sulphate for preterm foetal neuroprotection in low- and middle-income countries: a scoping review of research studies and guidelines

**DOI:** 10.7189/jogh.16.04088

**Published:** 2026-03-20

**Authors:** Shona Goldsmith, Tasneem Karim, Sarah McIntyre, Alice Rumbold, Atul Malhotra, Gulam Khandaker, Sugandha Arya, Emily Shepherd

**Affiliations:** 1 Cerebral Palsy Alliance Research Institute, Speciality of Child and Adolescent Health, Sydney Medical School, Faculty of Medicine and Health, The University of Sydney, Sydney, New South Wales, Australia; 2 Children’s Hospital Westmead Clinical School, Faculty of Medicine and Health, The University of Sydney, Sydney, New South Wales, Australia; 3 South Australian Health and Medical Research Institute (SAHMRI) Women and Kids,; SAHMRI, Adelaide, South Australia, Australia; 4 Adelaide Medical School, Adelaide University, Adelaide, South Australia, Australia; 5 Monash Newborn, Monash Children’s Hospital, Melbourne, Victoria, Australia; 6 Department of Paediatrics, Monash University, Melbourne, Victoria, Australia; 7 The Ritchie Centre, Hudson Institute of Medical Research, Melbourne, Victoria, Australia; 8 Central Queensland Hospital and Health Service, Rockhampton, Queensland, Australia; 9 Department of Paediatrics, Vardhman Mahavir Medical College and Safdarjung Hospital, Ansari Nagar, New Delhi, India

## Abstract

**Background:**

Antenatal magnesium sulphate reduces the risk of cerebral palsy (CP) for infants born very preterm. While endorsed by the World Health Organization for global implementation in 2015, studies underpinning this recommendation were conducted in high-income countries. Our objective was to systematically gather, organise, and map published research studies on the use of antenatal magnesium sulphate for preterm foetal neuroprotection in low- and middle-income countries (LMICs), and to obtain existing relevant national and international clinical practice guidelines from (or for) LMICs.

**Methods:**

Following scoping review methods, we searched nine databases and the websites of societies/ministries of health for relevant qualitative or quantitative studies and national or international guidelines, published from 2015, from any LMIC. We screened each publication for inclusion, and two reviewers independently extracted information. Content analysis included narrative summaries and descriptive statistics.

**Results:**

In total, 57 research studies (12 randomised controlled trials) and 25 clinical guidelines were included in the analysis. Most (n = 75) were in English, from lower-middle (n = 45) and upper-middle (n = 31) countries, and published between 2020 and 2025 (n = 60). The most common research scope was effects and/or safety (n = 38). The remaining studies focused on intervention uptake or quality improvement programmes (n = 10), mechanisms of action (n = 5), or regimen comparisons (n = 4). Short-term outcomes were common, and CP was described in only four studies. Regarding clinical guidelines, magnesium sulphate was usually included in general guidelines (n = 24), those published by professional associations (n = 18), or those published by government bodies (n = 6). After categorisation, an upper gestational limit of 32 weeks was most common (n = 18). Treatment regimens varied, commonly including a 4 g intravenous loading dose (n = 12) and a 1 g/h intravenous maintenance dose (n = 11). One in three recommended no specific regimen.

**Conclusions:**

A sizeable number of heterogeneous studies and clinical guidelines exist, primarily from middle-income countries, regarding magnesium sulphate for neuroprotection. Further context-specific research may include regimen comparisons, impact, and implementation studies, informing future updates to clinical guidelines globally.

**Registration:**

OSF 10.17605/OSF.IO/ASN67

Cerebral palsy (CP) is a life-long physical disability. This disorder of movement and posture leads to limitations in activity [1,2], and may be accompanied by disorders of sensation, cognition, communication, behaviour, epilepsy and pain [1,3]. Global estimates of CP indicate a recent decline in birth prevalence in high-income countries (HICs) to 1.6 per 1000 live births, amid improvements in maternal and neonatal care and public health [4]. Until recently, limited data were available from low- and middle-income countries (LMICs), but findings suggest a markedly higher birth prevalence [4], and rapidly expanding CP registers across LMICs are offering new epidemiological insights [5,6]. While CP results from a non-progressive maldevelopment or injury to the foetal or infant brain [1], many risk factors for CP have been identified, including infections, congenital anomalies, genetic conditions, hypoxic-ischaemic encephalopathy, and post-neonatal events [2,7]. Preterm birth is a seminal risk factor for CP, and the impact of preterm birth is highest in LMICs [8], where it is expected that children with increasingly lower gestational age will survive, compounding the burden.

Antenatal magnesium sulphate is now recognised as one of only two interventions for the prevention of CP supported by high-certainty evidence [9,10]. Its foetal neuroprotective potential was first documented in observational studies during the 1990s [11,12]. Subsequently, several randomised controlled trials (RCTs) assessed its impact on death and CP when administered to women prior to preterm birth [13–16]. In 2009, a Cochrane systematic review confirmed its neuroprotective benefit for the first time. It demonstrated that 63 preterm babies (95% confidence interval (CI) = 44–155) need to be exposed to antenatal magnesium sulphate to prevent one child developing CP (risk ratio (RR) = 0.68; 95% CI = 0.54–0.87) [17].

Following the compelling Cochrane review findings [17], the World Health Organization (WHO) 2015 guidelines on interventions to improve preterm birth outcomes strongly endorsed that ‘the use of magnesium sulphate is recommended for women at risk of imminent preterm birth before 32 weeks of gestation for prevention of CP in the infant and child’ [18]. Professional bodies in many HICs recommended this treatment in clinical practice guidelines [19], and broader international adoption ensued [20].

In response to evidence from new RCTs [21,22] and follow-up of original RCTs [23,24], a 2024 update to the Cochrane review was undertaken, which reaffirmed the neuroprotective benefits of antenatal magnesium sulphate [25]. The updated review included data from six RCTs, involving 5917 women and their 6759 children. However, it also highlighted an important limitation – all included RCTs were conducted in HICs. The authors recommended that the generalisability of findings to LMICs should be further considered [25]. A companion editorial called for ‘implementation into clinical practice to be accelerated globally to benefit preterm babies’ [20].

The potential benefits of improved uptake in LMICs are notable, given the markedly higher prevalence of both preterm birth and CP in these settings [4,8]. Magnesium sulphate also has intrinsic advantages as a preventive intervention, particularly for LMICs. These include relatively low cost and accessibility, a long history of antenatal use for prevention and treatment of eclampsia, and a well-established safety profile [26–28].

A decade after the WHO recommendation to implement antenatal magnesium sulphate for foetal neuroprotection [18], an overview of research and clinical guidelines from LMICs is timely and warranted. This can identify knowledge gaps regarding research and clinical guidelines about magnesium sulphate in LMICs and guide future context-specific research and clinical practice. Thus, the aims of this scoping review were to systematically gather, organise, and map published research studies on the use of antenatal magnesium sulphate for preterm foetal neuroprotection from LMICs, and to obtain existing national and international clinical practice guidelines on antenatal magnesium sulphate use for preterm foetal neuroprotection from (or for) LMICs.

## METHODS

In this scoping review, we followed a registered study protocol [29] – guided by the proposed framework of Arksey and O’Malley [30] and methodology from the Joanna Briggs Institute [31,32]. In reporting, we followed the PRISMA-ScR statement [33] (Table S1 in the **Online Supplementary Document**). We aimed to address the following questions: What is the volume and nature (including geographical distribution) of research studies assessing antenatal magnesium sulphate for preterm foetal neuroprotection from LMICs? What is the availability (including geographical distribution) and content of current national and international clinical practice guidelines on antenatal magnesium sulphate for preterm foetal neuroprotection from/for LMICs? These questions lend themselves to a scoping review, enabling a broad overview to identify available research studies and guidelines, their key characteristics, and potential knowledge gaps [34].

### Eligibility criteria

For research studies, qualitative and quantitative studies (such as RCTs, cohort studies, before-and-after studies, and descriptive studies), as well as mixed-methods studies, were eligible for inclusion. Reviews and case reports were excluded from the analysis. We included studies published in any language and used Google Translate for translation into English as required.

We followed the Population, Concept, Context framework. Eligible populations were health care service users and health care professionals. Studies of antenatal magnesium sulphate for preterm foetal neuroprotection were eligible. We excluded studies of magnesium sulphate for the prevention or treatment of eclampsia, or for tocolysis in preterm labour. Studies published from 1 November 2015 onwards were eligible, following publication of the relevant WHO recommendation [18]. This date was selected because endorsement by the WHO can catalyse the adoption of health interventions globally, and recommendations may inform the development and implementation of national guidelines. Studies from any setting in an LMIC were included (any country defined by the World Bank country classification by income level as being low, lower-middle, or upper-middle income at the time of conducting the final search (13 January 2025). We categorised countries by their World Bank income level classification on the same date.

For clinical practice guidelines, inclusion criteria were: the provision of recommendation(s) related to the use of antenatal magnesium sulphate for preterm foetal neuroprotection; developed by a recognised committee or a medical society for national or international use in a LMIC (defined as above) and available in any language (translated as above, as required); published from 1 November 2015 onwards (as above) and accessible in the public domain. If multiple versions of the same guideline were identified, we included only the most recent version. We excluded guidelines developed for local or hospital use. Given that our search strategy was focused on national societies, we deviated from our protocol and also excluded regional guidelines. Recognising great variation in the reporting of methodological detail and in order to be inclusive, we deviated from our protocol (which specified that we would only include guidelines that ‘meet the definition of clinical practice guidelines by the Institute of Medicine’ (*i.e.* informed by a systematic review of evidence) [29]. The vast majority of ‘guidelines’ identified did not report a systematic review of evidence. Acknowledging that excluding all such guidelines would vastly limit the utility of the review, we included all documents that self-identified as national or international clinical guidelines, protocols, recommendations or standards.

### Search strategy

We undertook a comprehensive search of CENTRAL, Embase, MEDLINE, Global Index Medicus, Trip Medical Database, ProQuest Dissertations & Theses Global, Google Scholar, ClinicalTrials.gov, and International Clinical Trials Registry Platform from 1 November 2015 to 13 January 2025. Where applicable, we used a combination of controlled vocabulary and free-text terms, guided by our selection criteria (Appendix S1 in the **Online Supplementary Document**). The reference lists of relevant studies (and excluded but relevant reviews) were also searched. For guidelines, we searched the websites of national societies of obstetrics and gynaecology (LMIC member societies of the International Federation of Gynaecology and Obstetrics) [35] and national ministries of health. An extensive web search was conducted to identify the websites of national ministries of health, using English keywords and translating them into the relevant languages for each country.

We imported citations into EndNote, version 20 (Clarivate, Philadelphia, Pennsylvania, USA) as separate files. Files were merged and duplicates removed, prior to importation into Covidence for further deduplication and study screening.

### Study and guideline selection, data extraction and charting

Two reviewers (ES and/or SG and/or TK) independently screened each title and abstract and the full text of potentially eligible studies and guidelines. Disagreements were settled through discussion. Standardised data extraction forms were created, piloted and refined. Data were extracted from each study and guideline by two reviewers (ES/or SG, and/or TK) independently, with discrepancies resolved by discussion. For research studies, information extracted included format, language, design, dates and setting, aim/purpose, magnesium sulphate evidence cited, population, magnesium sulphate and comparator characteristics and outcomes. We also extracted the authors’ key findings and conclusions. The main scope of the studies and the authors’ corresponding conclusions were pragmatically categorised to provide an overarching synthesis of the evidence base. We devised and refined categories during the data extraction process. The scope of each study was first categorised by the study aim: effects/safety, mechanism of action, regimen comparison, or uptake/quality improvement programme. The corresponding conclusions of the authors were then categorised within each scope: effects/safety (potential/no benefit, potential/no harm), mechanism of action (potential/no support), regimen comparison (comparable effects/specific regimen recommendation), and uptake/quality improvement programme (low (< 50%), moderate (50–80%), high (> 80%) reported uptake). For guidelines, the extracted information included author/developer, language, country, scope of the guideline (general or intervention-specific), magnesium sulphate evidence cited, and details of relevant recommendation(s). We did not contact study or guideline authors to obtain/confirm information. Aligned with scoping review guidance, a critical appraisal of the studies and guidelines was not conducted.

Quantitative and qualitative content analysis were descriptive in nature. Tables and figures are presented alongside narrative summaries of the studies and guidelines, with some use of descriptive statistics (percentages).

## RESULTS

**Figure 1:**
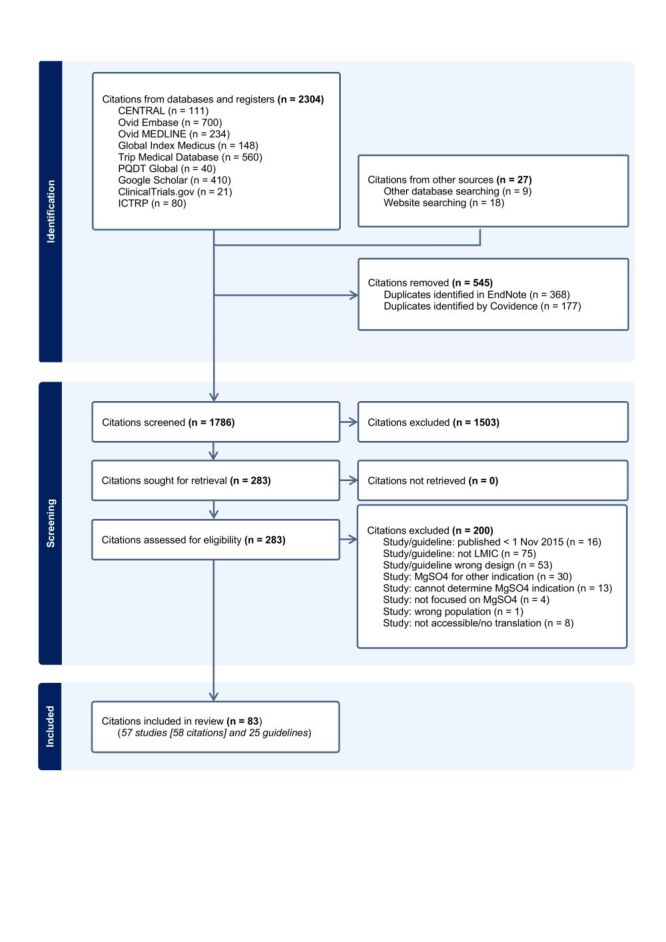
PRISMA-ScR flow diagram.

**Figure 2:**
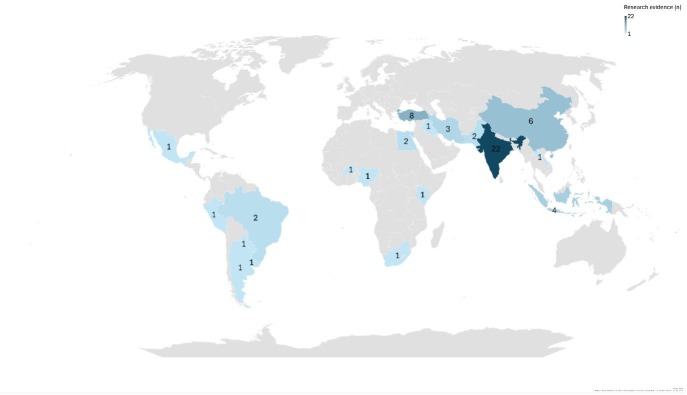
Geographical distribution of research studies. Powered by Bing. (C) Australian Bureau of Statistics, GeoNames, Microsoft, Navinfo, Open Places, OpenStreetMap, Overture Maps Foundation, Zenrin.

After screening and review, a total of 83 citations were included relating to 57 research studies [36–93] (one study, two citations) [65,71] and 25 guidelines [94–116] (**Figure 1**). The majority of research studies and guidelines were published in English [36–64,66–78,80,82–97,100–111,114–117], between 2020–2025 [38–41,43–46,48–50,52–56,58–62,64,67–69,71, 72,74–76,80,82–84,86–94,98,101–108,113–116,118] (**Table 1**, **Table 2**). While studies and guidelines were identified from all six WHO regions, all but one [61] originated from middle-income countries (**Figure 2**). There were considerable variations in recommended regimens and evidence cited.

**Table 1:** Characteristics of research studies of magnesium sulphate for foetal neuroprotection, n (%)

Characteristics	Total studies (n = 57)	Full papers only (n = 48)
**Publication year**		
2015–2019	15 (26.3)	10 (20.8)
2020–2025	42 (73.7)	38 (79.2)
World Bank country classification by income level*		
Low	1 (1.8)	1 (2.1)
Lower-middle	33 (57.9)	27 (56.3)
Upper-middle†	23 (40.4)	20 (41.7)
**Language**		
English	55 (96.5)	46 (95.8)
Other‡	2 (3.5)	2 (4.2)
**Publication format§**		
Trial registration	4 (7.0)	NA
Abstract	5 (8.8)	NA
Full-text	48 (84.2)	48 (100.0)
**Main scope of publication (in relation to magnesium sulphate)**		
Effects/safety	38 (66.7)	36 (75.0)
Mechanism of action	5 (8.8)	4 (8.3)
Regimen comparison	4 (7.0)	2 (4.2)
Uptake/quality improvement programme	10 (17.5)	6 (12.5)
**The authors’ overall conclusion regarding magnesium sulphate¶**		
Effects/safety		
*Potential benefits (no harms)*	27 (75.0)	27 (75.0)
*Potential benefits and harms*	1 (2.8)	1 (2.8)
*Potential harms (no benefits)*	2 (5.6)	2 (5.6)
*No clear benefits and/or harms*	5 (13.9)	5 (13.9)
*Unclear*	1 (2.8)	1 (2.8)
Mechanism of action		
*Potential support*	2 (50.0)	2 (50.0)
*Not supported*	2 (50.0)	2 (50.0)
Regimen comparison/evaluation		
*Comparable effects*	2 (66.7)	1 (50.0)
*Specific regimen recommendation*	1 (33.3)	1 (50.0)
Uptake/quality improvement		
*Low reported uptake*	4 (40.0)	2 (33.3)
*Moderately reported uptake*	3 (30.0)	3 (50.0)
*High reported uptake*	3 (30.0)	1 (16.7)
*Not applicable (trial registrations)*	4 (NA)	NA
**Design║**		
RCT	12 (21.1)	7 (14.6)
Prospective cohort	12 (21.1)	12 (25.0)
Retrospective cohort	10 (17.5)	10 (20.8)
Cross-sectional	5 (8.8)	4 (8.3)
Case series	7 (12.3)	6 (12.5)
Other	6 (10.5)	5 (10.4)
Unclear	5 (8.8)	4 (8.3)
Enrolled participants (n)**		
< 50	4 (7.0)	3 (6.3)
50–99	21 (36.8)	16 (37.5)
100–249	17 (29.8)	13 (31.3)
250–499	8 (14.0)	6 (12.5)
500–999	4 (7.0)	3 (6.3)
≥ 1000	3 (5.3)	3 (6.3)
**Gestational age in weeks (upper limit)**		
< 28	1 (1.8)	0 (0.0)
≤ 32	26 (45.6)	23 (47.9)
≤ 34	18 (31.6)	16 (33.3)
≤ 36	5 (8.8)	4 (8.3)
Not reported	7 (12.3)	5 (10.4)
**Magnesium sulphate regimen mode**		
Intravenous	43 (75.4)	38 (79.2)
Intravenous and intramuscular	1 (1.8)	1 (2.1)
Not reported	13 (22.8)	9 (18.8)
**Magnesium sulphate loading dose regimen in g**		
4††	35 (61.4)	32 (66.7)
4 or 6	1 (1.8)	1 (2.1)
6	5 (8.8)	4 (8.3)
Nil	1 (1.8)	0 (0.0)
Not reported	15 (26.3)	11 (22.9)
**Magnesium sulphate maintenance dose regimen in g/h‡‡**		
1	25 (43.9)	22 (47.9)
< 1	1 (1.8)	1 (2.1)
> 1	7 (12.3)	7 (14.6)
Varied dose reported	5 (8.8)	2 (4.2)
Not reported	15 (26.3)	11 (22.9)
Nil (loading dose only)	4 (7.0)	4 (8.3)
**Duration of maintenance dose**		
12 h/or until birth	10 (17.5)	8 (16.7)
24 h/or until birth	18 (31.6)	17 (35.4)
> 24 h	1 (1.8)	1 (2.1)
Other/varied duration reported	6 (10.5)	5 (10.4)
Not reported	18 (31.6)	13 (27.1)
Nil (loading dose only)	4 (7.0)	4 (8.3)
**Control/comparison**		
Placebo	6 (10.5)	5 (10.4)
No magnesium sulphate	32 (56.1)	26 (54.2)
Alternative magnesium sulphate regimen	2 (3.5)	1 (2.1)
Other comparison	6 (10.5)	5 (10.4)
Nil	11 (19.3)	11 (22.9)
**Outcomes reported**		
Maternal	31 (54.4)	24 (50.0)
Foetal/neonatal	44 (77.2)	38 (79.2)
Long-term infant/child	14 (24.6)	11 (22.9)
Cerebral palsy	4 (7.0)	3 (6.3)
Death	31 (54.4)	28 (58.3)
**Evidence cited**		
RCTs	35 (61.4)	35 (72.9)
2009 Cochrane systematic review	23 (40.4)	23 (47.9)
2015 WHO guideline	12 (21.1)	12 (25.0)
Other relevant systematic review(s)/meta-analysis(es) or guideline(s)	39 (68.4)	38 (79.2)

NA – not applicable, RCT – randomised controlled trial, WHO – World Health Organization

*Countries were categorised by their World Bank income level on 13 January 2025.

†Includes one research publication that included data from five countries: one lower-middle, three upper-middle and one high-income country.

‡Languages of publication: Indonesian and Spanish.

§Format: abstract includes conference abstract (n = 3) and abstract available only (n = 2).

¶Authors’ overall conclusion only; no quality appraisal completed.

║Design: likely study design categorised. Both self-reported and the likely study design are reported in Table S3 in the **Online Supplementary Document**.

**Studies reported by number of women enrolled (n = 34), infants enrolled (n = 22), or mother/infant units enrolled (n = 1). For full-text studies: women enrolled (n = 27), infants enrolled (n = 20), or mother/infant units enrolled (n = 1).

††Includes one study reporting a loading dose of 4.5 g.

‡‡Maintenance dose: 1 g/h includes one study reporting 4g/4 h.

**Table 2:** Characteristics of guidelines with magnesium sulphate for fetal neuroprotection recommendations, n (%)

Characteristics	Total guidelines
**Year of publication**	
2015–2019	7 (28.0)
2020–2024	18 (72.0)
World Bank classification*	
Low	0 (0.0)
Lower-middle	12 (48.0)
Upper-middle†	8 (32.0)
International	5 (20.0)
**Language**	
English	20 (80.0)
Translated by Google‡	5 (20.0)
**Author/developer**	
Obstetrics and gynaecology/perinatal professional association	18 (72.0)
Government	6 (24.0)
Other (WHO)	1 (4.0)
**Guideline type**	
Specific: magnesium sulphate for foetal neuroprotection	1 (4.0)
General: preterm labour/PROM/PPROM§	12 (52.0)
General: obstetric/newborn care	11 (44.0)
**Gestational age (upper limit)¶**	
≤ 32	18 (72.0)
≤ 34	3 (12.0)
Preterm (not specified)	4 (16.0)
**Number of babies in utero**	
Restriction or specification	3 (12.0)
No restriction or specification║	22 (88.0)
**Magnesium sulphate regimen mode**	
Intravenous	13 (52.0)
Intravenous and/or intramuscular	3 (12.0)
Not specified	9 (36.0)
Loading dose regimen**††	
4 g IV	12 (48.0)
4–6 g IV	4 (16.0)
10 g IM	3 (12.0)
Not specified	9 (36.0)
**Maintenance dose regimen**	
1 g/h IV	11 (44.0)
1–2 g/h IV	3 (12.0)
5 g/4–6 h IM	3 (12.0)
None	2 (8.0)
Not specified	9 (36.0)
Duration of maintenance dose**‡‡	
12 h/or until birth	2 (8.0)
24 h/or until birth	12 (48.0)
>24 h	1 (4.0)
Not reported	11 (44.0)
Nil (loading dose only)	1 (4.0)
Repeat treatment**	
Yes	2 (8.0)
No	3 (12.0)
Not specified	21 (84.0)
**Evidence cited**	
RCTs	3 (12.0)
2009 Cochrane systematic review	7 (28.0)
2015 WHO guideline	6 (24.0)
Other relevant systematic review(s)/meta-analysis(es) or guideline(s)	15 (60.0)

IM – intramuscular, IV – intravenous, PPROM – preterm prelabour rupture of membranes, PROM – prelabour rupture of membranes, RCT – randomised controlled trial, WHO – World Health Organization

*Countries were categorised by their World Bank income level on 13 January 2025.

†Includes one guideline that represented five countries: two lower-middle, three upper-middle and one high-income country.

‡Languages of publication: Indonesian and Spanish.

§Includes one guideline for the diagnosis and management of a small-for-gestational-age foetus and foetal growth restriction.

¶Gestational age categories: ≤32 includes <30 (consider up to 32–34), <30 or <32, <31, not specified or <32 (n = 1 each); ≤34 includes <32 or <34 for small for gestational age infants (n = 1).

║Includes one guideline that provides no specification, but a separate recommendation for twins.

**Total n is greater than the number of studies when multiple recommendations are made (total % can exceed 100).

††Includes guidelines recommending 4 g IV or 10 g IM (n = 1), and 4 g IV and 10g IM (n = 2).

‡‡24 h/until birth includes one guideline recommending medical dose until birth (no maximum duration).

### Research studies

**Figure 3:**
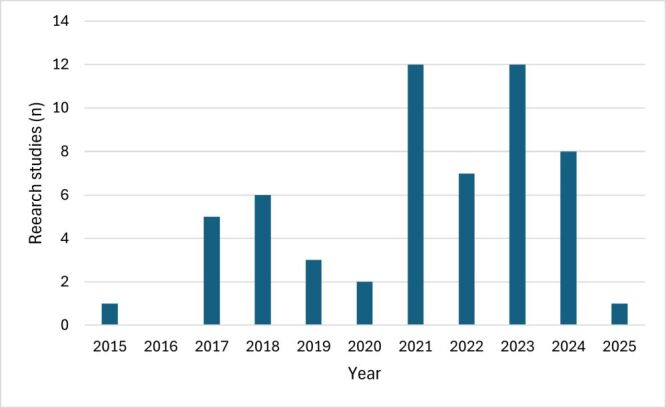
Research studies by year of publication.

Descriptive results were broadly similar whether including studies with full-text publications (n = 48) or all studies (n = 57) (**Table 1**; Table S1–3 in the **Online Supplementary Document**). The following results include all publications. The publications originated from 19 countries (**Figure 2**), most published 2020–2025 (**Figure 3**).

In our pragmatic categorisation of study scope and author-reported effects, without quality appraisal, the most common scope of the publications was the effects and/or safety of magnesium sulphate for neuroprotection (n = 38; 66.7%) [41,43,46–50,52–57,59–63,67,68,70–74,77–83,85,87–89,92,93]. The authors of 27 publications reported potential benefits (no harms) [41,48–50,52–57,59–63,71,72,74,78,79,81–83,85,87,89,93], while the authors of two studies reported no benefits but potential harms of magnesium sulphate. Only 12 publications reported a RCT design (21.1%) [36,38,39,47,49,57,73,77,82,83,85]. A smaller number of publications considered uptake of magnesium sulphate or a quality improvement programme (n = 10) [37,42,44, 45,48,64,75,76,90,91], with variable uptake reported by authors across relevant studies. The final publications focused on elucidating potential mechanisms of action (n = 5) [36,51,66,69,84] and comparisons of magnesium sulphate regimens (n = 4) [38–40,86] (**Table 1**; Table S4 in the **Online Supplementary Document**). These synthesised conclusions from authors must be held in tension with the descriptions of individual studies (Table S4 in the **Online Supplementary Document**), including small sample sizes, a lack of comparators, unclear or non-standard dosing, and non-prospective designs described as ‘trials.’

Given the variation in scope, the population characteristics in studies varied greatly. Studies enrolled women, infants, or women-infant pairs, with participant numbers ranging from 20–24 226. There were three large, retrospective cohort studies that included thousands of infants. These aimed to explore the neonatal effects of clinical practices (including magnesium sulphate) in large populations in China and South America [53,88,91]. However, most studies enrolled between 50 and 249 participants (n = 38) [36,39–41,43–46,48,49,51,52,54–63,66,69,71,73,74,76–80,83–86,90,93]. The upper limit of gestational age for women receiving magnesium sulphate also varied. After categorisation, the most common upper gestational age category was ≤32 weeks (n = 26 publications) [36,43,45,47,51,53,55,60–62,66–72,74,76,78,82,85,87,88,91,93] (**Table 1**; Table S3–4 in the **Online Supplementary Document**).

Magnesium sulphate regimens reported within studies most frequently included intravenous administration (n = 43) [36,38–41,43,44,47–52,54–60,62,63,66–74,77,78,80–87,92,93], particularly, a 4 g loading dose (n = 35) [38,39,41,43,47–50,52,54–63,66–69,71,72,74,80,82–87,92,93], and a maintenance dose of 1 g/h (n = 25) [36,43,47–50,57,58,60–63,68–72,74,80,82,83,85,86,92,93], continued for 24 hours or until birth (n = 18) [36,43,49,56–58,60,62,67,68,70,72,74,80,82–84,92], however there was heterogeneity between publications (**Table 1**; Table S4 in the **Online Supplementary Document**). Most studies [37,41–45,47–50,53–55,58,60–62,64,66,67,69,70,75–79,81,87,88,90,91] used a no magnesium sulphate comparator group, with six using a placebo [36,57,63,73,82,83] (**Table 1**; Table S4 in the **Online Supplementary Document**).

Outcomes reported by studies varied widely; some were clearly prespecified, while others were reported only in the study publications. Short-term foetal or neonatal (n = 44) [36,38–41,43,46–57,59–63,66–70,72–74,77,78,80,82–91] and maternal outcomes (n = 31) [36–38,40–42,44–48,50,51,55–62,64,75,76,78,79,82,84,90–92] were most common, along with death of the infant (n = 31) [38,41,43,46–50,52,53,55–57,59,61–63,67,69,70,72,74,78–80,82,83,86–88,90]. Longer-term follow-up was much less frequently reported, with CP described in only four studies [38,55,62,71] (**Table 1**; Table S4 in the **Online Supplementary Document**).

The evidence cited within the included publications varied. While one in five [45,52-54,56,58,59,61,82,85,92,93] cited the relevant 2015 WHO guideline [18], 40.4% [38,41,52–57,59,61,63,67–70,78,82–86,88,89] referenced the applicable 2009 Cochrane review [17] and 61.4% cited RCTs included within the Cochrane review [40,41,48–52,54–57,59–63,66–70,72–74,78,79,81–89]. Over two-thirds of publications referred to other relevant systematic reviews, meta-analyses or guidelines (68.4%) [38,40,41,43–46,48–50,52–56,58–63,66–70,72,75,78,80–83,85–88,91–93] (**Table 1**; Table S3 in the **Online Supplementary Document**).

### Guidelines

Of the 25 included guidelines, most were published by professional associations (n = 18) [94–96,98–100,102–104,108,109,111,112,114–118] or government bodies (n = 6) [97,101,105–107,113]. None were identified from low-income countries. A single international guideline focused on magnesium sulphate for neuroprotection was identified (International Federation of Gynaecology and Obstetrics) [114]. In all other cases, recommendations were included within broader guidelines (**Table 2**; Table S5 in the **Online Supplementary Document**).

Recommended magnesium sulphate regimens for foetal neuroprotection varied considerably. The upper limit of gestational age recommended varied from <30 weeks to 34 weeks, the most common category being ≤ 32 weeks (n = 18) [94,97–100,102,103,105,106,108–115,118]. There was generally no restriction or specification on the number of babies in utero (n = 22) [94–113,115,117]. One in three guidelines did not provide specific information about the recommended regimen. Of those providing a recommendation, intravenous administration was most common (n = 13) [94,95,98,100,101,105,106,109,112–115,118]. Intravenous loading doses (4 g) were most often recommended (n = 12) [95,100,101,105,107,111–116,118], though intramuscular doses were also endorsed [107,111,116]. Two guidelines included a regimen without a maintenance dose [101,109]. Most recommended 1 g/h intravenously (n = 11) [95,98,100,101,105,107,112–115,118]. A recommendation on repeat treatment was rarely made [98,106,116,118] (**Table 2**; Table S6 in the **Online Supplementary Document**).

Guidelines most commonly based their recommendations on other systematic review(s), meta-analysis(es) or guideline(s) (n = 15) [94–98,100,104–107,112–114,116,118], with some citing the relevant 2009 Cochrane review [17] (n = 7) [95,96,99,107,114,116,117] and 2015 WHO guideline [119] (n = 6) [99,100,105,110,116,117]. Six guidelines did not clearly cite published evidence [101–103,108,109,111,115] (**Table 2**; Table S5 in the **Online Supplementary Document**).

## DISCUSSION

In this scoping review, we systematically identified and mapped published research studies and clinical guidelines for the use of antenatal magnesium sulphate for preterm foetal neuroprotection in LMICs. We identified 57 research studies, all but one from middle-income countries including India, Turkey and China. Publications appeared to be increasing over time, with the greatest interest from 2021 onwards. One in five studies was an RCT; most aimed to assess effects (n = 9), with two comparing magnesium sulphate regimens. Of the 45 non-RCTs, most sought to assess effects (n = 29) or intervention uptake (n = 9). The studies primarily included short-term foetal/neonatal outcomes, with CP included in only four of 57 studies. We also identified 25 clinical guidelines; none originated from low-income countries, and most recommendations were published from 2022 onwards (Table S3–6 in the **Online Supplementary Document**).

To our knowledge, this is the first scoping review to map research and recommendations on the use of magnesium sulphate for foetal neuroprotection from LMICs. The inclusive methodology is not designed to assess effects; rather, it may complement the 2024 Cochrane review update on magnesium sulphate for foetal neuroprotection, which ultimately included studies from HICs only [25]. We noted variation across studies in the selected magnesium sulphate regimen, including selected dose, upper gestational age threshold, maintenance dose regimen and duration. While we did not formally assess efficacy or effectiveness, the authors of most studies reported benefits. These were mostly related to short-term foetal/neonatal outcomes rather than long-term neurodevelopment, including CP. However, we note that 10 of the 12 RCTs in our review were also identified by the 2024 Cochrane review. Six were deemed ‘awaiting classification’ (due to trustworthiness concerns) [48,57,63,73,82,83] and four were trial registrations [36,38,47,77]. Trustworthiness concerns in the Cochrane review of identified RCTs often related to publication without prospective registration and unclear methodology [25]. Of note, RCTs identified by our scoping review commonly included relatively small sample sizes and infrequent long-term outcome assessment (one RCT assessed CP), which may limit their influence in future systematic review updates. Taken together, these concerns may mean that some RCTs from our review may not ultimately be judged reliable enough for inclusion in future systematic review updates with effectiveness syntheses. It is acknowledged that resources, infrastructure, funding, and sample sizes are influenced by study context and affect research design. However, the primary outcome (*i.e.* CP) underpinning the WHO endorsement remains very sparsely documented in LMIC studies. This is a crucial evidence gap for context-specific policy.

The identification of 12 RCTs from LMICs in our scoping review highlights a clear desire for context-specific evidence for magnesium sulphate’s effects. For example, one such RCT stated, *‘*Although several developed countries have formulated guidelines for antenatal administration of magnesium sulphate for neuroprotection, only a few Indian studies have been conducted to assess the efficacy of the same in our population’ [83]. This desire is unsurprising, given that RCTs of perinatal interventions that proved effective in HICs have yielded mixed results in LMICs, where resources and infrastructure may be limited. For example, therapeutic hypothermia, widely recommended in HICs for treating neonatal encephalopathy, has shown mixed results in LMICs [120–122]. Until recent RCT evidence was established, the safety and efficacy of antenatal glucocorticoids prior to very preterm birth in low-resource countries were uncertain [123]. Large-scale population-based cohort studies may provide a viable alternative to RCTs for future context-specific evidence in LMICs.

The implementation of magnesium sulphate for foetal neuroprotection remains a challenge globally [20,124], likely exacerbated in LMICs, where additional systemic and health system barriers are common. We identified 10 studies reporting uptake or quality improvement in LMICs (six reporting moderate or high uptake), answering the WHO call to monitor the impact of their magnesium sulphate recommendation [18]. We did not identify any qualitative studies from LMICs focused on translating magnesium sulphate for foetal neuroprotection into practice, in line with another recent review of barriers and enablers to preterm-specific interventions in general [125]. Until such studies are undertaken, contextual factors identified in WHO guidance on interventions to improve preterm birth outcomes [19], and implementation studies of magnesium sulphate for pre-eclampsia or eclampsia from LMICs are worth considering [124,125]. These include health system capabilities, such as the availability of essential medicines, provider competence and confidence in administration, certainty of gestational age, and referral processes to manage women in preterm labour [18,125–127].

Barriers to the implementation of magnesium sulphate for foetal neuroprotection in HICs, identified in the aforementioned review [125], included health care professionals’ variable knowledge of benefits and harms, and inconsistent clinical guidelines and protocols [125]. We identified 25 guidelines (none from low-income countries) in this review. We deviated from our original protocol by including all self-identified national or international clinical guidelines, protocols, recommendations or standards, as we were focused on inclusivity and real-world recommendations for magnesium sulphate. However, we note that the methodological detail provided on guideline development is limited – it was often unclear whether guidelines were informed by systematic reviews, formal grading systems, or comprehensive stakeholder engagement – and we did not intend to evaluate quality. Recommendations within the 25 guidelines identified by our scoping review were largely consistent with the 2015 WHO guidance [18]. However, significant variation was observed, including differences in gestational age thresholds. In addition to variation, recommendations often lacked specificity regarding the magnesium sulphate regimen. One in three guidelines did not specify the mode of delivery, loading dose, maintenance dose, or the duration of the maintenance dose. Eighty-four percent did not specify whether repeat treatment was recommended. Similarly, a 2019 systematic review of guidelines on magnesium sulphate for foetal neuroprotection noted differences in the specifics of recommendations across the seven included guidelines [19]. Of note, all of the included guidelines were from HICs, recognising the value of our scoping review’s search extending beyond traditional databases to identify guidelines from LMICs [19]. This concerning variation and lack of specificity may contribute to confusion among health workers, to misdosing, or to missed treatments, ultimately affecting translation. Future guidelines should focus on the explicitness and alignment of recommendations, and on research into the mechanisms required to improve the global implementation of magnesium sulphate for foetal neuroprotection.

Our scoping review is not without limitations. We sought to be inclusive and captured research on magnesium sulphate regardless of whether this was the main or sole focus of the studies, but acknowledge that this inclusivity introduces significant limitations. As such, there is substantial heterogeneity in the study populations, outcomes, and methodologies of the included studies. For example, many studies, of relatively small samples, focused solely on evaluations of magnesium sulphate’s effects, while others, including the largest included study (> 24 000 infants) [91], involved an assessment of magnesium sulphate’s effects in the context of a broader evaluation of perinatal practice/interventions. By grouping studies and findings, the extent to which authors’ conclusions can be attributed to magnesium sulphate rather than co-interventions or system-level changes must be interpreted with caution, alongside detailed results (Table S3–4 in the **Online Supplementary Document**). While we chose to report the study authors’ main findings and overall conclusions regarding magnesium sulphate for foetal neuroprotection, and to make brief comments on the guidelines’ methodologies, we did not qualify these with judgements on the quality or certainty of the evidence (in keeping with scoping review guidance). We may have inadvertently misclassified findings through our pragmatic classification of scope and author conclusions, particularly by using uniform cut-offs and novel thresholds across studies with different designs and varying levels of reporting detail. Furthermore, although readers interested in specific outcomes may consult the supplementary files, our study design did not include reporting individual effect sizes for those outcomes.

We restricted inclusion to studies and guidelines published from 1 November 2015, hypothesising that research and guidelines would have burgeoned following the WHO endorsement. Thus, early experiences from LMICs may be under-represented. Furthermore, we included countries classified as LMIC by the World Bank as of the date of the final literature search. As the World Bank classification of countries changes, we may have excluded research or guidelines from countries that have recently transitioned, for example, from a middle-income country to an HIC.

While an extensive search was conducted as specified, it is possible that other national guidelines exist that were not captured, for example, if hosted on other government portals not accessible to the public, by professional bodies outside of obstetrics and gynaecology, or on non-English websites with limited discoverability. In particular, the apparent absence of guidelines from low-income countries may reflect limitations in online availability or searchability. Finally, we pragmatically limited our search to national and international guidelines, meaning we could not assess potential variation in guidance at the regional, local, and hospital levels.

Optimising outcomes for infants born preterm, including the use of magnesium sulphate for foetal neuroprotection, requires ongoing concerted efforts by the global community. To date, no large RCT has assessed the efficacy or alternative regimens of magnesium sulphate for the prevention of death or CP in an LMIC [25,128]. Given the availability of high-certainty evidence from HICs [25], regimen comparisons in future RCTs may be preferred, prioritising, for example, intramuscular dosing or loading-dose-only regimens [128]. Both included RCTs comparing regimens in this review evaluated a 4 g loading dose only *vs*. a 4 g loading dose combined with a 1 g/h maintenance dose. Pharmacokinetic modelling of existing data from HIC studies may also offer novel insights into alternate dosing regimens to maximise neuroprotection [128].

Other gaps identified include research and guidelines from low-income countries, as well as qualitative studies of barriers and facilitators to implementation from LMICs. In low-resource contexts in particular, there are myriad challenges related to research capacity, funding, and global health priorities. Faced with limited resources and competing opportunities and priorities, it is not unexpected that evidence generation on antenatal magnesium sulphate has not progressed at the same pace as in HICs. A lack of guidelines from low-income countries, in particular, is noted – in addition to the aforementioned resource limitations, contributing factors may include limited specialist societies, reliance on general WHO guidance, or reliance on subnational protocols. That being said, in both HICs [19] and LMICs, specific recommendations regarding the use of magnesium sulphate vary across guidelines. A forthcoming update to the magnesium sulphate for foetal neuroprotection individual participant data meta-analysis may help address outstanding uncertainties regarding which women to treat, when, and how [25]. It is hoped that this will also inform future WHO guidance and may, in turn, support more consistent recommendations across the globe.

## CONCLUSIONS

In this scoping review, we broadly described the landscape of magnesium sulphate for foetal neuroprotection research and guidelines from LMICs since the WHO 2015 recommendation. It identified a sizeable number of heterogeneous studies, often with small sample sizes and short-term outcomes. Therefore, despite the sheer volume of data, the core evidence on CP and long-term outcomes in LMICs remains minimal. While promising, research from LMICs remains fragmented, with many small, heterogeneous studies rather than a coherent, high-quality evidence base. Further context-specific research is warranted, building on the 2024 Cochrane review. This may, for example, include a focus on regimen comparisons in RCTs, larger-scale evaluations of ‘real-world’ impact in non-RCTs, and national/local implementation studies including qualitative components. Guidelines identified mainly provided recommendations aligned with 2015 WHO guidance, though variation existed, and clarity regarding specific aspects of the recommendations was often lacking. Collaborative efforts to generate robust evidence and relevant guidelines, including for antenatal magnesium sulphate for foetal neuroprotection, are critical to improving outcomes for preterm infants globally.

## Additional material


Online Supplementary Document
