# Supplementary material for: Antenatal magnesium sulphate for preterm foetal neuroprotection in low- and middle-income countries: a scoping review of research studies and guidelines
Source: J Glob Health. 2026 Mar 20;16:04088. doi: 10.7189/jogh.16.04088 (PMC13003896; doi:10.7189/jogh.16.04088)
Supplement: Online Supplementary Document [file jogh-16-04088-s001.pdf]

Supplement to: Goldsmith S, Karim T, McIntyre S, Rumbold A, Malhotra A, Khandaker G, Arya S, Shepherd E. Antenatal magnesium sulphate for preterm fetal neuroprotection in low- and middle-income countries: a scoping review of research studies and guidelines. J Glob Health. 2026;16:04088.

S1 Appendix. Database and register search strategies

CENTRAL search strategy and results

#	Search strategy (25 May 2023)	Results
1	MeSH descriptor: [Magnesium Sulfate] this term only	1189
2	(magnesium-sulfate or magnesium-sulphate or MgSO4):ti,ab,kw	3353
3	MeSH descriptor: [Pregnancy] explode all trees	31028
4	MeSH descriptor: [Pregnancy Complications] explode all trees	15881
5	MeSH descriptor: [Prenatal Care] this term only	2119
6	MeSH descriptor: [Perinatal Care] this term only	309
7	MeSH descriptor: [Maternal Exposure] this term only	105
8	MeSH descriptor: [Infant, Premature] explode all trees	4871
9	MeSH descriptor: [Cerebral Palsy] this term only	2116
10	(pregnan* or labor or laboring or labour* or antepart* or prenatal* or antenatal* or perinatal* or intranatal* or obstetric* or intrapart* or preterm or prematur* or fetus* or fetal* or foetus* or foetal* or neuroprotect* or cerebral palsy or cerebral palsies):ti,ab,kw	129771
11	(afghan* OR africa* OR albania* OR algeria* OR angola* OR antigua* OR barbuda* OR argentin* OR armenia* OR aruba* OR azerbaijan* OR bahrain* OR bangladesh* OR bengal* OR bangal* OR barbados* OR barbadian* OR bajan OR bajans OR belarus* OR belorus* OR byelarus* OR byelorus* OR belize* OR benin* OR dahomey OR bhutan* OR bolivia* OR bosnia* OR herzegovin* OR botswan* OR batswan* OR bechuanaland OR brazil* OR brasil* OR bulgaria* OR burkina* OR burkinese* OR upper-volta* OR burundi* OR urundi* OR cabo-verde* OR cape-verde* OR cambodia* OR kampuchea* OR khmer* OR cameroon* OR cameroun* OR ubangi-shari* OR chad* OR chile* OR china* OR chinese OR colombia* OR comoro* OR comore* OR comorian* OR mayotte* OR congo* OR zaire* OR costa-rica* OR (cote* AND *ivoir*) OR ivory-coast* OR ivorian* OR croatia* OR cuba* OR cyprus* OR cypriot* OR czech* OR djibouti* OR french-somaliland* OR dominica* OR ecuador* OR egypt* OR united-arab-republic* OR el-salvador* OR salvadoran* OR guinea* OR equatoguinea* OR eritrea* OR estonia* OR eswatini* OR swaziland* OR swazi* OR swati* OR ethiopia* OR fiji* OR gabon* OR gabonese* OR gabonaise* OR gambia* OR ((georgia OR georgian OR georgians) NOT (atlanta OR california OR florida)) OR ghana* OR gibraltar* OR greece* OR greek* OR grecian* OR grenada* OR grenadian* OR guam* OR guatemala* OR guyana* OR guiana* OR guyanese* OR haiti* OR hispaniola* OR hondura* OR hungary* OR hungarian* OR india* OR indonesia* OR iran* OR iraq* OR isle-of-man* OR jamaica* OR jordan* OR kazakh* OR kenya* OR karabati* OR korea* OR kosovo* OR kosova* OR kyrgyz* OR kirgiz* OR kirghiz* OR laos OR lao OR laotian* OR latvia* OR lebanon* OR lebanese* OR lesotho* OR lesothan* OR lesothonian* OR basutoland* OR mosotho* OR basotho* OR liberia* OR libya* OR jamahiriya* OR lithuania* OR macedonia* OR madagasca* OR malagasy* OR malawi* OR nyasaland* OR malaysia* OR malay-federation OR malaya-federation OR malayan-federation OR maldives* OR maldivian* OR indian-ocean* OR mali* OR malta* OR maltese* OR micronesia* OR marshallese* OR kiribati* OR marshall-island* OR nauru OR nauran OR nauruans OR nauran* OR mariana OR marianas OR palau OR paluan* OR tuvalu* OR mauritania* OR mauritan* OR mauritius* OR mexico* OR mexican* OR moldova* OR moldovia* OR mongol* OR montenegr* OR morocco* OR moroccan* OR ifni OR mozambique* OR mozambican* OR myanmar* OR burma* OR burmese OR namibia* OR nepal* OR new-caledonia* OR netherlands-antill* OR nicaragua* OR niger* OR oman* OR pakistan* OR palestin* OR gaza* OR west-bank* OR panama* OR paraguay* OR peru* OR philippine* OR philipine* OR phillipine* OR phillippine* OR filipino* OR filipina* OR poland* OR polish OR pole OR poles OR portugal* OR portuguese OR puerto-ric* OR romania* OR russia* OR ussr* OR soviet* OR rwanda* OR rwandese OR ruanda* OR ruandese OR samoa* OR navigator-island* OR pacific-island* OR polynesia* OR sao-tome* OR santomean* OR saudi-arabia* OR saudi OR saudis OR senegal* OR serbia* OR seychell* OR sierra-leone* OR slovak* OR sloven* OR melanesia* OR solomon-island* OR norfolk-island*	211709

	OR somali* OR sri-lanka* OR ceylon* OR saint-kitts* OR st-kitts* OR kittian* OR nevisian* OR saint-lucia* OR st-lucia* OR saint-vincent* OR st-vincent* OR vincentian* OR grenadine* OR sudan* OR surinam* OR syria* OR tajik* OR tadjik* OR tadzhik* OR tanzania* OR tanganyika* OR thai* OR timor-leste* OR east-timor* OR timorese* OR togo* OR tonga* OR trinidad* OR tobago* OR tunisia* OR turkiy* OR turkey* OR turk OR turks OR turkish OR turkmen* OR uganda* OR ukrain* OR uruguay* OR uzbek* OR vanuatu* OR new-hebrides OR venezuela* OR vietnam* OR viet-nam* OR yemen* OR yugoslav* OR zambia* OR zimbabwe* OR rhodesia* OR arab-countr* OR arabic-countr* OR middle-east* OR global-south OR sahara* OR subsahara* OR magreb* OR maghrib* OR west-indies* OR caribbean* OR central-america* OR latin-america* OR south-america* OR central-asia* OR north-asia* OR northern-asia* OR southeastern-asia* OR south-eastern-asia* OR southeast-asia* OR south-east-asia* OR west-asia* OR western-asia* OR east-europe* OR eastern-europe* OR developing-countr* OR developing-nation* OR developing-population* OR developing-world OR less-developed-countr* OR less-developed-nation* OR less-developed-world OR lesser-developed-countr* OR lesser-developed-nation* OR lesser-developed-world OR under-developed-countr* OR under-developed-nation* OR under-developed-world OR underdeveloped-countr* OR underdeveloped-nation* OR underdeveloped-world OR middle-income-countr* OR middle-income-nation* OR middle-income-population* OR low-income-countr* OR low-income-nation* OR low-income-population* OR lower-income-countr* OR lower-income-nation* OR lower-income-population* OR underserved-countr* OR underserved-nation* OR underserved-population* OR under-served-population* OR under-served-nation* OR under-served-population* OR deprived-countr* OR deprived-population* OR high-burden-countr* OR high-burden-nation* OR countdown-countr* OR countdown-nation* OR poor-countr* OR poor-nation* OR poor-population* OR poor-world OR poorer-countr* OR poorer-nation* OR poorer-population* OR poorer-world OR developing-econom* OR less developed-econom* OR underdeveloped-econom* OR under-developed-econom* OR middle-income-econom* OR low-income-econom* OR lower-income-econom* OR low-gdp OR low-gnp OR low-gross-domestic OR low-gross-national OR lower-gdp OR lower-gnp OR lower-gross-domestic OR lower-gross-national OR lmic OR lmics OR third-world OR lami-countr* OR transitional-countr* OR emerging-econom* OR emerging-nation*):ti,ab,kw	
12	#1 or #2	3353
13	#3 or #4 or #5 or #6 or #7 or #8 or #9 or #10	131022
14	#11 and #12 and #13 *with Publication Year from 2015 to present, in Trials*	90
Top up of search strategy (13 January 2025)		21
**Total**		111

Ovid Embase search strategy and results

#	Search strategy (25 May 2023)	Results
1	Magnesium Sulfate/	19365
2	(magnesium sulfate OR magnesium sulphate OR MgSO4).mp.	22105
3	OR/1-2	2210
4	exp Pregnancy/	772175
5	exp Pregnancy Complications/	159000
6	Prenatal Care/	49649
7	Perinatal Care/	15925
8	Maternal Exposure/	4273
9	Exp Infant, Premature/	127009
10	Cerebral Palsy/	44335
11	pregnan* OR labor OR laboring OR labour* OR antepart* OR prenatal* OR antenatal* OR perinatal* OR intranatal* OR obstetric* OR intrapart* OR preterm OR prematur* OR fetus* OR fetal* OR foetus* OR foetal* OR neuroprotect* OR cerebral palsy OR cerebral palsies	2084494
12	OR/4-11	2094821

13	(afghan* OR africa* OR albania* OR algeria* OR angola* OR antigua* OR barbuda* OR argentin* OR armenia* OR aruba* OR azerbaijan* OR bahrain* OR bangladesh* OR bengal* OR bangal* OR barbados* OR barbadian* OR bajan OR bajans OR belarus* OR belorus* OR byelarus* OR byelorus* OR belize* OR benin* OR dahomey OR bhutan* OR bolivia* OR bosnia* OR herzegovin* OR botswan* OR batswan* OR bechuanaland* OR brazil* OR brasil* OR bulgaria* OR burkina* OR burkinese* OR upper volta* OR burundi* OR urundi* OR cabo verde* OR cape verde* OR cambodia* OR kampuchea* OR khmer* OR cameroon* OR cameroun* OR ubangi shari* OR chad* OR chile* OR china* OR chinese OR colombia* OR comoro* OR comore* OR comorian* OR mayotte* OR congo* OR zaire* OR costa rica* OR "cote d'ivoir*" OR "cote d' ivoir*" OR cote divoir* OR cote d ivoir* OR ivory coast* OR ivorian* OR croatia* OR cuba OR cuban OR cubans OR "cuba's" OR cyprus* OR cypriot* OR czech* OR djibouti* OR french somaliland* OR dominica* OR ecuador* OR egypt* OR united arab republic* OR el salvador* OR salvadoran* OR guinea* OR equatoguinea* OR eritrea* OR estonia* OR eswatini* OR swaziland* OR swazi* OR swati* OR ethiopia* OR fiji* OR gabon* OR gabonese* OR gabonaise* OR gambia* OR ((georgia OR georgian OR georgians) NOT (atlanta OR california OR florida)) OR ghana* OR gibraltar* OR greece* OR greek* OR grecian* OR grenada* OR grenadian* OR guam* OR guatemala* OR guyana* OR guiana* OR guyanese* OR haiti* OR hispaniola* OR hondura* OR hungary* OR hungarian* OR india* OR indonesia* OR iran* OR iraq* OR isle of man* OR jamaica* OR jordan* OR kazakh* OR kenya* OR karabati* OR korea* OR kosovo* OR kosova* OR kyrgyz* OR kirgiz* OR kirghiz* OR laos OR lao OR laotian* OR latvia* OR lebanon* OR lebanese* OR lesotho* OR lesothan* OR lesothonian* OR basutoland* OR mosotho* OR basotho* OR liberia* OR libya* OR jamahiriya* OR lithuania* OR macedonia* OR madagasca* OR malagasy* OR malawi* OR nyasaland* OR malaysia* OR malay* federation OR maldives* OR maldivian* OR indian ocean OR mali OR malian* OR "mali's" OR malta OR maltese* OR "malta's" OR micronesia* OR marshallese* OR kiribati* OR marshall island* OR nauru OR nauran OR nauruans OR "naurian's" OR mariana OR marianas OR palau OR paluan* OR tuvalu* OR mauritania* OR mauritan* OR mauritius* OR mexico* OR mexican* OR moldova* OR moldovia* OR mongol* OR montenegr* OR morocco* OR moroccan* OR ifni OR mozambique* OR mozambican* OR myanmar* OR burma* OR burmese OR namibia* OR nepal* OR new caledonia* OR netherlands antill* OR nicaragua* OR niger* OR oman OR omani OR omanis OR "oman's" OR pakistan* OR palestin* OR gaza* OR west bank* OR panama* OR paraguay* OR peru OR peruvian* OR "peru's" OR philippine* OR philipine* OR phillipine* OR phillippine* OR filipino* OR filipina* OR poland* OR polish OR pole OR poles OR portugal* OR portuguese OR puerto ric* OR romania* OR russia* OR ussr* OR soviet* OR rwanda* OR rwandese OR ruanda* OR ruandese OR samoa* OR navigator island* OR pacific island* OR polynesia* OR "sao tome and principe*" OR sao tomean* OR santomean* OR saudi arabia* OR saudi OR saudis OR senegal* OR serbia* OR seychell* OR sierra leone* OR slovak* OR sloven* OR melanesia* OR solomon island* OR norfolk island* OR somali* OR sri lanka* OR ceylon* OR "saint kitts and nevis*" OR "st kitts and nevis*" OR kittian* OR nevisian* OR saint lucia* OR st lucia* OR saint vincent* OR st vincent* OR vincentian* OR grenadine* OR sudan* OR surinam* OR syria* OR tajik* OR tadjik* OR tadzhik* OR tanzania* OR tanganyika* OR thai* OR timor leste* OR east timor* OR timorese* OR togo OR togoles* OR "togo's" OR tonga* OR trinidad* OR tobago* OR tunisia* OR turkiy* OR turkey* OR turk OR turks OR turkish OR turkmen* OR uganda* OR ukrain* OR uruguay* OR uzbek* OR vanuatu* OR new hebrides* OR venezuela* OR vietnam* OR viet nam* OR yemen* OR yugoslav* OR zambia* OR zimbabwe* OR rhodesia* OR arab* countr* OR middle east* OR global south OR sahara* OR subsahara* OR magreb* OR maghrib* OR west indies* OR caribbean* OR central america* OR latin america* OR south america* OR central asia* OR north asia* OR northern asia* OR southeastern asia* OR south eastern asia* OR southeast asia* OR south east asia* OR west asia* OR western asia* OR east europe* OR eastern europe* OR developing countr* OR developing nation* OR developing population* OR developing world OR less developed countr* OR less developed nation* OR less developed world OR lesser developed countr*	3837326

	OR lesser developed nation* OR lesser developed world OR under developed countr* OR under developed nation* OR under developed world OR underdeveloped countr* OR underdeveloped nation* OR underdeveloped world OR middle income countr* OR middle income nation* OR middle income population* OR low income countr* OR low income nation* OR low income population* OR lower income countr* OR lower income nation* OR lower income population* OR underserved countr* OR underserved nation* OR underserved population* OR under served population* OR under served nation* OR under served population* OR deprived countr* OR deprived population* OR high burden countr* OR high burden nation* OR countdown countr* OR countdown nation* OR poor countr* OR poor nation* OR poor population* OR poor world OR poorer countr* OR poorer nation* OR poorer population* OR poorer world OR developing econom* OR less developed econom* OR underdeveloped econom* OR under developed econom* OR middle income econom* OR low income econom* OR lower income econom* OR low gdp OR low gnp OR low gross domestic OR low gross national OR lower gdp OR lower gnp OR lower gross domestic OR lower gross national OR lmic OR lmics OR third world OR lami countr* OR transitional countr* OR emerging econom* OR emerging nation*).ti,ab,hw,kf.	
14	3 and 12 and 13	1026
15	Limit 14 to yr="2015 -Current"	525
Top up of search strategy (13 January 2025)		175
**Total**		700

Ovid MEDLINE search strategy and results

#	Search strategy (25 May 2023)	Results
1	Magnesium Sulfate/	9816
2	(magnesium sulfate OR magnesium sulphate OR MgSO4).mp.	5511
3	OR/1-2	9816
4	exp Pregnancy/	1001959
5	exp Pregnancy Complications/	472839
6	Prenatal Care/	32352
7	Perinatal Care/	5299
8	Maternal Exposure/	11069
9	Exp Infant, Premature/	64631
10	Cerebral Palsy/	23533
11	(pregnan* OR labor OR laboring OR labour* OR antepart* OR prenatal* OR antenatal* OR perinatal* OR intranatal* OR obstetric* OR intrapart* OR preterm OR prematur* OR fetus* OR fetal* OR foetus* OR foetal* OR neuroprotect* OR cerebral palsy or cerebral palsies).mp.	1750839
12	OR/4-11	1778530
13	(afghan* OR africa* OR albania* OR algeria* OR angola* OR antigua* OR barbuda* OR argentin* OR armenia* OR aruba* OR azerbaijan* OR bahrain* OR bangladesh* OR bengal* OR bangal* OR barbados* OR barbadian* OR bajan OR bajans OR belarus* OR belorus* OR byelarus* OR byelorus* OR belize* OR benin* OR dahomey OR bhutan* OR bolivia* OR bosnia* OR herzegovin* OR botswan* OR batswan* OR bechuanaland* OR brazil* OR brasil* OR bulgaria* OR burkina* OR burkinese* OR upper volta* OR burundi* OR urundi* OR cabo verde* OR cape verde* OR cambodia* OR kampuchea* OR khmer* OR cameroon* OR cameroun* OR ubangi shari* OR chad* OR chile* OR china* OR chinese OR colombia* OR comoro* OR comore* OR comorian* OR mayotte* OR congo* OR zaire* OR costa rica* OR "cote d'ivoir*" OR "cote d' ivoir*" OR cote divoir* OR cote d ivoir* OR ivory coast* OR ivorian* OR croatia* OR cuba OR cuban OR cubans OR "cuba's" OR cyprus* OR cypriot* OR czech* OR djibouti* OR french somaliland* OR dominica* OR ecuador* OR egypt* OR united arab republic* OR el salvador* OR salvadoran* OR guinea* OR equatoguinea* OR eritrea* OR estonia* OR eswatini* OR swaziland* OR swazi* OR swati* OR ethiopia* OR fiji* OR gabon* OR gabonese* OR gabonaise* OR gambia* OR ((georgia OR georgian OR georgians) NOT (atlanta OR california OR florida)) OR ghana* OR gibraltar* OR greece* OR	3160177

	greek* OR grecian* OR grenada* OR grenadian* OR guam* OR guatemala* OR guyana* OR guiana* OR guyanese* OR haiti* OR hispaniola* OR hondura* OR hungary* OR hungarian* OR india* OR indonesia* OR iran* OR iraq* OR isle of man* OR jamaica* OR jordan* OR kazakh* OR kenya* OR karabati* OR korea* OR kosovo* OR kosova* OR kyrgyz* OR kirgiz* OR kirghiz* OR laos OR lao OR laotian* OR latvia* OR lebanon* OR lebanese* OR lesotho* OR lesothan* OR lesothonian* OR basutoland* OR mosotho* OR basotho* OR liberia* OR libya* OR jamahiriya* OR lithuania* OR macedonia* OR madagasca* OR malagasy* OR malawi* OR nyasaland* OR malaysia* OR malay* federation OR maldives* OR maldivian* OR indian ocean OR mali OR malian* OR "mali's" OR malta OR maltese* OR "malta's" OR micronesia* OR marshallese* OR kiribati* OR marshall island* OR nauru OR nauran OR nauruans OR "naurian's" OR mariana OR marianas OR palau OR paluan* OR tuvalu* OR mauritania* OR mauritan* OR mauritius* OR mexico* OR mexican* OR moldova* OR moldovia* OR mongol* OR montenegr* OR morocco* OR moroccan* OR ifni OR mozambique* OR mozambican* OR myanmar* OR burma* OR burmese OR namibia* OR nepal* OR new caledonia* OR netherlands antill* OR nicaragua* OR niger* OR oman OR omani OR omanis OR "oman's" OR pakistan* OR palestin* OR gaza* OR west bank* OR panama* OR paraguay* OR peru OR peruvian* OR "peru's" OR philippine* OR philipine* OR phillipine* OR phillippine* OR filipino* OR filipina* OR poland* OR polish OR pole OR poles OR portugal* OR portuguese OR puerto ric* OR romania* OR russia* OR ussr* OR soviet* OR rwanda* OR rwandese OR ruanda* OR ruandese OR samoa* OR navigator island* OR pacific island* OR polynesia* OR "sao tome and principe*" OR sao tomean* OR santomean* OR saudi arabia* OR saudi OR saudis OR senegal* OR serbia* OR seychell* OR sierra leone* OR slovak* OR sloven* OR melanesia* OR solomon island* OR norfolk island* OR somali* OR sri lanka* OR ceylon* OR "saint kitts and nevis*" OR "st kitts and nevis*" OR kittian* OR nevisian* OR saint lucia* OR st lucia* OR saint vincent* OR st vincent* OR vincentian* OR grenadine* OR sudan* OR surinam* OR syria* OR tajik* OR tadjik* OR tadzhik* OR tanzania* OR tanganyika* OR thai* OR timor leste* OR east timor* OR timorese* OR togo OR togoles* OR "togo's" OR tonga* OR trinidad* OR tobago* OR tunisia* OR turkiy* OR turkey* OR turk OR turks OR turkish OR turkmen* OR uganda* OR ukrain* OR uruguay* OR uzbek* OR vanuatu* OR new hebrides* OR venezuela* OR vietnam* OR viet nam* OR yemen* OR yugoslav* OR zambia* OR zimbabwe* OR rhodesia* OR arab* countr* OR middle east* OR global south OR sahara* OR subsahara* OR magreb* OR maghrib* OR west indies* OR caribbean* OR central america* OR latin america* OR south america* OR central asia* OR north asia* OR northern asia* OR southeastern asia* OR south eastern asia* OR southeast asia* OR south east asia* OR west asia* OR western asia* OR east europe* OR eastern europe* OR developing countr* OR developing nation* OR developing population* OR developing world OR less developed countr* OR less developed nation* OR less developed world OR lesser developed countr* OR lesser developed nation* OR lesser developed world OR under developed countr* OR under developed nation* OR under developed world OR underdeveloped countr* OR underdeveloped nation* OR underdeveloped world OR middle income countr* OR middle income nation* OR middle income population* OR low income countr* OR low income nation* OR low income population* OR lower income countr* OR lower income nation* OR lower income population* OR underserved countr* OR underserved nation* OR underserved population* OR under served population* OR under served nation* OR under served population* OR deprived countr* OR deprived population* OR high burden countr* OR high burden nation* OR countdown countr* OR countdown nation* OR poor countr* OR poor nation* OR poor population* OR poor world OR poorer countr* OR poorer nation* OR poorer population* OR poorer world OR developing econom* OR less developed econom* OR underdeveloped econom* OR under developed econom* OR middle income econom* OR low income econom* OR lower income econom* OR low gdp OR low gnp OR low gross domestic OR low gross national OR lower gdp OR lower gnp OR lower gross domestic OR lower gross national OR lmic OR lmics OR third world OR lami countr* OR transitional countr* OR emerging econom* OR emerging nation*).ti,ab,hw,kf.	

14	3 and 12 and 13	395
15	Limit 14 to yr="2015 -Current"	175
Top up of search strategy (13 January 2025)		59
**Total**		234

Global Index Medicus search strategy and results

**Search strategy (25 May 2023)**	**Results**
(tw:(("magnesium sulfate" OR "magnesium sulphate" OR MgSO4))) AND (tw:((pregnan* OR labor OR laboring OR labour* OR antepart* OR prenatal* OR antenatal* OR perinatal* OR intranatal* OR obstetric* OR intrapart* OR preterm OR prematur* OR fetus* OR fetal* OR foetus* OR foetal* OR neuroprotect* OR "cerebral palsy" or "cerebral palsies")))<br>[restricted to 2015-]	119
Top up of search strategy (13 January 2025)	29
**Total**	148

Trip Medical Database search strategy and results

**Search strategy (25 May 2023)**	**Results**
("magnesium sulfate" OR "magnesium sulphate" OR MgSO4) AND (pregnan* OR labor OR laboring OR labour* OR antepart* OR prenatal* OR antenatal* OR perinatal* OR intranatal* OR obstetric* OR intrapart* OR preterm OR prematur* OR fetus* OR fetal* OR foetus* OR foetal* OR neuroprotect* OR "cerebral palsy" or "cerebral palsies")<br>[restricted to 2015-; LMIC sensitive]	560
**Total**	560

PQDT Global search strategy and results

**Search strategy (25 May 2023)**	**Results**
noft("magnesium sulfate" OR "magnesium sulphate" OR MgSO4) AND fulltext(pregnan* OR labor OR laboring OR labour* OR antepart* OR prenatal* OR antenatal* OR perinatal* OR intranatal* OR obstetric* OR intrapart* OR preterm OR prematur* OR fetus* OR fetal* OR foetus* OR foetal* OR neuroprotect* OR "cerebral palsy" OR "cerebral palsies")<br>[restricted to 2015-]	38
Top up of search strategy (13 January 2025)	2
**Total**	40

Google Scholar search strategy and results

**Search strategy (25 May 2023)**	**Results**
All of the words: magnesium sulfate<br>With at least one of the words: pregnant pregnancy pregnancies labor labour laboring labouring antepartum prenatal prenatally antenatal antenatally perinatal perinatally intranatal intranatally obstetric obstetrics intrapartum preterm premature prematurely fetus fetuses fetal fetally foetus foetuses foetal foetally neuroprotective neuroprotection "cerebral palsy" "cerebral palsies"<br><br>(also searched with magnesium sulphate, and MgSO4)<br>[restricted to 2015-; all words in title of the article]	298
Top up of search strategy (13 January 2025)	112
**Total**	410

ClinicalTrials.gov search strategy and results

**Search strategy (25 May 2023)**	**Results**
magnesium sulfate OR magnesium sulphate OR MgSO4 (intervention/treatment)	18

pregnant OR pregnancy OR labor OR laboring OR labour OR labouring OR antepartum OR prenatal OR antenatal OR perinatal OR intranatal OR obstetric OR intrapartum OR preterm OR premature OR fetus OR fetal OR foetus OR foetal OR neuroprotection OR cerebral palsy (title) [first posted from 01/01/2015]	
Top up of search strategy (13 January 2025)	3
**Total**	21

ICTRP search strategy and results

Search strategy (25 May 2023)	Results
pregnan* OR labor OR laboring OR labour* OR antepart* OR prenatal* OR antenatal* OR perinatal* OR intranatal* OR obstetric* OR intrapart* OR preterm OR prematur* OR fetus* OR fetal* OR foetus* OR foetal* OR neuroprotect* OR “cerebral palsy” or “cerebral palsies” (in title)<br>AND<br>"magnesium sulfate" OR "magnesium sulphate" OR MgSO4 (in intervention)<br>[recruitment status: ALL; date of registration from 1/01/2015]	67
Top up of search strategy (13 January 2025)	13
**Total**	80

S1 Table. Preferred Reporting Items for Systematic reviews and Meta-Analyses extension for Scoping Reviews (PRISMA-ScR) Checklist

SECTION	ITEM	PRISMA-ScR CHECKLIST ITEM	REPORTED ON PAGE #
**TITLE**			
Title	1	Identify the report as a scoping review.	1
**ABSTRACT**			
Structured summary	2	Provide a structured summary that includes (as applicable): background, objectives, eligibility criteria, sources of evidence, charting methods, results, and conclusions that relate to the review questions and objectives.	2
**INTRODUCTION**			
Rationale	3	Describe the rationale for the review in the context of what is already known. Explain why the review questions/objectives lend themselves to a scoping review approach.	3
Objectives	4	Provide an explicit statement of the questions and objectives being addressed with reference to their key elements (e.g., population or participants, concepts, and context) or other relevant key elements used to conceptualize the review questions and/or objectives.	3
**METHODS**			
Protocol and registration	5	Indicate whether a review protocol exists; state if and where it can be accessed (e.g., a Web address); and if available, provide registration information, including the registration number.	2, 4
Eligibility criteria	6	Specify characteristics of the sources of evidence used as eligibility criteria (e.g., years considered, language, and publication status), and provide a rationale.	4
Information sources*	7	Describe all information sources in the search (e.g., databases with dates of coverage and contact with authors to identify additional sources), as well as the date the most recent search was executed.	5-6
Search	8	Present the full electronic search strategy for at least 1 database, including any limits used, such that it could be repeated.	4, S1 Appendix
Selection of sources of evidence†	9	State the process for selecting sources of evidence (i.e., screening and eligibility) included in the scoping review.	5
Data charting process‡	10	Describe the methods of charting data from the included sources of evidence (e.g., calibrated forms or forms that have been tested by the team before their use, and whether data charting was done independently or in duplicate) and any processes for obtaining and confirming data from investigators.	5
Data items	11	List and define all variables for which data were sought and any assumptions and simplifications made.	5
Critical appraisal of individual sources of evidence§	12	If done, provide a rationale for conducting a critical appraisal of included sources of evidence; describe the methods used and how this information was used in any data synthesis (if appropriate).	N/A

SECTION	ITEM	PRISMA-ScR CHECKLIST ITEM	REPORTED ON PAGE #
Synthesis of results	13	Describe the methods of handling and summarizing the data that were charted.	5
**RESULTS**			
Selection of sources of evidence	14	Give numbers of sources of evidence screened, assessed for eligibility, and included in the review, with reasons for exclusions at each stage, ideally using a flow diagram.	5, Fig1
Characteristics of sources of evidence	15	For each source of evidence, present characteristics for which data were charted and provide the citations.	5, Fig2, Fig3, Table 1, S3 Table
Critical appraisal within sources of evidence	16	If done, present data on critical appraisal of included sources of evidence (see item 12).	N/A
Results of individual sources of evidence	17	For each included source of evidence, present the relevant data that were charted that relate to the review questions and objectives.	S3-6 Tables, 5-11
Synthesis of results	18	Summarize and/or present the charting results as they relate to the review questions and objectives.	5-11
**DISCUSSION**			
Summary of evidence	19	Summarize the main results (including an overview of concepts, themes, and types of evidence available), link to the review questions and objectives, and consider the relevance to key groups.	11-12
Limitations	20	Discuss the limitations of the scoping review process.	12
Conclusions	21	Provide a general interpretation of the results with respect to the review questions and objectives, as well as potential implications and/or next steps.	13
**FUNDING**			
Funding	22	Describe sources of funding for the included sources of evidence, as well as sources of funding for the scoping review. Describe the role of the funders of the scoping review.	14

JBI = Joanna Briggs Institute; PRISMA-ScR = Preferred Reporting Items for Systematic reviews and Meta-Analyses extension for Scoping Reviews.
* Where *sources of evidence* (see second footnote) are compiled from, such as bibliographic databases, social media platforms, and Web sites.
† A more inclusive/heterogeneous term used to account for the different types of evidence or data sources (e.g., quantitative and/or qualitative research, expert opinion, and policy documents) that may be eligible in a scoping review as opposed to only studies. This is not to be confused with *information sources* (see first footnote).
‡ The frameworks by Arksey and O'Malley (6) and Levac and colleagues (7) and the JBI guidance (4, 5) refer to the process of data extraction in a scoping review as data charting.
§ The process of systematically examining research evidence to assess its validity, results, and relevance before using it to inform a decision. This term is used for items 12 and 19 instead of "risk of bias" (which is more applicable to systematic reviews of interventions) to include and acknowledge the various sources of evidence that may be used in a scoping review (e.g., quantitative and/or qualitative research, expert opinion, and policy document).

From: Tricco AC, Lillie E, Zarin W, O'Brien KK, Colquhoun H, Levac D, et al. PRISMA Extension for Scoping Reviews (PRISMAScR): Checklist and Explanation. Ann Intern Med. 2018;169:467–473. doi: 10.7326/M18-0850.

S2 Table. Reasons for exclusion at full text review

First search, 27/05/2023	
**Citation, as extracted from Covidence**	**Summary reason for exclusion**
WHO recommendations on interventions to improve preterm birth outcomes. World Health Organisation Guidelines. 2015.	Study/guideline: wrong context (< 1 Nov 2015)
Correlation Between LIF (Leukemia Inhibitory Factor) Levels in Cord and Maternal Blood in Women Treated With Mg. Clinical Trials. 2015.	Study/guideline: wrong context (< 1 Nov 2015)
Bouet P-E, Brun S, Madar H, et al. Implementation of an antenatal magnesium sulfate protocol for fetal neuroprotection in preterm infants. Scientific Reports. 2015;5(1):1-8.	Study/guideline: wrong context (< 1 Nov 2015)
Brookfield K, Su F, Drover D, Adelus M, Lyell D, Carvalho B. 175: Pharmacokinetics of magnesium sulfate in pregnant women. American Journal of Obstetrics & Gynecology. 2015;212(1):S102.	Study/guideline: wrong context (< 1 Nov 2015)
De Jesus LC, Sood BG, Shankaran S, et al. Antenatal magnesium sulfate exposure and acute cardiorespiratory events in preterm infants. American Journal of Obstetrics and Gynecology. 2015;212(1):94. e91-94. e97.	Study/guideline: wrong context (< 1 Nov 2015)
Elizabeth A Marchant BKAASAvZTRKRBPML. Attenuated innate immune defenses in very premature neonates during the neonatal period. Pediatric Research. 2015:492-497.	Study/guideline: wrong context (< 1 Nov 2015)
Jocelyn C Leung CLCAGAC-CJSRMV. Antenatal factors modulate hearing screen failure risk in preterm infants. Archives of Disease in Childhood Fetal and Neonatal Edition. 2015:F56-61.	Study/guideline: wrong context (< 1 Nov 2015)
Kamyar M, Bardsley T, Korgenski K, Clark EAS. Association of antenatal magnesium sulfate with neonatal morbidity and mortality in very preterm infants. Reproductive Sciences. 2015;22:144A-144A.	Study/guideline: wrong context (< 1 Nov 2015)
Kim YH, Noh EJ, Kim JW, Song T-B. Total and Ionized Serum Magnesium, and Calcium Levels During Magnesium Sulfate Administration for Preterm labor. Reproductive Sciences. 2015;22:152A-152A.	Study/guideline: wrong context (< 1 Nov 2015)
Oddie S, Tuffnell DJ, McGuire W. Antenatal magnesium sulfate: neuro-protection for preterm infants. Archives of Disease in Childhood-Fetal and Neonatal Edition. 2015;100(6):F553-F557.	Study/guideline: wrong context (< 1 Nov 2015)
Paneth N. The causation and prevention of Cerebral Palsy (CP). Birth Defects Research Part A - Clinical and Molecular Teratology. 2015;103(5):365.	Study/guideline: wrong context (< 1 Nov 2015)
Soo-Young OH. Obstetrical Management of Periviable Birth. Korean Journal of Perinatology. 2015:1-11.	Study/guideline: wrong context (< 1 Nov 2015)
Stark MJ, Hodyl NA, Andersen CC. Effects of antenatal magnesium sulfate treatment for neonatal neuro-protection on cerebral oxygen kinetics. Pediatric Research. 2015;78(3):310-314.	Study/guideline: wrong context (< 1 Nov 2015)
Utz B, Zafar S, Arshad N, Kana T, Gopalakrishnan S, van den Broek N. Status of emergency obstetric care in four districts of Punjab, Pakistan-results of a baseline assessment. Journal of the Pakistan Medical Association. 2015;65(5):480-485.	Study/guideline: wrong context (< 1 Nov 2015)
Woods J, Gagliardi L, Nara S, et al. An innovative approach to in-service training of maternal health staff in Cambodian hospitals. International Journal of Gynaecology & Obstetrics. 2015;129(2):178-183.	Study/guideline: wrong context (< 1 Nov 2015)
Yadava S, Garabedian M, Sit A, El-Sayed Y. 390: Use of magnesium sulfate and labor outcomes in PPROM at less than 37 weeks. American Journal of Obstetrics & Gynecology. 2015;212(1):S203-S204.	Study/guideline: wrong context (< 1 Nov 2015)
Intrauterine growth restriction. Nordic Federation of Societies of Obstetrics and Gynecology. 2016.	Study/guideline: wrong context (not LMIC)
Management of Pregnancy. VA/DoD Clinical Practice Guidelines. 2018.	Study/guideline: wrong context (not LMIC)
Perinatal Management of Extreme Preterm Birth Before 27 weeks of Gestation. British Association of Perinatal Medicine. 2019.	Study/guideline: wrong context (not LMIC)
Preterm fetal monitoring. Nordic Federation of Societies of Obstetrics and Gynecology. 2019.	Study/guideline: wrong context (not LMIC)

Magnesium sulfate as neuroprotection in preterm birth. Nordic Federation of Societies of Obstetrics and Gynecology. 2020.	Study/guideline: wrong context (not LMIC)
Clinical Guideline: Maternity care for mothers and babies during the COVID-19 pandemic. Queensland Health. 2020.	Study/guideline: wrong context (not LMIC)
Preterm labour and birth. National Institute for Health and Clinical Excellence - Clinical Guidelines. 2022.	Study/guideline: wrong context (not LMIC)
The National COVID?19 Clinical Evidence Taskforce: pregnancy and perinatal guidelines. MJA Clinical Guidelines. 2022.	Study/guideline: wrong context (not LMIC)
Stephens AJ, Barton JR, Bentum NAA, Blackwell SC, Sibai BM. General Guidelines in the Management of an Obstetrical Patient on the Labor and Delivery Unit during the COVID-19 Pandemic. American Journal of Perinatology. 2020;37(8):829-836.	Study/guideline: wrong context (not LMIC)
The Effect of Magnesium on Maternal Mood, Cognitive Function, and Birth Experience. Clinical Trials. 2015.	Study/guideline: wrong context (not LMIC)
Alonso LG, Prieto MP, Colmenero EG, et al. Prenatal treatment with magnesium sulphate: Initial clinical outcomes in pre-term infants less than 29 weeks and correlation with neonatal magnesium levels. Anales de Pediatría (English Edition). 2017;86(3):135-141.	Study/guideline: wrong context (not LMIC)
Alonso LG, Prieto MP, Colmenero EG, et al. Prenatal therapy with magnesium sulfate and its correlation with neonatal serum magnesium concentration. American Journal of Perinatology. 2018;35(02):170-176.	Study/guideline: wrong context (not LMIC)
Arun J, Arasu A, Bhat R. ANTENATAL MAGNESIUM SULPHATE TREATMENT FOR FETAL NEUROPROTECTION: SHORT TERM AND LONG TERM OUTCOMES IN A TERTIARY NICU. Pediatric Research. 2019;86:51-51.	Study/guideline: wrong context (not LMIC)
Ayed M, Ahmed J, More K, et al. Antenatal Magnesium Sulfate for Preterm Neuroprotection: A Single-Center Experience from Kuwait Tertiary NICU. Biomedicine hub. 2022;7(2):80-87.	Study/guideline: wrong context (not LMIC)
Brookfield KF, Su F, Elkomy MH, Drover DR, Lyell DJ, Carvalho B. Pharmacokinetics and placental transfer of magnesium sulfate in pregnant women. American Journal of Obstetrics and Gynecology. 2016;214(6):737. e731-737. e739.	Study/guideline: wrong context (not LMIC)
Correia AL, Castro C, Morais JM, Portela A, Peixoto S. Hypermagnesemia in preterm neonates exposed to antenatal magnesium sulfate. Minerva Pediatrics. 2022.	Study/guideline: wrong context (not LMIC)
Deihl TE, Simhan HN. Antenatal Magnesium Sulfate and Ponderal Index From Birth to Age 2 in Preterm Male and Female Infants. Reproductive Sciences. 2017;24:130A-130A.	Study/guideline: wrong context (not LMIC)
Deihl TE, Simhan HN. Antenatal Magnesium Sulfate Exposure and Ponderal Index in Preterm Infants. American Journal of Perinatology. 2019;36(03):329-334.	Study/guideline: wrong context (not LMIC)
Díaz Vázquez M. Neuroprotection with antepartum magnesium sulfate in preterm birth between 32 and 34 weeks' gestation: multicentre randomized clinical trial. 2018.	Study/guideline: wrong context (not LMIC)
Doyle LW, Spittle AJ, Olsen JE, et al. Translating antenatal magnesium sulphate neuroprotection for infants born< 28 weeks' gestation into practice: A geographical cohort study. Australian and New Zealand Journal of Obstetrics and Gynaecology. 2021;61(4):513-518.	Study/guideline: wrong context (not LMIC)
Eswaran H, Escalona-Vargas DI, Thagard AS, Napolitano PG, Magann EF, Lowery CL. Observations of fetal brain activity via non-invasive magnetoencephalography following administration of magnesium sulfate for neuroprotection in preterm labor. Prenatal Diagnosis. 2016;36(10):982.	Study/guideline: wrong context (not LMIC)
Gano D, Ho M, Glass HC, Xu D, Barkovich AJ, Ferriero D. DECREASED RISK OF CEREBELLAR HEMORRHAGE IN PREMATURE NEWBORNS EXPOSED TO ANTENATAL MAGNESIUM SULFATE. Journal of Investigative Medicine. 2016;64(1):286-286.	Study/guideline: wrong context (not LMIC)
Gano D, Ho M-L, Partridge JC, et al. Antenatal exposure to magnesium sulfate is associated with reduced cerebellar hemorrhage in preterm newborns. The Journal of pediatrics. 2016;178:68-74.	Study/guideline: wrong context (not LMIC)

García Alonso L. 1, Pumarada Prieto M 2, et al., Prenatal treatment with magnesium sulphate: Initial clinical outcomes in pre-term infants less than 29 weeks and correlation with neonatal magnesium levels. An Pediatr (Barc). 2016:30184-30189.	Study/guideline: wrong context (not LMIC)
Gentle SJ, Carlo WA, Sylvia TAN, et al. Association of Antenatal Corticosteroids and Magnesium Sulfate Therapy With Neurodevelopmental Outcome in Extremely Preterm Infants. Obstetrics and Gynecology. 2020;135(6):1377.	Study/guideline: wrong context (not LMIC)
Georgiev V. Magnesium Sulphate Neuroprophylaxis in Pregnancy with Iugr Due to Hyperitensive Disease. 2018(10972960):55.	Study/guideline: wrong context (not LMIC)
Gray KB, Sorensen AV, Sommerness SA, et al. Implementation experiences with improving safe medication practices for oxytocin and magnesium sulfate during labor and delivery. Research on Women's Health (RWH). 2018.	Study/guideline: wrong context (not LMIC)
Hong JAX, Mathur M. Resident Quality Improvement Project: Antenatal Magnesium Sulfate Protocol for Fetal Neuroprotection in Preterm Births. Obstet Gynecol Int J. 2017;7(5):00265.	Study/guideline: wrong context (not LMIC)
Hong JY, Hong JY, Choi Y-S, et al. Antenatal magnesium sulfate treatment and risk of necrotizing enterocolitis in preterm infants born at less than 32 weeks of gestation. Scientific Reports. 2020;10(1):12826.	Study/guideline: wrong context (not LMIC)
Hong JY, Kim Y-M, Hong JY, et al. 489: Does antenatal magnesium sulfate exposure increase the risk of necrotizing enterocolitis in preterm neonates? American Journal of Obstetrics & Gynecology. 2019;220(1):S327.	Study/guideline: wrong context (not LMIC)
Hurrion EM, Colditz PB, Boyd RN, et al. G490 (P) Neuroprotective benefit of antenatal magnesium sulfate for preterm infants. Is it the magnesium or the sulfate? : BMJ Publishing Group Ltd; 2017.	Study/guideline: wrong context (not LMIC)
Inomata S, Yoshida T, Nagaoka M, et al. Effects of long-term antenatal magnesium sulfate administration on the bone mineralization of preterm infants. Journal of Obstetrics and Gynaecology Research. 2022.	Study/guideline: wrong context (not LMIC)
Isrctn, Imperial College London Y. When to deliver small babies between 32 and 37 weeks. 2020.	Study/guideline: wrong context (not LMIC)
Jeong EJ, Park YH, Cho MH, et al. Antenatal magnesium sulfate for both short-term tocolysis and fetal neuroprotection in premature rupture of the membranes remote from term. 대한산부인과학회 학술발표논문집. 2016;102:415-415.	Study/guideline: wrong context (not LMIC)
Jones CW, Petrashek K, Wenzlaff M, Simpson P, Pan AY. Prenatal Magnesium Sulfate and Time to First Stool in Late Preterm Infants [32N]. Obstetrics & Gynecology. 2018;131:160S.	Study/guideline: wrong context (not LMIC)
Jung EJ, Byun JM, Kim YN, et al. Antenatal magnesium sulfate for both tocolysis and fetal neuroprotection in premature rupture of the membranes before 32 weeks' gestation. The Journal of Maternal-Fetal & Neonatal Medicine. 2018;31(11):1431-1441.	Study/guideline: wrong context (not LMIC)
Kamyar M, Clark EAS, Yoder BA, Varner MW, Manuck TA. Antenatal magnesium sulfate, necrotizing enterocolitis, and death among neonates< 28 weeks gestation. American Journal of Perinatology Reports. 2016;6(01):e148-e154.	Study/guideline: wrong context (not LMIC)
Kim SH, Kim Y-J, Shin SH, et al. Antenatal Magnesium Sulfate Reduced Intestinal Morbidities Requiring Surgery in Preterm Infants With Extremely Low Gestational Age: A Retrospective Cohort Study. 2020.	Study/guideline: wrong context (not LMIC)
Kim SH, Kim Y-J, Shin SH, et al. Antenatal magnesium sulfate and intestinal morbidities in preterm infants with extremely low gestational age. Pediatrics & Neonatology. 2021;62(2):202-207.	Study/guideline: wrong context (not LMIC)
Kim WH, Kim YH. Yuna An, Jong Ho Moon, Eun Ji Noh, Jong Woon Kim. Total and ionized serum magnesium and calcium levels during magnesium sulfate administration for preterm labor. Obstet Gynecol Sci 2018; 61 (1): 56-62 https://doi org/105468/ogs. 2018;1.	Study/guideline: wrong context (not LMIC)
Kotidis C, Sharp A, Alfirevic Z, Weindling M, Turner M. NEONATAL CARDIOVASCULAR AND CEREBRAL FUNCTION AFTER ANTENATAL MATERNAL EXPOSURE TO MAGNESIUM SULFATE. European Journal of Pediatrics. 2016;175(11):1484-1484.	Study/guideline: wrong context (not LMIC)
Kotidis C, Turner M, Weindling A, Subhedar N. Haemodynamics in Preterm Infants with Patent Ductus Arteriosus. 2022(29920232):437.	Study/guideline: wrong context (not LMIC)

Lloreda-Garcia JM, Lorente-Nicolás A, Bermejo-Costa F, Martínez-Uriarte J, López-Pérez R. Need for resuscitation in preterm neonates less than 32 weeks treated with antenatal magnesium sulphate for neuroprotection. Revista Chilena de Pediatria. 2016;87(4):261-267.	Study/guideline: wrong context (not LMIC)
Mei JY, Lee D, Negi M. Duration of labor induction in nulliparous women receiving magnesium sulfate and maternal and neonatal outcomes. American Journal of Obstetrics & Gynecology. 2023;228(1):S662.	Study/guideline: wrong context (not LMIC)
Mena N P, León del P J, Sandino P D, et al. Evacuación del meconio intestinal para mejorar tolerancia alimentaria en prematuro de muy bajo peso (protocolo Emita). Rev Soc Boliv Pediatr. 2016;55(1):57-64.	Study/guideline: wrong context (not LMIC)
Mikhael M, Bronson C, Zhang L, Curran M, Rodriguez H, Bhakta KY. Lack of evidence for time or dose relationship between antenatal magnesium sulfate and intestinal injury in extremely preterm neonates. Neonatology. 2019;115(4):371-378.	Study/guideline: wrong context (not LMIC)
Mota R, Rey y Formoso V, Soares P, Guimaraes H. Antenatal Magnesium Sulfate: Retrospective Study of Clinically Relevant Outcomes in Preterm Neonates. European Journal of Pediatrics. 2019;178(11):1744-1744.	Study/guideline: wrong context (not LMIC)
Nct, Yes CHUdR. Magnesium Sulfate (MgSO4) and Fetal Heart Rate (FHR) in Case of Prematurity. 2018.	Study/guideline: wrong context (not LMIC)
O'Brien JM, Santolaya JL, Palomares K, Blitzer D, Santolaya-Forgas J. Association of histological chorioamnionitis and magnesium sulfate treatment in singleton and dichorionic twin pregnancies with preterm premature rupture of membranes: preliminary observations. Journal of Perinatal Medicine. 2018;46(8):839-844.	Study/guideline: wrong context (not LMIC)
Okito O, Aromolaran A, Massa-Buck B, Abdelatif D, Aly H, Mohamed MA. Antenatal Magnesium Sulfate and the Need for Mechanical Ventilation in the First Three Days of Life. Pediatrics & Neonatology. 2023.	Study/guideline: wrong context (not LMIC)
Pinto MP, Costa RJB, Dias Nunes SMM. Seguimento de Recém-nascidos pré-termo Tardios no CHUCB. 2021(29009752):69.	Study/guideline: wrong context (not LMIC)
Qasim A, Jain S, Dasgupta S. Does antenatal magnesium sulfate increase the likelihood of a hemodynamically significant patent ductus arteriosus in neonates? Journal of Investigative Medicine. 2017;65(2):547-548.	Study/guideline: wrong context (not LMIC)
Qasim A, Jain SK, Aly AM. Antenatal magnesium sulfate exposure and hemodynamically significant patent ductus arteriosus in premature infants. American Journal of Perinatology Reports. 2019;9(04):e353-e356.	Study/guideline: wrong context (not LMIC)
Raouf S. VP52. 22: The uptake of magnesium sulphate (MgSO4) administration for neuroprotection of preterm birth: an East Midlands tertiary joint obstetric and neonatal experience. Ultrasound in Obstetrics & Gynecology. 2020;56:300-300.	Study/guideline: wrong context (not LMIC)
Revenga JT, Peña-Moreno A, Arriaga-Redondo M, et al. Antenatal magnesium sulphate and delayed passage of meconium: A multicentre study. Anales de Pediatría (English Edition). 2022;97(6):383-389.	Study/guideline: wrong context (not LMIC)
Reynolds A, Slattery S, Byrne S, et al. Timing of administration of antenatal magnesium sulfate and umbilical cord blood magnesium levels in preterm babies. The Journal of Maternal-Fetal & Neonatal Medicine. 2019;32(6):1014-1019.	Study/guideline: wrong context (not LMIC)
Richter AE, Scherjon SA, Dikkers R, Bos AF, Kooi EMW. Antenatal magnesium sulfate and preeclampsia differentially affect neonatal cerebral oxygenation. Neonatology. 2020;117(3):331-340.	Study/guideline: wrong context (not LMIC)
Saldanha J, Moniz C, Machado MDC. Very low birth weight infants in a Portuguese intensive care unit and the Vermont Oxford network: 15 years of registry data. [Portuguese]. Acta Medica Portuguesa. 2019;32(11):686-692.	Study/guideline: wrong context (not LMIC)
Shalabi M, Mohamed A, Lemyre B, et al. Antenatal exposure to magnesium sulfate and spontaneous intestinal perforation and necrotizing enterocolitis in extremely preterm neonates. American Journal of Perinatology. 2017;34(12):1227-1233.	Study/guideline: wrong context (not LMIC)
Shaw A, Talbot H. G117 (P) Evaluation of the use of antenatal magnesium sulphate prior to in-utero transfer. BMJ Publishing Group Ltd; 2018.	Study/guideline: wrong context (not LMIC)
Sung SI, Ahn SY, Choi S-J, et al. Increased risk of meconium-related ileus in extremely premature infants exposed to antenatal magnesium sulfate. Neonatology. 2022;119(1):68-76.	Study/guideline: wrong context (not LMIC)

Valdovinos AG, Arriaga-Redondo M, Bitriá ED, Rodríguez IP, Isidro EM, Bravo DB. Prenatal therapy with magnesium sulphate and intestinal obstruction due to meconium in preterm newborns. Anales de Pediatría (English Edition). 2022;96(2):138-144.	Study/guideline: wrong context (not LMIC)
Vanhaesebrouck S, Zecic A, Goossens L, et al. Association of antenatal magnesium sulfate with reduced late-onset sepsis in extreme preterm infants. Acta Clinica Belgica. 2023;78(1):11-15.	Study/guideline: wrong context (not LMIC)
Young S, Wang MJ, Srivastava A, et al. Intrapartum magnesium sulfate exposure and obstetric hemorrhage risk. The Journal of Maternal-Fetal & Neonatal Medicine. 2022;35(25):10036-10043.	Study/guideline: wrong context (not LMIC)
Yun-Sun C, Ji-Young H, Jee-Youn H, et al. The effects of maternal body mass index and plurality on maternal and umbilical cord serum magnesium levels in preterm birth at less than 32 weeks of gestation. Obstetrics & Gynecology Science. 2021:62-72.	Study/guideline: wrong context (not LMIC)
Preterm Labor. Medscape. 2020.	Study/guideline: wrong design
Prematurity. Medscape Pediatrics. 2020.	Study/guideline: wrong design
Premature Rupture of Membranes. Medscape. 2020.	Study/guideline: wrong design
Abu-Faza M, Abdelazim IA. Antenatal magnesium sulphate (MgSO4) for fetal neuroprotection prior to preterm labor: Mini-review. ARC J Gynecol Obstet. 2017;2:11-14.	Study/guideline: wrong design
CRACKCast E180 – Labor & Delivery. CandiEM. 2018.	Study/guideline: wrong design
Pearson S, Loubser L, Nguyen K, Curry A. Should magnesium sulfate be given for neuroprotection for preterm labor between 32 and 34 weeks' gestation? Evidence-Based Practice.10.1097.	Study/guideline: wrong design
Magnesium Sulfate. FP Notebook. 2018.	Study/guideline: wrong design
Cerebral palsy. BMJ Best Practice. 2019.	Study/guideline: wrong design
Maternal and Newborn Survival in Sub-Saharan Africa. Academy of Medical Sciences. 2019.	Study/guideline: wrong design
The effect of antenatal magnesium sulfate on intraventricular hemorrhage in premature infants: a systematic review and meta-analysis. Obstetrics & Gynecology Science. 2020.	Study/guideline: wrong design
Fetal Growth Restriction. Medscape. 2020.	Study/guideline: wrong design
Quality of medicines for life-threatening pregnancy complications in low- and middle-income countries: A systematic review. PLoS ONE [Electronic Resource]. 2020.	Study/guideline: wrong design
Hypermagnesemia. Medscape. 2020.	Study/guideline: wrong design
Pediatric Hypermagnesemia. Medscape Pediatrics. 2020.	Study/guideline: wrong design
Intestinal Obstruction in the Newborn. Medscape Pediatrics. 2020.	Study/guideline: wrong design
Antenatal magnesium sulfate and gastrointestinal complications in preterm infants - A systematic review and meta-analysis. PROSPERO. 2022.	Study/guideline: wrong design
Antenatal magnesium and chorioamnionitis: a systematic review. PROSPERO. 2023.	Study/guideline: wrong design
Bell V, Fitzgerald D, Twomey A, Carroll S, Murphy JF. Antenatal Magnesium Sulphate: Preventing Cerebral Palsy in Preterm Infants.	Study/guideline: wrong design
Binder C, Schmid P, Abele H, Graf J. Does Antenatal MgSO4 Administration to the Mother in the Event of Imminent Premature Birth Reduce the Occurrence of Infantile Cerebral Palsy in the Child?–An Umbrella Review. Geburtshilfe und Frauenheilkunde. 2023;83(05):602-611.	Study/guideline: wrong design

Brookfield KF, Mbata O. Magnesium Sulfate Use in Pregnancy for Preeclampsia Prophylaxis and Fetal Neuroprotection: Regimens in High-Income and Low/Middle-Income Countries. Obstetrics & Gynecology Clinics of North America. 2023;50(1):89-99.	Study/guideline: wrong design
Cason I, Rocha CA, Goldman RE. Preterm premature rupture of membranes: management between 28 and 34 weeks of pregnancy. ABCS health sci. 2021;46:e021309-e021309.	Study/guideline: wrong design
Chollat C, Sentilhes L, Marret S. Protection of brain development by antenatal magnesium sulphate for infants born preterm. Developmental Medicine & Child Neurology. 2019;61(1):25-30.	Study/guideline: wrong design
Coutinho T, Coutinho CM, Coutinho LM. Neuroproteção fetal: uma utilização contemporânea do sulfato de magnésio. Femina. 2019;47(2):114-121.	Study/guideline: wrong design
Coutinho T, Coutinho CM, Coutinho LM. Sulfato de magnésio: principais utilizações na obstetrícia contemporânea. Rev méd Minas Gerais. 2021;31:30211-30211.	Study/guideline: wrong design
Cruz CNR, Santana FZ, Silva GASe, Carvalho GBd, Paula CCd, Casanova MdS. Rotura prematura de membrana: abordagem clínica. Femina. 2018;46(1):48-53.	Study/guideline: wrong design
Editors of eBioMedicine T. Retraction and republication-effect of antenatal magnesium sulphate on MRI biomarkers of white matter development at term equivalent age: The MagNUM Study. EBioMedicine. 2022;78.	Study/guideline: wrong design
Elito Jr J, Shuai MG. Antenatal Corticosteroids and Magnesium Sulfate in Twin Pregnancy for the Prevention of Neonatal Morbidity. Topics on Critical Issues in Neonatal Care. 2022:1.	Study/guideline: wrong design
Emily Shepherd RASDMASPMMMCAC. Antenatal magnesium sulphate and adverse neonatal outcomes: A systematic review and meta-analysis. EvidenceUpdates. 2019:e1002988.	Study/guideline: wrong design
Garg B, Dash SK. Neuroprotective Role of Antenatal Magnesium Sulphate in Preterms. NEOCHAP BULLETIN. 2016;186:10.	Study/guideline: wrong design
Garg BD. Antenatal magnesium sulfate is beneficial or harmful in very preterm and extremely preterm neonates: a new insight. The Journal of Maternal-Fetal & Neonatal Medicine. 2019;32(12):2084-2090.	Study/guideline: wrong design
Goldenberg RL, McClure EM. Improving birth outcomes in low-and middle-income countries. New England Journal of Medicine. 2017;377(24):2387-2388.	Study/guideline: wrong design
Hong JY, Hong JY, Choi Y-S, et al. Author Correction: Antenatal magnesium sulfate treatment and risk of necrotizing enterocolitis in preterm infants born at less than 32 weeks of gestation. Scientific Reports. 2020;10(1):20209.	Study/guideline: wrong design
Huusom LD, Wolf HT. Antenatal magnesium sulfate treatment for women at risk of preterm birth is safe and might decrease the risk of cerebral palsy. BMJ Evidence-Based Medicine. 2018;23(5):195-196.	Study/guideline: wrong design
Jayaram PM, Mohan MK, Farid I, Lindow S. review of clinical practice guidelines. Journal Antenatal magnesium sulfate for fetal neuroprotection: a critical appraisal and systematic of Perinatal Medicine. 2019;47(3):262-269.	Study/guideline: wrong design
Kobayashi A, Ito M, Kato A, Namba F, Ota E. SCHOOL-AGE OUTCOMES OF PRETERM INFANTS WHO RECEIVED ANTENATAL MAGNESIUM SULPHATE THERAPY: A SYSTEMATIC REVIEW AND META-ANALYSIS. Pediatric Research. 2019;86:61-62.	Study/guideline: wrong design
Larsen ML, Krebs L, Rackauskaite G, Hoei-Hansen CE, Greisen G. Re: Antenatal magnesium sulphate for the prevention of cerebral palsy in infants born preterm: a double-blind, randomised, placebo-controlled, multi-centre trial: At which gestational ages should magnesium sulphate be given to women at risk of preterm birth? BJOG: An International Journal of Obstetrics and Gynaecology. 2020;127(10):1295-1296.	Study/guideline: wrong design
Lesley M McCowan FFNHA. Evidence-based national guidelines for the management of suspected fetal growth restriction: comparison, consensus, and controversy. American Journal of Obstetrics and Gynecology. 2018:S855-S868.	Study/guideline: wrong design
Marret S, Ancel PY. Neuroprotection for preterm infants with antenatal magnesium sulphate. Journal de gynecologie, obstetrique et biologie de la reproduction. 2016;45(10):1418-1433.	Study/guideline: wrong design

Pejaver RK. Low cost interventions to improve outcomes in newborns. Journal of Perinatal Medicine. 2017;45(Supplement 2):148.	Study/guideline: wrong design
Prasath A, Aronoff N, Chandrasekharan P, Diggikar S. Antenatal Magnesium Sulfate and Adverse Gastrointestinal Outcomes in Preterm Infants-A Systematic Review and Meta-Analysis. Authorea Preprints. 2023.	Study/guideline: wrong design
Ran D, Juan Q, Hongbo QI. Diagnosis and management of fetal growth restriction: from experience to evidence-based practice. Chinese Journal of Perinatal Medicine. 2019(12):381-384.	Study/guideline: wrong design
Rana Islamiah Zahroh AHKEEJPVÖTNMFAOTOMAB. Factors influencing appropriate use of interventions for management of women experiencing preterm birth: A mixed-methods systematic review and narrative synthesis. PLoS Medicine. 2022:e1004074.	Study/guideline: wrong design
Rodríguez-Hernández PA, Beltrán-Avendaño MA. Aproximación a la farmacología del sulfato de magnesio desde la perspectiva obstétrica. MedUNAB. 2016;19(1):25-32.	Study/guideline: wrong design
Seligman KM, Abir G. Emergency Resources in Obstetrics. Anesthesiology Clinics. 2021;39(4):631-647.	Study/guideline: wrong design
Si-Meng WEI. Recent research on the effect of common treatments given in the perinatal period on neurodevelopment in offspring. Chinese Journal of Contemporary Pediatrics. 2022(12):332-338.	Study/guideline: wrong design
Sonia Giouleka ITNKGKIKAMAATD. Preterm Labor: A Comprehensive Review of Guidelines on Diagnosis, Management, Prediction and Prevention. Obstetrical & Gynecological Survey. 2022:302-317.	Study/guideline: wrong design
Ting JY, Kingdom JC, Shah PS. Antenatal glucocorticoids, magnesium sulfate, and mode of birth in preterm fetal small for gestational age. American Journal of Obstetrics and Gynecology. 2018;218(2):S818-S828.	Study/guideline: wrong design
Tsakiridis I, Mamopoulos A, Athanasiadis A, Dagklis T. Antenatal corticosteroids and magnesium sulfate for improved preterm neonatal outcomes: a review of guidelines. Obstetrical & Gynecological Survey. 2020;75(5):298-307.	Study/guideline: wrong design
Wenwen HE, Mingyan HEI. Research progress on risk factors of spontaneous intestinal perforation in premature infants. Chinese Pediatric Emergency Medicine. 2022(12):75-77.	Study/guideline: wrong design
Magnesium Sulfate Dose in Obese Patients. Clinical Trials. 2019.	Study: wrong concept/intervention ($MgSO_4$ for other indication)
Assiut U. Magnesium Sulphate in Premature Rupture of Membranes. https://ClinicalTrials.gov/show/NCT05134688; 2022.	Study: wrong concept/intervention ($MgSO_4$ for other indication)
Magnesium Therapy in Children With Cerebral Palsy. Clinical Trials. 2015.	Study: wrong concept/intervention ($MgSO_4$ for other indication)
Ctri, Pt BDshuY. Magnesium suplphate in protection against brain injury in newborn. 2018.	Study: wrong concept/intervention ($MgSO_4$ for other indication)
Huang HB, Watt MJ, Hicks M, et al. A Family-Centered, Multidisciplinary Clinic for Early Diagnosis of Neurodevelopmental Impairment and Cerebral Palsy in China-A Pilot Observation. Frontiers in Pediatrics. 2022;10 (no pagination).	Study: wrong concept/intervention ($MgSO_4$ for other indication)
Mekonnen SM, Bekele DM, Fenta FA, Wake AD. The Prevalence of Necrotizing Enterocolitis and Associated Factors Among Enteral Fed Preterm and Low Birth Weight Neonates Admitted in Selected Public Hospitals in Addis Ababa, Ethiopia: A Cross-sectional Study. Global Pediatric Health. 2021;8(no pagination).	Study: wrong concept/intervention ($MgSO_4$ for other indication)
Nct, Cairo University Y. Magnesium Sulfate for Fetal Neuroprotection. 2020.	Study: wrong concept/intervention

	(MgSO4 for other indication)
Electricity Access and Maternal Care in Rural Health Facilities in Uganda. Clinical Trials. 2018.	Study: wrong concept/intervention (MgSO4 for other indication)
Ambadkar A, Prasad M, Chauhan AR. Neonatal effects of maternal magnesium sulphate in late preterm and term pregnancies. The Journal of Obstetrics and Gynecology of India. 2019;69:25-30.	Study: wrong concept/intervention (MgSO4 for other indication)
Amoakoh-Coleman M, Agyepong IA, Kayode GA, Grobbee DE, Klipstein-Grobusch K, Ansah EK. Public health facility resource availability and provider adherence to first antenatal guidelines in a low resource setting in Accra, Ghana. BMC Health Services Research. 2016;16(1):505.	Study: wrong concept/intervention (MgSO4 for other indication)
Bozkurt O, Eras Z, Canpolat FE, Oguz SS, Uras N, Dilmen U. Antenatal magnesium sulfate and neurodevelopmental outcome of preterm infants born to preeclamptic mothers. The Journal of Maternal-Fetal & Neonatal Medicine. 2016;29(7):1101-1104.	Study: wrong concept/intervention (MgSO4 for other indication)
Castillo MS, Corsino MA, Calibo AP, et al. Turning Disaster into an Opportunity for Quality Improvement in Essential Intrapartum and Newborn Care Services in the Philippines: Pre- to Posttraining Assessments. BioMed Research International. 2016;2016:6264249.	Study: wrong concept/intervention (MgSO4 for other indication)
de Oliveira-Filho AD, Vieira AES, da Silva RC, et al. Adverse drug reactions in high-risk pregnant women: A prospective study. Saudi Pharmaceutical Journal. 2017;25(7):1073-1077.	Study: wrong concept/intervention (MgSO4 for other indication)
Hassan L, Musa S, Abdullahi FL, Sarkin-pawa Z, Abdulkadir I. Maternal antepartum Use of Magnesium Sulfate and immediate Neonatal Outcomes at the Ahmadu Bello University Teaching Hospital Shika, Zaria.	Study: wrong concept/intervention (MgSO4 for other indication)
Kananura RM, Kiwanuka SN, Ekirapa-Kiracho E, Waiswa P. Persisting demand and supply gap for maternal and newborn care in eastern Uganda: a mixed-method cross-sectional study. Reproductive Health. 2017;14(1):136.	Study: wrong concept/intervention (MgSO4 for other indication)
Lama TP, Munos MK, Katz J, Khatry SK, LeClerq SC, Mullany LC. Assessment of facility and health worker readiness to provide quality antenatal, intrapartum and postpartum care in rural Southern Nepal. BMC Health Services Research. 2020;20(1):16.	Study: wrong concept/intervention (MgSO4 for other indication)
Madeiro A, Rufino AC, Nunes MdDS, et al. Analysis of the structural adequacy of maternity hospitals in Piauí, Brazil, 2018-2019. Rev Bras Saúde Mater Infant (Online). 2022;22(2):267-273.	Study: wrong concept/intervention (MgSO4 for other indication)
Phd TA, Phd TD, Chinesho A. ADVERSE BIRTH OUTCOME AND ASSOCIATED FACTORS AMONG PREGNANT WOMEN MANAGED BY MAGNESIUM SULPHATE IN HIWOT FANA COMPREHENSIVE SPECIALIZED UNIVERSITY HOSPITAL, HARARI REGIONAL STATE, EASTERN ETHIOPIA. 2021.	Study: wrong concept/intervention (MgSO4 for other indication)
Chowdhary S, Khajuria R, Jaggi R. Role of magnesium Sulfate in Preterm labour-A prospective study. Annals of the Romanian Society for Cell Biology. 2021;25(7):1818-1832.	Study: wrong concept/intervention (MgSO4 for other indication)
Dolly M, Mammen MV. Retrospective Study of Neonatal Outcomes with the Use of Magnesium Sulfate in Case of Preterm Labor. Journal homepage: www ijrpr com ISSN.2582:7421.	Study: wrong concept/intervention (MgSO4 for other indication)
Essam Rady Hashish M, Mohamed Talaat El-Garhy I, Fawzy El-Sharkawy M. EFFECT OF MAGNESIUM SULFATE ON DOPPLER INDICES OF UTERINE, UMBILICAL AND FETAL MIDDLE CEREBRAL ARTERIES IN	Study: wrong concept/intervention (MgSO4 for other indication)

WOMEN WITH THREATENED PRETERM LABOUR. Al-Azhar Medical Journal. 2022;51(4):2077-2090.	
Hua LIU, Haijian MA, Hongchao G. Risk warning management in pregnant women with late threatened abortion by magnesium sulfate treatment. 实用临床医药杂志. 2020;24(17):118-121.	Study: wrong concept/intervention (MgSO4 for other indication)
Karkour TA, Abdel-Fattah EA, Ibrahim AELA, Mohamed T, Karawya ASM. MAGNESIUM SULFATE EFFECT ON FETAL CEREBROPLACENTAL DOPPLER INDICES IN CASES OF THREATENED PRETERM LABOUR. ALEXMED ePosters. 2023;5(2):13-14.	Study: wrong concept/intervention (MgSO4 for other indication)
Nct, Assiut University Y. Magnesium Sulphate in Premature Rupture of Membranes. 2021.	Study: wrong concept/intervention (MgSO4 for other indication)
Padmavathi K, Shobha B. Role of Magnesium Sulphate In Management of Preterm Labor.	Study: wrong concept/intervention (MgSO4 for other indication)
Senadhipathi S, Ch A. Role of magnesium sulphate in patients with pregnancy. 2018.	Study: wrong concept/intervention (MgSO4 for other indication)
Buckley A, Persad MD, Milone GF, Pardo C, Davilmar I, Garretto DJ. An Evaluation of the Maternal and Neonatal Characteristics Associated with Preterm Birth in Haiti. Obstetrics and Gynecology Conference: 67th Annual Clinical and Scientific Meeting of the American College of Obstetricians and Gynecologists Nashville, TN United States. 2019;133(SUPPL 1).	Study: wrong concept/intervention (cannot determine MgSO4 indication)
Peng H, Shi Y, Wang F, et al. Comparisons of care practices for very preterm infants and their short-term outcomes in two tertiary centers in northwest and south China: A retrospective cohort study. BMC Pediatrics. 2022;22(1) (no pagination).	Study: wrong concept/intervention (cannot determine MgSO4 indication)
Rodrigo FGM, Fabres JG, Nieto CZ, et al. Survival and Survival without Major Morbidity Seem to Be Consistently Better throughout Gestational Age in 24- to 30-Week Gestational Age Very-Low-Birth-Weight Female Infants Compared to Males. Neonatology. 2022;119(5):585-593.	Study: wrong concept/intervention (cannot determine MgSO4 indication)
Wang Y, Liu L, Huang Y. Effect of antenatal dexamethasone therapy on hearing screening in premature infants: A retrospective case-control study. American Journal of Otolaryngology - Head and Neck Medicine and Surgery. 2023;44(2) (no pagination).	Study: wrong concept/intervention (cannot determine MgSO4 indication)
Xi-Shi Lin X-YPM-MYL-LNY-WSYJS-WFQL. The single pregnancy predicting model of 1 minute Apgar score less than 7 after preterm birth: A retrospective study. PLoS ONE [Electronic Resource]. 2022:e0279385.	Study: wrong concept/intervention (cannot determine MgSO4 indication)
Xinxin DU, Guangxia LEI, Jinling Z, Chunyan L, Xiaoxia C, Jian W. Risk factors of periventricular-intraventricular hemorrhage in premature infants. Chinese Pediatric Emergency Medicine. 2022(12):665-670.	Study: wrong concept/intervention (cannot determine MgSO4 indication)
Protecting Brains and Saving Futures - the PBSF Protocol. Clinical Trials. 2019.	Study: wrong concept/intervention (not MgSO4)
Sahu M, Panda R, Das S. Factors leading to early preterm premature rupture of membranes in a tertiary care centre in eastern india: A prospective study. Journal of Clinical and Diagnostic Research. 2020;14(6):QC01-QC05.	Study: wrong concept/intervention (not MgSO4)
Yan C, Deng X, Hong F. Analysis of Maternal and Neonatal Outcome of Patients with Preterm Prelabor Rupture of Membranes. Journal of Healthcare Engineering. 2022;2022 (no pagination).	Study: wrong concept/intervention (not MgSO4)
Bulbul M, Bucak IH. MgSO4 prophylaxis for obstetric reasons: Traditional solutions vs ready-made solutions. 2019.	Study: wrong population

Akila C, Rozati R. Role of Magnesium Sulphate in Patients with Pregnancy. 2016(28507530):128.	Study: cannot access
Aoki K, Matsuuchi S, Akaba K. Effects of antenatal magnesium sulfate on early neonatal hyperkalemia in preterm infants. J Jpn Soc Perin Neon Med. 2018;54(1037):e42.	Study: cannot access
Chung SH, Im HS, Lee JE, et al. OB-10: Effects of Antenatal Magnesium Sulfate Exposure on Oxygen Support in Preterm Neonates. 대한산부인과학회 학술발표논문집. 2015;101:259-259.	Study: cannot access
Hong JY, Sung J-h, Oh S-y. OB03: Routine use or non-use of antenatal magnesium sulfate exposure for neuroprotection and risk of necrotizing enterocolitis in preterm neonates. 대한산부인과학회 학술발표논문집. 2018;104:202-202.	Study: cannot access
Mettänen R. Implementation of antenatal magnesium sulfate for fetal neuroprotection in the third-level teaching university hospital: The retrospective analysis in period 2012-2016. 2017.	Study: cannot access
Pandey R, Arnold C, Duncan A. EXTREMELY LOW BIRTH WEIGHT INFANTS WITH ANTENATAL EXPOSURE TO MAGNESIUM SULFATE ARE LESS LIKELY TO HAVE CEREBRAL PALSY. Journal of Investigative Medicine. 2019;67(2):526-526.	Study: cannot access
Park J-A. Does Antenatal Infusion of Magnesium Sulphate in Preterm and Moderate Preterm Labour (up to 33+ 6 Weeks of Gestation) Improve Neurodevelopmental Outcomes:(with Associated Recommendations for Practice). 2017.	Study: cannot access
Ashouri N, Kordi M, Shakeri MT, Tara F. Vaginal delivery postpartum hemorrhage: Incidence, risk factors, and causes. Iranian Journal of Obstetrics, Gynecology and Infertility. 2019;21(12):65-76.	Study: translation not available
**Top up search, 13/1/2025**	
Hellström S, Jonsdotter A, Jonsson M, et al. A follow up on the feasibility after national implementation of magnesium sulfate for neuroprotection prior to preterm birth. Acta Obstetricia et Gynecologica Scandinavica. 2023;102(12):1741-1748.	Study/guideline: wrong context (not LMIC)
Inoue M, Sekiguchi K, Tsushita H, et al. Metabolomic characteristics of cord blood from neonates with hyperkalemia after antenatal exposure to ritodrine and magnesium sulfate. 2024.	Study/guideline: wrong context (not LMIC)
Jo Y, Kim Y, Ko H, et al. EP20. 03: Risk factors and prediction model for high neonatal serum magnesium concentrations in prenatal maternal magnesium sulfate therapy. Ultrasound in Obstetrics & Gynecology. 2023;62:229-230.	Study/guideline: wrong context (not LMIC)
McLeod RM, Rosenkrantz TS, Fitch RH. Antenatal Magnesium Sulfate Benefits Female Preterm Infants but Results in Poor Male Outcomes. Pharmaceuticals. 2024;17(2):218.	Study/guideline: wrong context (not LMIC)
Monteagudo BF, Castro SV, García PC, Sarrato SZ, Luna MS. Neuroprotective effect of magnesium sulfate in premature infants. Analysis after establishing an antenatal administration protocol in a tertiary care hospital. Anales de Pediatría (English Edition). 2023;99(4):224-231.	Study/guideline: wrong context (not LMIC)
Nagayasu Y, Mitsuhashi R, Fujita D, Ohmichi M. EP01. 33: Examination of fetal breathing movements following magnesium sulphate treatment to the mother using artificial intelligence. Ultrasound in Obstetrics & Gynecology. 2024;64:116-116.	Study/guideline: wrong context (not LMIC)
Okito O, Aromolaran A, Massa-Buck B, Abdelatif D, Aly H, Mohamed MA. Antenatal magnesium sulfate and the need for mechanical ventilation in the first three days of life. Pediatrics & Neonatology. 2023;64(6):644-650.	Study/guideline: wrong context (not LMIC)
Omori-Shimano S, Tominaga T, Ikeda K. Maternal magnesium sulfate administration increases early-onset hyperkalemia risk in premature infants: A propensity score-matched, case-control study. Pediatrics & Neonatology. 2023;64(2):119-125.	Study/guideline: wrong context (not LMIC)
Vanhaesebrouck S, Zecic A, Goossens L, et al. Association of antenatal magnesium sulfate with reduced late-onset sepsis in extreme preterm infants. Acta Clinica Belgica. 2023;78(1):11-15.	Study/guideline: wrong context (not LMIC)

猪又智実. Effects of long-term antenatal magnesium sulfate administration on the bone mineralization of preterm infants. 2023.	Study/guideline: wrong context (not LMIC)
Hall M, Valencia CM, Soma-Pillay P, Luyt K, Jacobsson B, Shennan A. Effective and simple interventions to improve outcomes for preterm infants worldwide: The FIGO PremPrep-5 initiative. International Journal of Gynaecology & Obstetrics. 2024;165(3):929-935.	Study/guideline: wrong design
Jeon GW. Long-Term Neuroprotective Effect and Safety of Antenatal Magnesium Sulfate on Preterm Infants. Perinatology. 2023;34(3):105-113.	Study/guideline: wrong design
Mengtong LIU, Huixia Y. Progress in antenatal magnesium sulfate exposure for neuroprotection in preterm infants. Chinese Journal of Perinatal Medicine. 2023(12):68-71.	Study/guideline: wrong design
Pearson S, Loubser L, Nguyen K, Curry A. Should magnesium sulfate be given for neuroprotection for preterm labor between 32 and 34 weeks' gestation? Evidence-Based Practice. 2023;26(4):26-27.	Study/guideline: wrong design
Iftikhar S, Riaz M, Rafique S. Efficacy of antenatal magnesium sulfate in the prevention of necrotizing enterocolitis: A randomized case-control study in preterm neonates. The Professional Medical Journal. 2024;31(07):1018-1022.	Study: wrong concept/intervention (MgSO4 for other indication)
Karkour TA, Abdel-Fattah EA, Ibrahim AELA, Mohamed T, Karawya ASM. MAGNESIUM SULFATE EFFECT ON FETAL CEREBROPLACENTAL DOPPLER INDICES IN CASES OF THREATENED PRETERM LABOUR. ALEXMED ePosters. 2023;5(2):13-14.	Study: wrong concept/intervention (MgSO4 for other indication)
Kumar DS, Kumari K, Mahaseth P. Maternal and Perinatal Outcome of Eclampsia in a Tertiary Care Center. International Journal of Pharmaceutical and Clinical Research. 2024;16(9):1646-1649.	Study: wrong concept/intervention (MgSO4 for other indication)
Tafere TZ, Aschalew AY, Tsehay CT, Gebremedhin T. Process Evaluation of Facility Delivery Services in Northwest Ethiopia: In the Case of Public Health Centers. International Journal of Women's Health. 2023;15:235-253.	Study: wrong concept/intervention (MgSO4 for other indication)
Fajolu IB, Mairami AB, Okonkwo I, et al. Rates and predictors of mortality of very low birthweight infants in three Nigerian tertiary hospitals. Acta Paediatrica, International Journal of Paediatrics. 2023;112(8):1766-1773.	Study: wrong concept/intervention (cannot determine MgSO4 indication)
Jiang SY, Yang CZ, Tian XY, et al. Outcomes and care practices of extremely preterm infants at 22-25 weeks' gestation age from the Chinese Neonatal Network. [Chinese]. Zhonghua er ke za zhi = Chinese journal of pediatrics. 2024;62(1):22-28.	Study: wrong concept/intervention (cannot determine MgSO4 indication)
Lin H, Yu Z, Huang J, et al. Delivery room resuscitation and short-term outcomes in very preterm infants: a multicenter cross-sectional study in China. Frontiers in Pediatrics. 2024;12(no pagination).	Study: wrong concept/intervention (cannot determine MgSO4 indication)
Mao W, Jiang S, Shen C, et al. Spontaneous intestinal perforation among very preterm infants in China: a multicenter cohort study. Translational Pediatrics. 2024;13(4):542-554.	Study: wrong concept/intervention (cannot determine MgSO4 indication)
Nayyar M, Sood M, Panwar PK. Profile and risk factors of sight-threatening retinopathy of prematurity: Experience from SNCU in North India. Oman Journal of Ophthalmology. 2024;17(2):224-233.	Study: wrong concept/intervention (cannot determine MgSO4 indication)
Venter M, Stassen W. A national retrospective descriptive analysis of critical care transfers in the private sector in South Africa. South African Medical Journal. 2023;113(9) (no pagination).	Study: wrong concept/intervention (cannot determine MgSO4 indication)
Zhang WW, Wang S, Li Y, et al. Development and validation of a model to predict mortality risk among extremely preterm infants during the early postnatal period: a multicentre prospective cohort study. BMJ Open. 2023;13(12) (no pagination).	Study: wrong concept/intervention (cannot determine MgSO4 indication)

Doddamani RM, Pujar TV, Patil GL, P AK, B R. A descriptive study on the risk factors of preterm birth with its maternal and fetal outcomes at a tertiary care hospital. 2023;10.	Study: wrong concept/intervention (not $MgSO_4$)

S3 Table. Characteristics of research studies (1)

Study ID	Format of publication	Language	Design	Dates of study	Funding, Declaration, Ethics, Prospective RCT registration	Setting, World Bank Classification	Aim/purpose	MgSO4 evidence cited
Abdel Fattah 2015 [1]	Trial registration	English	RCT	Start: July 2015<br>Completion: November 2015	Funding: NR<br>Declarations: NR<br>Ethical approval: NR<br>Prospective registration: Yes	Egypt<br>Lower-middle	"to find out if middle cerebral artery Doppler of the fetus can explain the mechanism behind the use of magnesium sulfate for fetal neuroprotection in preterm births."	Nil
Achola 2018 [2]	Conference abstract	English	Non-RCT<br><br>Not described (likely case series)	October 2016 to December 2017	Funding: NR<br>Declarations: NR<br>Ethical approval: NR	Kenya<br>Lower-middle	"Preterm Birth Initiative East Africa in Kenya modified the WHO checklist for use in 17 facilities in Migori County, Western Kenya. The goal of the modified SCC (mSCC) is to serve as both a quality improvement clinical-decision making tool as well as a data collection tool."	Nil
Atia 2023 [3]	Trial registration	English	RCT	Estimated start: January 2023<br>Estimated completion: July 2024	Funding: NR<br>Declarations: NR<br>Ethical approval: NR<br>Prospective registration: Yes	Egypt<br>Lower-middle	"Till now, there is a gap and lack of knowledge regarding the value of loading dose only as sufficient and effective strategy for neuroprotection compared to full therapy, which needs	Cochrane review: no citation, assumed Doyle 2009<br>Other relevant SRs/guidelines: FIGO 2021

Study ID	Format of publication	Language	Design	Dates of study	Funding, Declaration, Ethics, Prospective RCT registration	Setting, World Bank Classification	Aim/purpose	MgSO4 evidence cited
							more health costs, longer monitoring and carries more risk for the patients."	
Bachnas 2020 [4]	Conference abstract	English	RCT<br>Unclear: "were randomly grouped"	NR	Funding: NR<br>Declarations: NR<br>Ethical approval: NR<br>Prospective registration: NR	Indonesia<br>Upper-middle	"Whether a single bolus of 4g MgSO4 compared to 4g continued by 1g/ hour maintenance makes a difference to the level BDNF [brain derived neurotrophic factor] produced by fetus, needs to be clarified."	Nil
Bachnas 2022 [5]	Full-text publication	English	Non-RCT<br>Described as "cross sectional study" (likely prospective cohort)	October 2020 to February 2021	Funding: nil<br>Declarations: nil<br>Ethical approval: yes	Indonesia<br>Upper-middle	"Protocols for administering antenatal MgSO4 differ between countries and institutions. One of the concerns is whether the dose is given and the time interval to delivery has a big contribution towards a better fetal outcome. These relationships have not been studied before. This study aimed to analyze the correlation of the total dose of MgSO4 and time interval to delivery with BDNF levels in cord blood."	RCTs:<br>ACTOMgSO4 [no citation, BEAM [no citation]<br>Other relevant SRs/guidelines:<br>ACOG 2010, SOGC 2011, RCOG 2014 [assumed RCOG 2011]

Study ID	Format of publication	Language	Design	Dates of study	Funding, Declaration, Ethics, Prospective RCT registration	Setting, World Bank Classification	Aim/purpose	MgSO4 evidence cited
Bansal 2022 [6]	Full-text publication	English	Non-RCT<br>Described as "prospective observational comparative study" (likely prospective cohort)	April 2016 to March 2018	Funding: NR<br>Declarations: nil<br>Ethical approval: yes	India<br>Lower-middle	"1. To investigate the effectiveness of antenatal magnesium sulfate for neuroprotection in preterm infants between 26 and 34 weeks in preventing early neonatal morbidity and mortality. 2. To assess any adverse events with the use of magnesium sulfate on the mother and neonate…In spite of the recommendations, drug being inexpensive, clinicians experienced with its use in eclampsia, it is still not being widely practiced, and to my knowledge, there are no known Indian comparative studies on use of magnesium sulfate for neuroprotection."	Cochrane review: Doyle 2009<br>RCTs: Crowther 2003, Magpie 2007, Marret 2007/2008, Mittendorf 2002, Rouse 2008<br>Other relevant SRs/guidelines: ACOG 2010, Conde-Agudelo 2009, Costantine 2009, SOGC 2011, RCOG 2011
Cavalcanti 2018 [7]	Conference abstract	English	Non-RCT<br>Design not described (likely before-after study)	March to December 2016	Funding: NR<br>Declarations: NR<br>Ethical approval: NR	Brazil<br>Upper-middle	"In this study, we aimed to evaluate the role continuing medical education as the way to implement the current	Nil

Study ID	Format of publication	Language	Design	Dates of study	Funding, Declaration, Ethics, Prospective RCT registration	Setting, World Bank Classification	Aim/purpose	MgSO4 evidence cited
							recommendations in intrapartum care.”	
Chandran 2021 [8]	Full-text publication	English	Non-RCT<br>Prospective cohort study	November 2016 to August 2017	Funding: supported by a fluid research grant from the Institutional Review Board, Christian Medical College Vellore<br>Declarations: nil<br>Ethical approval: yes	India<br>Lower-middle	“Recently antenatal MgSO4 has become the standard of care for preterm neuroprotection and after its wide usage for neuroprotection, there are no prospective cohort studies which studied the effects of antenatal MgSO4 on the preterm gut. So, this study was planned to clarify the effects of antenatal MgSO4 on feed intolerance and blood flow integrity of the gut on preterm/VLBW neonates.”	Other relevant SRs/guidelines: ACOG 2010
Daneji 2022 [9]	Full-text publication	English	Non-RCT<br>Described as “a retrospective audit” (likely cross-sectional study)	January 2018 to December 2019	Funding: NR<br>Declarations: nil<br>Ethical approval: NR	Nigeria<br>Lower-middle	“This study aims to audit the management of Preterm PROM in Aminu Kano Teaching Hospital. It will give us a guide on how PPROM is managed in the hospital. This will improve care and will decrease the morbidity and mortality associated with PPROM.”	Other relevant SRs/guidelines: ACOG 2020 [PROM], RCOG 2019 [PPROM]

Study ID	Format of publication	Language	Design	Dates of study	Funding, Declaration, Ethics, Prospective RCT registration	Setting, World Bank Classification	Aim/purpose	MgSO4 evidence cited
Diggikar 2021 [10]	Full-text publication	English	Non-RCT<br>Cross-sectional study (survey)	December 2019 to February 2020	Funding: nil<br>Declarations: nil<br>Ethical approval: not required	India<br>Lower-middle	"As there is no national registry on neonatal practices that can capture all centers across India, the data on the uptake of antenatal magnesium sulfate is sparse. Therefore, we aimed to know the practices of antenatal magnesium sulfate usage and its challenges across India through a survey"	Other relevant SRs/guidelines: Crowther 2017, FOGSI 2017, WHO 2015b [no date cited]
Dolly 2022 [11]	Full-text publication	English	Non-RCT<br>Described as "retrospective observational study" (likely non-comparative study – case series)	March 2019 to December 2020	Funding: NR<br>Declaration: NR<br>Ethical approval: yes	India<br>Lower-middle	"Recently, a few studies have indicated that the use of magnesium sulfate has a neuroprotective effect on the neonate, but it has some toxic effects when used for an extended period of time. The goal of this study is to assess the neonatal outcomes of preterm births, because it is still unclear how to describe the neonatal outcomes with the use of magnesium sulphate during preterm labor"<br><br>"This study basically focuses on analyzing	Other relevant SRs/guidelines: Bain 2013

Study ID	Format of publication	Language	Design	Dates of study	Funding, Declaration, Ethics, Prospective RCT registration	Setting, World Bank Classification	Aim/purpose	MgSO4 evidence cited
							the neonatal outcomes with the use of magnesium sulphate which was administered to women who undergone preterm labor in our hospital, as there is less number of studies available in India regarding the maternal use of magnesium sulphate to correlate the association of maternal effects and analyzing the neonatal outcomes with the drug."	
Gathwala 2018 [12]	Trial registration	English	RCT	Date of first enrolment: June 2018 (recruitment status "Not Yet Recruiting")	Funding: "Source of Monetary or Material Support: Pt. B.D. Sharma, PGIMS, Rohtak"<br>Declaration: NR<br>Ethical approval: yes<br>Prospective registration: yes	India<br>Lower-middle	NR; as per title and outcomes, to assess the "Role of Magnesium sulphate to mother in protecting brain of preterm babies"	Nil
Gupta 2021 [13]	Full-text publication	English	RCT	August 2018 to December 2020	Funding: nil<br>Declaration: nil<br>Ethical approval: NR<br>Prospective registration: NR	India<br>Lower-middle	"To assess the effect of MgSO4 given for fetal neuroprotection to women at risk of preterm birth."	RCTs: Crowther 2003, Marret 2008, Rouse 2009<br>Other relevant SRs/guidelines: Conde-Agudelo 2009

Study ID	Format of publication	Language	Design	Dates of study	Funding, Declaration, Ethics, Prospective RCT registration	Setting, World Bank Classification	Aim/purpose	MgSO4 evidence cited
Gupta 2023 [14]	Full-text publication	English	Non-RCT "randomization study" "patients were divided into two group each having 50 women" (likely quasi-RCT)	September 2017 to October 2018	Funding: nil<br>Declaration: nil<br>Ethical approval: yes	India<br>Lower-middle	"Aim of this study was to compare the perinatal outcome of preterm deliveries between women receiving magnesium sulphate and those who were not received magnesium sulphate and to evaluate the decrease in preterm morbidity."	RCTs: Rouse 2009<br>Other relevant SRs/guidelines: Conde-Agudelo 2009, Zeng 2016
Iqbal 2023 [15]	Full-text publication	English	Non-RCT "Prospective observational research" (likely prospective cohort study)	January 2020 to December 2022	Funding: nil<br>Declaration: nil<br>Ethical approval: yes	Pakistan<br>Lower-middle	"To evaluate the impact of MgSO4 administered to pregnant women at risk for premature delivery for fetal neuroprotection."<br><br>"Despite the recommendation, low cost and the availability of doctors with experience using it in eclampsia, to the best of our knowledge, no comparison research on the use of magnesium sulfate for neuroprotection in Pakistan in recent years, and it is still not widely used. Therefore, we have	RCTs: Crowther 2003, Marret 2008, Rouse 2009<br>Other relevant SRs/guidelines: Agustin 2009 [Conde-Agudelo 2009], ACOG 2010 [no citation]

Study ID	Format of publication	Language	Design	Dates of study	Funding, Declaration, Ethics, Prospective RCT registration	Setting, World Bank Classification	Aim/purpose	MgSO4 evidence cited
							decided to establish the use of magnesium sulfate for fetal neuroprotection in patients with expected premature delivery as the norm in our unit at a tertiary care institution."	
Jamileh 2018 [16]	Full-text publication	English	Non-RCT "Before-after study"	April 2016 to May 2017	Funding: NR<br>Declaration: nil<br>Ethical approval: yes	Iran<br>Lower-middle	"The aim of this study was to assess the effect of MgSO4 on fetal middle cerebral artery, umbilical artery and uterine artery by using Doppler parameters in pregnancy up to 32 weeks."	RCTs: Altman 2002
Jayashree 2021 [17]	Full-text publication	English	Non-RCT "Prospective observational study" (likely case series)	October 2019 to October 2021	Funding: NR<br>Declarations: NR<br>Ethical approval: NR	India<br>Lower-middle	"the present study aimed to investigate the effectiveness of antenatal magnesium sulphate for neuroprotection in preterm infants between 26 and 34 weeks in preventing early neonatal morbidity and mortality. Secondary objective was to assess any adverse events with the use of magnesium sulphate on	WHO 2015<br>Cochrane review: Doyle 2009<br>RCTs: Crowther 2003, Magpie 2007, Marret 2008, Mittendorf 2002, Rouse 2008<br>Other relevant SRs/guidelines: Bain 2012, Bain 2013, Costantine 2009, Conde-Agudelo 2009

Study ID	Format of publication	Language	Design	Dates of study	Funding, Declaration, Ethics, Prospective RCT registration	Setting, World Bank Classification	Aim/purpose	MgSO4 evidence cited
							the mother and neonate.”	
Jin 2022 [18]	Full-text publication	English	Non-RCT (likely retrospective cohort study)	January 2019 to December 2019	Funding: “supported by the Canadian Institutes of Health Research (CTP87518) and the Chinese Neonatal Network coordinating center” Declarations: nil Ethical approval: yes	China Upper-middle	“During the past decade, several studies have reported that select maternal and newborn practices can improve preterm birth outcomes, including… prenatal MgSO4 usage... These 4 practices have been shown to be effective in clinical trials and are widely used clinically. However, the efficacy of clinical interventions in the real world is often different from randomized controlled trials since population risks may differ and multiple interventions may coexist. Our objective was to examine the individual and cumulative effects of these 4 evidence-based clinical practices in the VPI population in China and on their subpopulation of extremely preterm	WHO 2015 Cochrane review: Doyle 2009 Other relevant SRs/guidelines: Crowther 2017

Study ID	Format of publication	Language	Design	Dates of study	Funding, Declaration, Ethics, Prospective RCT registration	Setting, World Bank Classification	Aim/purpose	MgSO4 evidence cited
							infants (EPI) born at 24–27 weeks GA.”	
Kasapoğlu 2020 [19]	Full-text publication	English	Non-RCT Retrospective cohort study	January 2015 to January 2017	Funding: nil<br>Declaration: nil<br>Ethical approval: yes	Turkey<br>Upper-middle	“Considering the widespread use of AAM [antenatal administration of magnesium sulphate] in obstetric care, the aim of the present study was to assess the potential neuroprotective effect of antenatal MgSO4 on auditory nerve development and sensorineural hearing in premature newborns.”	Cochrane review: Doyle 2009<br>RCTs: Crowther 2003<br>Other relevant SRs/guidelines: ACOG 2013
Kumar 2025 [20]	Full-text publication	English	Non-RCT Prospective cohort study	May 2019 to May 2020 with follow up of children to 3 years	Funding: nil<br>Declaration: nil<br>Ethical approval: yes	India<br>Lower-middle	“The use of magnesium sulphate as a neuroprotective agent had been a very appreciable development in obstetrics; however, still its use is not universal in all heath care settings especially in developing countries owing to the fear of side effects to the mother. Administration of 24 h intravenous dose is difficult in small settings; hence,	WHO 2015<br>Cochrane review: Doyle 2009<br>RCTs: Mittendorf 2002, Rouse 2008<br>Other relevant SRs/guidelines: Conde-Agudelo 2009, SOGC 2019, Moradi 2020, FIGO 2021, Shepherd 2019, Zeng 2016

Study ID	Format of publication	Language	Design	Dates of study	Funding, Declaration, Ethics, Prospective RCT registration	Setting, World Bank Classification	Aim/purpose	MgSO4 evidence cited
							there is a need to evaluate benefit of lower dose magnesium so that we can escape from side effects and gain neuroprotective benefits. Efficacy of lower dose can of great help to encourage its use in all low resource heath care settings which do encounter the problem preterm deliveries but do not have enough resources or expertise to monitor while giving a high dose of magnesium given to mother. Hence, the study was planned to evaluate what is the difference in neonatal outcome comparing magnesium sulphate in small dose (4 gram intravenous infusion over 20 min) in established preterm labour versus those who did not receive magnesium sulphate (controls)."	
Mamatha 2023 [21]	Full-text publication	English	Non-RCT Described as an "observational	November 2020 to	Funding: NR Declarations: NR Ethical approval: NR	India Lower-middle	"1. To assess the effectiveness of magnesium sulphate	WHO guideline [no citation]

Study ID	Format of publication	Language	Design	Dates of study	Funding, Declaration, Ethics, Prospective RCT registration	Setting, World Bank Classification	Aim/purpose	MgSO4 evidence cited
			study" (likely case series)	November 2021			for fetal neuroprotection in pregnancy less than 34 weeks of gestation.<br>2. To report maternal and fetal adverse events during magnesium sulphate usage"	Cochrane review: [no citation, assumed Doyle 2009]<br>RCTs: ACTOMgSO4 [no citation], BEAM [no citation], MagNET [no citation], PREMAG [no citation], Crowther 2013 [MAGENTA])<br>Other relevant SRs/guidelines: ACOG [no citation], RANCOG [no citation - presumed RANZCOG], RCOG 2011, RCOG 2019, SA Perinatal Practice Guideline [no citation], Shepherd 2019, FIGO 2021, SOGC [no citation]
Manoj 2017 [22]	Full-text publication	English	RCT	February 2014 to November 2015	Funding: nil<br>Declaration: nil<br>Ethical approval: yes<br>Prospective registration: NR	India<br>Lower-middle	"While several western countries have formulated guidelines on the use of MgSO4 as a neuroprotective agent, there are very few Indian studies regarding the antenatal use of MgSO4 as a neuroprotective agent."	Cochrane review: Doyle 2009<br>RCTs: Crowther 2003, Marret 2008, Rouse 2008

Study ID	Format of publication	Language	Design	Dates of study	Funding, Declaration, Ethics, Prospective RCT registration	Setting, World Bank Classification	Aim/purpose	MgSO4 evidence cited
							"At present, Indian protocols do not recommend routine antenatal administration of MgSO4 to mothers at risk of preterm delivery."<br>"MgSO4 is inexpensive and commonly used in the management of pre-eclampsia and for tocolysis. We undertook this study to test the hypothesis that antenatal MgSO4 has a neuroprotective role in the early neonatal period, when given to women considered at risk for preterm delivery."	
Marnal 2023 [23]	Full-text publication	English	Non-RCT<br>"Retrospective audit"	July 2020 to December 2020; and July 2021 to December 2021	Funding: NR<br>Declaration: nil<br>Ethical approval: NR	India<br>Lower-middle	"retrospective audit…" (across two periods)<br>"Changes in clinical practice were noted"	WHO 2015<br>Other relevant SRs/guidelines: ACOG [no citation], FOGSI 2017, NHS 2020, NICE 2015/2019, RCOG 2019, SOGC 2011
Mazhar 2023 [24]	Full-text publication	English	Non-RCT<br>"Cross-sectional descriptive study" (likely case series)	December 2020 to November 2021	Funding: nil<br>Declaration: nil<br>Ethical approval: NR	Pakistan<br>Lower-middle	"The study aimed to find the effect of 4 grams of intravenous bolus antenatal dose of	WHO [no citation]<br>Cochrane review: [no citation,

Study ID	Format of publication	Language	Design	Dates of study	Funding, Declaration, Ethics, Prospective RCT registration	Setting, World Bank Classification	Aim/purpose	MgSO4 evidence cited
							magnesium Sulphate on maternal and neonatal outcomes in preterm births."<br>"This study is aimed at determining the effectiveness of Magnesium Sulphate in preventing fetal and maternal outcomes"	assumed Doyle 2009]<br>RCTs: Crowther 2013, Doyle 2014, Marret 2007, Mittendorf 2002, Rouse 2008<br>Other relevant SRs/guidelines: 'Australian guidelines' [no citation], Conde-Agudelo 2009, Costantine 2009, Galinsky 2020, Jayaram 2019, NHS 2019, FIGO 2021
Medhi 2023 [25]	Full-text publication	English	Non-RCT<br>"Prospective observational study"<br>(likely prospective cohort study)	September 2020 to August 2021	Funding: NR<br>Declaration: Nil<br>Ethical approval: NR	India<br>Lower-middle	"To assess the effectiveness of magnesium sulfate as a neuroprotective agent in early preterm labour (28 to 32 weeks). To assess any maternal or fetal adverse effects after giving magnesium sulfate."	RCTs: Crowther 2003, Marret 2007, Rouse 2008<br>Other relevant SRs/guidelines: Conde-Agudelo 2009, FOGSI 2017
Millogo-Traoré 2022 [26]	Full-text publication	English	Non-RCT<br>Prospective cohort study	March to September 2020	Funding: NR<br>Declaration: nil<br>Ethical approval: unclear	Burkina Faso<br>Low-income	"evaluate the fetal neuroprotection protocol using sulfate magnesium during births before thirty-three (33) weeks of amenorrhea at the University Health	WHO 2015<br>Cochrane review: Doyle 2009<br>RCTs: Crowther 2003, Marret 2007 & 2008, Rouse 2008<br>Other relevant SRs/guidelines:

Study ID	Format of publication	Language	Design	Dates of study	Funding, Declaration, Ethics, Prospective RCT registration	Setting, World Bank Classification	Aim/purpose	MgSO4 evidence cited
							Centers (UHC) of Yalgado Ouedraogo and Bogodogo in Ouagadougou, Burkina Faso."<br>"Magnesium sulphate given in imminent preterm delivery has been shown to be effective in preventing the neurological sequelae. In sub-Saharan Africa, few studies have been conducted on this subject, and we have undertaken this study in Burkina Faso."	Costantine 2009, Crowther 2017
Mohan 2023 [27]	Full-text publication	English	Non-RCT Prospective cohort study	June 2021 to December 2022	Funding: NR<br>Declaration: NR<br>Ethical approval: Yes	India<br>Lower-middle	"To determine the role of magnesium sulphate given for fetal neuro protection to women at risk of preterm birth in preventing neonatal mortality and neuro developmental morbidity."	RCTs: Altman 2002, Crowther 2003, MAGENTA [no citation], Marret 2007, MASP [no citation], Rouse 2008<br>Other relevant SRs/guidelines: ACOG 2016
Muhammed 2019 [28]	Full-text publication	English	RCT	June 2018 to January 2019	Funding: NR<br>Declaration: NR<br>Ethical approval: unclear<br>Prospective registration: NR	Iraq<br>Upper-middle	"To assess neuroprotective effect of maternal intravenous administration of magnesium sulphate on neurological outcome	Cochrane review: Doyle 2009<br>RCTs: Doyle 2014, Hirtz 2015<br>Other relevant SRs/guidelines: Bain 2012, Bain

Study ID	Format of publication	Language	Design	Dates of study	Funding, Declaration, Ethics, Prospective RCT registration	Setting, World Bank Classification	Aim/purpose	MgSO4 evidence cited
							of neonates in preterm deliveries."	2013, Costantine 2009, Crowther 2017, NICE Guidelines 2015, Zeng 2016
Musiime 2021 [28]	Full-text publication	English	Non-RCT Retrospective cohort study	January to December 2016	Funding: nil<br>Declaration: nil<br>Ethical approval: yes	South Africa Upper-middle	"There is a need for recent data focusing on both long- and short-term outcomes of ELBW neonates in SA to guide forward planning, resource allocation and policy development with a view to optimising outcomes and decreasing mortality." "The purpose of the present study was to describe the morbidity and mortality of ELBW neonates treated at Tygerberg Hospital (TBH) in 2016."	Nil
Nunes 2018 [30]	Full-text publication	English	Non-RCT Cross-sectional study	September 2009 to January 2014	Funding: NR<br>Declaration: Nil<br>Ethical approval: yes	Brazil Upper-middle	"Cerebral palsy is often associated with prematurity and magnesium sulfate (MgSO4) has been used as a neuroprotector, with favorable results. However, its mechanism of action	RCTs: Crowther 2003, Marret 2007 & 2008, Rouse 2008<br>Other relevant SRs/guidelines: Costantine 2009

Study ID	Format of publication	Language	Design	Dates of study	Funding, Declaration, Ethics, Prospective RCT registration	Setting, World Bank Classification	Aim/purpose	MgSO4 evidence cited
							has not been fully elucidated. This study aimed to evaluate the association between MgSO4 at the imminent premature delivery and neonatal hemodynamic effects."	
Okulu 2024 [31]	Full-text publication	English	Non-RCT "Retrospective observational study" (likely retrospective cohort study)	January 2016 to June 2022	Funding: nil<br>Declarations: nil<br>Ethical approval: yes	Turkey<br>Upper-middle	"Magnesium sulfate (MgSO4) provides effective fetal neuroprotection. However, there is conflicting evidence regarding the association between antenatal MgSO4 exposure and patent ductus arteriosus (PDA)… In this study, we aimed to evaluate infants who were exposed to antenatal MgSO4 and determine the association between antenatal MgSO4 exposure and a hemodynamically significant PDA (hsPDA) as well as other short-term neonatal outcomes."	Cochrane review: Doyle 2009<br>RCTs: Mittendorf 2002<br>Other relevant SRs/guidelines: ACOG 2016, Conde-Agudelo 2009, Costantine 2009, SOGC 2011, SOGC 2019
Ozer Bekmez 2021 [32]	Full-text publication	English	Non-RCT "Prospective observational	January 2017 to	Funding: NR<br>Declaration: nil<br>Ethics approval: yes	Turkey<br>Upper-middle	"Antenatal magnesium sulfate (MgSO4) treatment is associated	Cochrane review: Doyle 2009

Study ID	Format of publication	Language	Design	Dates of study	Funding, Declaration, Ethics, Prospective RCT registration	Setting, World Bank Classification	Aim/purpose	MgSO4 evidence cited
			study" (likely prospective cohort study)	February 2018			with reduced risk of cerebral palsy in preterm infants. We aimed to investigate whether this treatment leads to any alterations on cerebral hemodynamics which could be detected by near-infrared spectroscopy (NIRS) readings in early postnatal life."	RCTs: Crowther 2003, Marret 2007, Rouse 2008<br>Other relevant SRs/guidelines: Conde-Agudelo 2009, Costantine 2009, Crowther 2017, Zeng 2016, SOGC 2011
Ozer Bekmez 2023 [33]	Full-text publication	English	Non-RCT<br>Described as "Prospective observational study" (likely prospective cohort study)	2017 to 2021	Funding: NR<br>Declarations: nil<br>Ethical approval: yes	Turkey<br>Upper-middle	"Magnesium sulfate (MgSO4) treatment is widely used for fetal neuroprotection despite the controversy concerning the side effects. There is limited data regarding the impact of various cumulative maternal doses and neonatal serum magnesium (Mg) levels on short-term neonatal morbidity and mortality. We opted to carry out a study to determine the impact of neonatal serum Mg levels on neonatal outcomes."	Cochrane review: Doyle 2009<br>RCTs: Crowther 2003, Marret 2008, Rouse 2008<br>Other relevant SRs/guidelines: Shepherd 2019

Study ID	Format of publication	Language	Design	Dates of study	Funding, Declaration, Ethics, Prospective RCT registration	Setting, World Bank Classification	Aim/purpose	MgSO4 evidence cited
Özlü 2019 [34]	Full-text publication	English	"Retrospective cohort study" (non-concurrent cohort)	2011-2012 and 2014-2016	Funding: nil<br>Declaration: nil<br>Ethical approval: yes	Turkey<br>Upper-middle	"The effect of magnesium sulphate (MgSO4) to mothers with imminent premature birth at <34 weeks on neonatal morbidities like intraventricular hemorrhage, feeding intolerance, retinopathy or bronchopulmonary dysplasia is not clear. We evaluated the effect of antenatal magnesium sulfate exposure on premature early and late morbidities and mortality retrospectively."<br>"Although some national guidelines based on relevant clinical evidence present, ongoing controversies about aspects of this therapy remain."<br>"In our unit, MgSO4 has been used as an anti- eclamptic agent, but not as a neuroprotective agent until recently."	Cochrane review: Doyle 2009<br>RCTs: Mittendorf 2002, Crowther 2003, Marret 2007<br>Other relevant SR/guidelines: Conde-Agudelo 2009, Costantine 2009, Zeng 2016

Study ID	Format of publication	Language	Design	Dates of study	Funding, Declaration, Ethics, Prospective RCT registration	Setting, World Bank Classification	Aim/purpose	MgSO4 evidence cited
Padmapriya 2023 [35] (and Niveditha 2020) [58]	Full-text publication and thesis	English	Non-RCT (unclear if case series/prospective cohort)	NR	Funding: nil<br>Declaration: nil<br>Ethical approval: NR in publication; yes in thesis	India<br>Lower-middle	"To understand the risk of neurological disability in preterm babies. To understand the role of MgSO4 for neuroprotection in preterm deliveries and its effect in preventing neurological outcomes. To evaluate the feasibility of its application in all mothers with preterm labour in future."	NR in publication (mentioned "recently issued guidelines" in text [no citation])<br><br>Thesis:<br>WHO guideline [no citation]<br>Cochrane review 2009 [no citation]<br>RCTs: ACTOMgSO4 [no citation], BEAM [no citation], PREMAG [no citation]
Pandey 2024 [36]	Full-text publication	English	Non-RCT "Observational study" (likely case series)	NR (2 years)	Funding: NR<br>Declaration: NR<br>Ethical approval: NR	India<br>Lower-middle	"To evaluate the use of antenatal Mgso4 for neuroprotection of preterm infants."	RCTs: ACTOMgSO4, BEAM, MagNET, PREMAG, [no citations]<br>Other relevant SRs/guidelines: RCOG 2011
Parashi 2017 [37]	Full-text publication	English	RCT	NR (expected dates in trial registration were: 2015-2016)	Funding: NR in paper; yes in trial registration<br>Declaration: NR<br>Ethical approval: yes<br>Prospective registration: no (retrospectively registered)	Iran<br>Lower-middle	"Therefore, this study aimed to investigate the effect of magnesium sulfate on intraventricular hemorrhage in infants of mothers with premature rupture of membranes." "Hence, regarding the extended use of magnesium	RCTs: Marret 2015, Rouse 2008/Hirtz 2015, Crowther et al. [no citation]

Study ID	Format of publication	Language	Design	Dates of study	Funding, Declaration, Ethics, Prospective RCT registration	Setting, World Bank Classification	Aim/purpose	MgSO4 evidence cited
							sulfate in health care centers of our country, the outcomes of IVH in premature neonates were assessed in this clinical trial.”	
Patel 2024 [38]	Full-text publication	English	Non-RCT “Retrospective prospective cohort study” (unclear if retrospective or prospective)	January to December 2021	Funding: nil<br>Declaration: nil<br>Ethical approval: NR	India<br>Lower-middle	“The study was conducted to determine effects of antenatal steroids along with magnesium sulphate on Intraventricular Hemorrhage and Periventricular Leukomalacia in neonates born below 32 weeks of gestation compared to antenatal steroids alone and without antenatal steroids or magnesium sulphate.”	RCTs: Crowther 2013
Peng 2024 [39]	Full-text publication	English	Non-RCT "Retrospective observational study" (likely retrospective cohort study)	January 2013 to December 2022	Funding: “financial support was received for the research, authorship, and/or publication of this article. The study was funded by the Maternal and Infant Nutrition and Health Research Project of the Maternal and Child Center of the Chinese Center for	China<br>Upper-middle	“this study aims to conduct the above-mentioned comparison [inborn versus postnatal transfer of extremely preterm infants in China] through a real world retrospective study, in order to provide support to the practice of in-utero transfer of EPIs.”	Nil

Study ID	Format of publication	Language	Design	Dates of study	Funding, Declaration, Ethics, Prospective RCT registration	Setting, World Bank Classification	Aim/purpose	MgSO4 evidence cited
					Disease Control and Prevention (2022FYH002, to SX); the National Natural Science Foundation of China (82001603, to SP); the Research Projects of the Maternal and Child Health Hospital of Hubei Province (230741005, to SP); and the Public Health Leading Talents Training Program of Hubei Province (NO. 1020013003, to SX). The funders are not involved in either the preparation of the data or the manuscript."<br>Declarations: nil<br>Ethical approval: yes			
Phrasidthideth 2021 [40]	Abstract (full-text publication not available)	English	Non-RCT<br>"retrospective cross-sectional study"	July to November 2017	Funding: NR<br>Declaration: NR<br>Ethics approval: NR	Lao PDR<br>Lower-middle	"we aimed to investigate the possible causes of death among preterm and LBW infants admitted to the post-delivery ward and/or NICU at Mahosot Hospital, Lao PDR, and the possible factors that influence	Nil

Study ID	Format of publication	Language	Design	Dates of study	Funding, Declaration, Ethics, Prospective RCT registration	Setting, World Bank Classification	Aim/purpose	MgSO4 evidence cited
							preterm and LBW infants' survival."	
Pirjani 2019 [41]	Trial registration	English	RCT	Date of registration: 2019-12-26<br>Date of first enrolment: 2019-12-22<br>Date of last refreshment: 2020-01-13<br>Status: recruiting	Funding: "Tehran University of Medical Sciences"<br>Declaration: NR<br>Ethical approval: yes<br>Prospective registration: no	Iran<br>Lower-middle	"Evaluation of neuroprotective effects of magnesium sulfate on preterm infants with a gestational age of 32-36 weeks"	Nil
Rauf 2017 [42]	Full-text publication	English	Non-RCT (likely retrospective cohort study)	January 2011 to February 2016	Funding: NR<br>Declaration: NR<br>Ethical approval: yes	Turkey<br>Upper-middle	"In Turkey, although magnesium sulfate has been licensed for the treatment of severe preeclampsia, eclampsia, and tocolysis, there is no approval for fetal neuroprotection indication. Also, there is still no standard protocol for MgSO4 use for fetal neuroprotective effect at many centers in Turkey. We have established the standard treatment protocol for the purpose of fetal neuroprotection in our clinic since April 2014.	Cochrane review: Doyle 2009<br>RCTs: Crowther 2003, Marret 2007, Mittendorf 2001, Rouse 2008<br>Other relevant SRs/guidelines: ACOG 2013, Bain 2012, Costantine 2011

Study ID	Format of publication	Language	Design	Dates of study	Funding, Declaration, Ethics, Prospective RCT registration	Setting, World Bank Classification	Aim/purpose	MgSO4 evidence cited
							In this study, we aimed to demonstrate the effect of MgSO4 for fetal neuroprotection on maternal and neonatal outcomes of pregnants delivered before 32 weeks and compare results with untreated patients."	
Rivera-Rueda 2017 [43]	Full-text publication	Spanish	Non-RCT "analysis of a cohort of neonates consecutively" (likely retrospective cohort study)	January to December 2016	Funding: NR<br>Declaration: nil<br>Ethics: NR	Mexico<br>Upper-middle	"The objective of the study was to determine morbidity and mortality in neonates <1,500 g at birth discharged from intensive care neonatal from a tertiary care institution."	RCTs: Crowther 2003, Marret 2007, Rouse 2008
Sarath 2021 [44]	Full-text publication	English	Non-RCT "Prospective study" (unclear if single arm trial or prospective cohort study)	May 2019 to April 2020	Funding: nil<br>Declaration: nil<br>Ethical approval: yes	India<br>Lower-middle	"Antenatal magnesium sulphate is one of the neuro protective strategies used for preterm deliveries. One of the concerns with this is whether it leads to hypermagnesemia in newborns."<br>"The primary objective of this study was to study the postnatal trends of magnesium in babies whose mothers received antenatal magnesium sulphate	Other relevant SRs/guidelines: Crowther 2017

Study ID	Format of publication	Language	Design	Dates of study	Funding, Declaration, Ethics, Prospective RCT registration	Setting, World Bank Classification	Aim/purpose	MgSO4 evidence cited
							for preterm neuro-protection. The secondary objectives were to compare serum magnesium levels in babies with and without the following selected morbidities: 1. Need for delivery room resuscitation 2. Need for mechanical ventilation within 24 hours 3. Hemodynamically significant PDA 4. Need for inotropes within 24 hours of life 5. Pulmonary hemorrhage 6. Hypocalcemia 7. Sepsis 8. Necrotising enterocolitis 9. Abnormal neurosonogram findings 10. Mortality"	
Sariati 2017 [45]	Full-text publication	Indonesian	Non-RCT "Analytic observational research with cross-sectional approach" (unclear if retrospective cohort or cross-sectional study)	January to March 2017	Funding: NR Declaration: NR Ethical approval: NR	Indonesia Lower-middle	"This study aims to determine the effect of a history of the use of magnesium sulphate in the mother during antenatal care against gross motor development in children aged 2-3 years"	RCTs: Crowther 2003 Other relevant SRs/guidelines: ACOG 2016, Conde-Agudelo 2009, SOGC 2011, Walker 2010 [summary of ANZ Guidelines])

Study ID	Format of publication	Language	Design	Dates of study	Funding, Declaration, Ethics, Prospective RCT registration	Setting, World Bank Classification	Aim/purpose	MgSO4 evidence cited
Sharma 2021 [46]	Full-text publication	English	RCT	January 2019 to 2020	Funding: NR<br>Declaration: NR<br>Ethical approval: NR<br>Prospective RCT registration: NR	India<br>Lower-middle	"Strong evidence from five randomized controlled trials and five meta-analyses has demonstrated that magnesium sulfate, when administered before preterm delivery, significantly reduces the risk of neurological disabilities… Aim of this study is to determine the effectiveness of magnesium sulphate given for neuroprotection to women at risk of preterm birth before 32 week gestation in preventing neurological deficit"	WHO 2015 [no citation]<br>Cochrane review: Doyle 2009<br>RCTs: Crowther 2003, Marret 2008, Mittendorf 2002, Rouse 2009<br>Other relevant SRs/guidelines: ('the university of Adelaide' [no citation], FIGO 2015 [no citation], France 2017 [no citation]
Sheeba 2022 [47]	Full-text publication	English	RCT	December 2015 to May 2016	Funding: nil<br>Declaration: nil<br>Ethical approval: yes<br>Prospective RCT registration: NR	India<br>Lower-middle	"Although several developed countries have formulated guidelines for antenatal administration of MgSO4 for neuroprotection, only a few Indian studies, and still less from Kerala have been conducted to assess the efficacy of	Cochrane review: Doyle 2009<br>RCTs: Crowther 2003, Magpie 2007<br>Other relevant SRs/guidelines: Conde-Agudelo 2009, Costantine 2009, Galinsky 2020, Jayaram 2019, FIGO 2021, Shepherd 2019

Study ID	Format of publication	Language	Design	Dates of study	Funding, Declaration, Ethics, Prospective RCT registration	Setting, World Bank Classification	Aim/purpose	MgSO4 evidence cited
							the same in our population" "The present study aimed to identify whether preterm babies whose mothers received MgSO4 had a better survival at discharge, and neurodevelopmental outcome at six months of age, when compared to those whose mothers did not." "To assess the role of administration of MgSO4 in improving neurodevelopmental outcome in preterm babies."	
Sulistyowati 2021 [48]	Full-text publication	English	Non-RCT "quantitative analytic study using a cross-sectional design"	July 2020 to November 2020	Funding: nil<br>Declaration: nil<br>Ethical approval: yes	Indonesia<br>Upper-middle	"The strategy to decrease CP incidence is by giving antenatal magnesium sulphate (MgSO4) prior to the inevitable preterm delivery. The neuroprotective effects of MgSO4 are driven by the stimulation of magnesium on Brain-Derived Neurotrophic Factor (BDNF) production…. Aim: To investigate the	Cochrane review: Doyle 2009<br>RCTs: Mittendorf 2002

Study ID	Format of publication	Language	Design	Dates of study	Funding, Declaration, Ethics, Prospective RCT registration	Setting, World Bank Classification	Aim/purpose	MgSO4 evidence cited
							correlation between maternal serum and umbilical cord blood magnesium levels over the umbilical cord blood BDNF levels.	
Tom 2018 [49]	Full-text publication	English	RCT<br>“randomized controlled trial” (described as RCT, unclear methodology)	January to December 2015	Funding: nil<br>Declaration: nil<br>Ethical approval: yes<br>Prospective RCT registration: NR	India<br>Lower-middle	“Even though there are guidelines regarding use of magnesium sulphate in pregnant women at risk of preterm labour for neuroprotection of fetus in several developed nations, none exist in India in this regard.”<br>“Present study aims to look at the effect of antenatal magnesium sulphate administration and milking of umbilical cord during delivery in the incidence of IVH in preterm infants.”	WHO [no citation]<br>Cochrane review: Doyle 2009<br>RCTs: Crowther 2013, Rouse 2008<br>Other relevant SRs/guidelines: Doyle 2009; IOG 2013
Tummala 2024 [50]	Full-text publication	English	Non-RCT<br>"Prospective observational study” (likely prospective cohort)	October 2020 to September 2022	Funding: “Financial support obtained from University, in collaborations with Department of Neonatology and Department of Biochemistry for	India<br>Lower-middle	“The effectiveness of MgSO4 for foetal neuroprotection is acknowledged, but the best time to provide it in relation to birth is a conundrum, and dose schedule is yet unknown.	Cochrane review: Doyle 2009<br>RCTs: ACTOMgSO4 [no citation], BEAM [no citation], Crowther 2013, PREMAG [no citation]

Study ID	Format of publication	Language	Design	Dates of study	Funding, Declaration, Ethics, Prospective RCT registration	Setting, World Bank Classification	Aim/purpose	MgSO4 evidence cited
					retrieving data and for the blood test" Declaration: nil Ethical approval: yes		Understanding the determinants of the magnesium levels in cord blood aids in determining the appropriate timing and length of administration… To assess the cord blood magnesium concentration in relation to the timing of MgSO4 and delivery. To achieve ROC in relation to optimum level of cord blood magnesium concentration in relation to neonatal outcome variables."	Other relevant SRs/guidelines: SOGC 2011, ACOG 2010, FIGO 2021, UoA 2010, Crowther 2017
Üstün 2021 [51]	Full-text publication	English	Non-RCT Retrospective cohort study	NR	Funding: nil Declaration: nil Ethical approval: yes	Turkey Upper-middle	"Based on the available evidence, some national guidelines have recommended antenatal MgSO4 for fetal neuroprotection. However, several studies raised concerns about the possible adverse effects of antenatal MgSO4 in terms of neonatal morbidities." "In our unit, MgSO4 is	RCTs: Crowther 2003, Marret 2008, Mittendorf 2002, Rouse 2008 Other relevant SRs/guidelines: Shepherd 2017, ACOG 2010

Study ID	Format of publication	Language	Design	Dates of study	Funding, Declaration, Ethics, Prospective RCT registration	Setting, World Bank Classification	Aim/purpose	MgSO4 evidence cited
							administered for fetal neuroprotection in preterm deliveries. This study aimed to investigate the effects of antenatal MgSO4 exposure on cardiorespiratory complications during the early neonatal period in premature infants."	
Vaz Ferreira 2024 [52]	Full-text publication	English	Non-RCT "Retrospective multicentre cohort study, with prospective data entry" (retrospective cohort study)	January 2015 to December 2020	Funding: NR<br>Declaration: nil<br>Ethical approval: yes	Uruguay<br>Lower-middle<br><br>Argentina, Paraguay, Peru<br>Upper-middle<br><br>*Also includes data from Chile*<br>*High-income*	"The purpose of this study was to assess the association between antenatal MgSO4 exposure and neonatal mortality, the incidence of severe IVH and composite outcome IVH grade III-IV/ death in VLBW infants. Secondarily, explore potential in-hospital undesired effects of MgSO4 administration in this cohort."	Cochrane review: Doyle 2009<br>RCTs: Rouse 2008<br>Other relevant SRs/guidelines: Crowther 2017, SOGC 2019, Moradi 2020, FIGO 2021, Prasath 2023, Shepherd 2019, Zeng 2016
Wang 2024 [53]	Full-text publication<br><br>*Pre-print*	English	Non-RCT Retrospective case-control study	January 2015 to June 2022	Funding: "The study was funded by Science & Technology Department of Sichuan Province [2022 NSFSC0657]"	China<br>Upper-middle	"we aimed to explore the effects of different doses of antenatal betamethasone on the hearing of preterm infants and the effects of different doses of	Cochrane review: Doyle 2009<br>RCTs: Rouse 2008

Study ID	Format of publication	Language	Design	Dates of study	Funding, Declaration, Ethics, Prospective RCT registration	Setting, World Bank Classification	Aim/purpose	MgSO4 evidence cited
					Declaration: nil<br>Ethical approval: yes		antenatal betamethasone alone or in combination with magnesium sulfate on the hearing of preterm infants”	
Wu 2022 [54]	Abstract (full-text publication not available)	English	Non-RCT<br>“Retrospective study” (unclear based on abstract, likely non-concurrent cohort study)	July 2014 to June 2021	Funding: NR<br>Declaration: NR<br>Ethical approval: NR	China<br>Upper-middle	“To analyze the outcomes of extremely preterm infants (EPIs) after the implementation and quality improvement of an intervention program from the prenatal period to delivery room and the factors influencing the mortality of EPIs.”	Nil
Xiao 2024 [55]	Full-text publication	English	Non-RCT<br>"Retrospective analysis of data from a prospective cohort" (retrospective cohort)	January 2019 to December 2021	Funding: “This work was funded by Sichuan Natural Science Foundation (2023NSFSC1604), and the Canadian Institutes of Health Research (CTP87518).”<br>Declaration: nil<br>Ethical approval: yes	China<br>Upper-middle	“We aim to investigate the characteristics of the perinatal strategies associated with sIVH for VPIs across the multiple NICUs in China.”	Other relevant SRs/guidelines: Moradi 2020
Yuce 2023 [56]	Full-text publication	English	Non-RCT<br>"patient records were evaluated retrospectively" (likely case series)	2019 to 2022	Funding: nil<br>Declaration: nil<br>Ethical approval: yes	Turkey<br>Upper-middle	“there is no data on neuroprotective MgSO4 administration on maternal APRI scores. Thus, this study	WHO [no citation]<br>Other relevant SRs/guidelines: ACOG 2016, Costantine 2009,

Study ID	Format of publication	Language	Design	Dates of study	Funding, Declaration, Ethics, Prospective RCT registration	Setting, World Bank Classification	Aim/purpose	MgSO4 evidence cited
							aimed to evaluate the changes in APRI scores in pregnant women who received MgSO4 for neuroprotective purposes.”	Galinksy 2020, Jayaram 2019
Zhou 2023 [57]	Full-text publication	English	Non-RCT "Observational" (likely prospective cohort study)	NR (children born 2004 to 2010; followed up at 10 to 16 years CA)	Funding: The National Key Research and Development Program, Grant/Award Number: 2016YFC1000400; The general program of the Natural Science Foundation of Department of Science and Technology of Sichuan Province, Grant/Award Number: 2022NSFSC0797. This study was supported by the National Key Research and Development Program “Reproductive Health and Prevention and Control of Major Birth Defects” (No.	China Upper-middle	"This study aimed to use VBM to investigate the long-term effects of MgSO4 on GM morphology in PTB children."	WHO 2015 Other relevant SRs/guidelines: ACOG 2016, NICE 2015

Study ID	Format of publication	Language	Design	Dates of study	Funding, Declaration, Ethics, Prospective RCT registration	Setting, World Bank Classification	Aim/purpose	MgSO4 evidence cited
					2016YFC1000400) and "Health Commission of Sichuan Province" (No. 17ZD007) of China."<br>Declaration: nil<br>Ethical approval: yes			

Abbreviations: ACOG: American College of Obstetricians and Gynecologists; ACTOMgSO4: Australasian Collaborative Trial of Magnesium Sulphate; BEAM: Beneficial Effects of Antenatal Magnesium Sulfate; CA; corrected age; FIGO: International Federation of Gynecology and Obstetrics; FOGSI: Federation of Obstetric and Gynaecological Societies of India; GM: gray matter; IOG: Institute of Obstetricians and Gynaecologists (Ireland); NHS: National Health Service; NICE: National Institute for Health and Care Excellence; NR: not reported; PPROM: preterm prelabour rupture of membranes; PROM: prelabour rupture of membranes; PTB: preterm born; RANZCOG: Royal Australian and New Zealand College of Obstetricians and Gynecologists; RCOG: Royal College of Obstetricians and Gynaecologists; RCT: randomised controlled trial; SOGC: Society of Obstetricians and Gynaecologists of Canada; SR: systematic review; UoA: University of Adelaide; VBM: voxel-based morphometry; VLBW: very low birthweight; VPI: very preterm infants; WHO: World Health Organization

References (Included Research Studies)

1. Abdel Fattah G. NCT02506894: Fetal Middle Cerebral Artery Doppler in Preterm Births Receiving Magnesium Sulfate for Neuroprotection 2015 [cited 4 December 2023]. Available from: https://clinicaltrials.gov/study/NCT02506894.
2. Achola KA, Lester F, Lihanda P, Olack B, Otare C, Miller L, et al. Adapting the WHO Safe Childbirth Checklist for preterm birth in Migori County, Western Kenya Int J Gynaecol Obstet. 2018;143(S3):158.
3. Atia H. NCT05674565: Magnesium Sulphate Neuroprotective Strategies for Preterm Deliveries 2023 [cited 9 November 2023]. Available from: https://clinicaltrials.gov/study/NCT05674565.
4. Bachnas MA, Sulistyowati S, Akbar U. Dose dependency in antenatal magnesium sulphate to promote brain-derived neurotrophic factor production. Ultrasound Obstet Gynecol. 2020;56(Suppl 1):147.
5. Bachnas MA, Sulistyowati S, Akbar U. Correlation between antenatal magnesium sulfate (MgSO4) total dose and delivery time interval with umbilical cord blood brain-derived neurotrophic factor (BDNF) levels as a neuroprotection strategy in preterm birth. Bali Med J. 2022;11(1):293-8.
6. Bansal V, Desai A. Efficacy of Antenatal Magnesium Sulfate for Neuroprotection in Extreme Prematurity: A Comparative Observational Study. J Obstet Gynaecol India. 2022;72(Suppl 1):36-47.
7. Cavalcanti RM, Sciamareli N, Campos F, Morato R, Souza AC, Silva R, et al. Tips and challenges to implement good childbirth care practices at an university hospital in Brazil. Int J Gynaecol Obstet. 2018;143(Suppl 3):158.
8. Chandran S, Tergestina M, Ross B, Joshi A, Rebekah G, Kumar M. Effects of Antenatal Magnesium Sulfate on the Gut Function of Preterm (<32 weeks) Very Low Birth Weight Neonates: Experience from a Tertiary Institute in South India. J Trop Pediatr. 2021;67(2):1-9.
9. Daneji SM, Kasim NI, Takai IU. Clinical audit of the management and outcomes of preterm pre-labour rupture of membranes at Aminu Kano Teaching Hospital. Pan Afr Med J. 2022;43:41.
10. Diggikar S, Kulkarni S, Aradhya AS, Venkatagiri P. Use of antenatal magnesium sulfate for neuroprotection: a survey of national practices. Int J Contemp Pediatrics. 2021;8(4):744-6.
11. Dolly M, Mammen MV. Retrospective Study of Neonatal Outcomes with the Use of Magnesium Sulfate in Case of Preterm Labor. Int J Res Publ Rev. 2022;3(10):886-94.
12. Gathwala G. CTRI/2018/06/014386: Antenatal magnesium sulphate for neuroprotection in preterm infants: An open label randomized control trial 2018 [cited 4 December 2023]. Available from: http://www.ctri.nic.in/Clinicaltrials/pmaindet2.php?trialid=22360.
13. Gupta N, Garg R, Gupta A, Mishra S. Magnesium Sulfate for Fetal Neuroprotection in Women at Risk of PretermBirth: Analysis of its Effect on Cerebral Palsy. J South Asian Fed Obstet Gynecol. 2021;13(3):90-3.
14. Gupta N, Jahan U, Seep S, Singh CP, Shukla S. Role of magnesium sulphate as a neuroprotective agent on neonatal outcome in preterm deliveries. Int J Reprod Contracept Obstet Gynecol. 2023;12(3):658-64.
15. Iqbal M, Muhammad Z, Iqbal S. Role of magnesium sulfate for fetal neuroprotection in women at risk of preterm birth. J Peoples U Med Health Sci. 2023;13(1):124-8.
16. Jamileh S, Masoumeh S, Nezamaddin E, Jalileh S, Gheshlagh Reza G, Sedigheh B, et al. Effect of magnesium sulfate on middle cerebral, umbilical artery and uterine arteries Doppler parameters in pregnancy up to 32 weeks. Med Sci. 2018;22(94):549-52.
17. Jayashree V, Vanathi R, Athmika N. Effect of magnesium sulphate for antenatal mothers in preterm labour for neuroprotection in infants. Int J Clin Obstet Gynaecol. 2021;6(6):243-9.
18. Jin D, Gu X, Jiang S, Wang Y, Yang T, Lu Y, et al. The Association between Evidence-Based Healthcare Practices and Outcomes among Preterm Births in China. Neonatology. 2022;119(1):26-32.
19. Kasapoglu I, Cetinkaya Demir B, Atalay MA, Orhan A, Ozkan H, Cakir SC, et al. Does antenatal magnesium sulphate improve hearing function in premature newborns? J Turk Ger Gynecol Assoc. 2020;21(3):187-92.
20. Kumar N, Kumar P, Kumar S, Tulsiyan P. Antenatal Low Dose Magnesium Sulphate for Foetal Neuroprotection in Preterm Birth Versus Control: A Comparative Prospective Cohort Study in Tertiary Care Centre in India. J Obstet Gynaecol India. 2025;75(1):46-52.
21. Mamatha S, Ananda M, Shaziya S, Manjunatha S, Somashekar HK. Maternal and Fetal outcome in usage of magnesium sulphate for fetal neuroprotection in pregnancy less than 34 weeks of gestation. Int J Life Sci Biotechnol Pharma Res. 2023;12(1):488-97.
22. Manoj Varanattu C, Menon B, Sankar A, Anand M, Manikoth P. Randomized controlled trial of antenatal magnesium sulfate for short-term neuroprotection in premature neonates. Indian J Child Health. 2017;4(2):199-202.
23. Marnal A, Poornima M, Suma KB, Sahana K. MgSO4 for neuroprophylaxis in preterm birth - clinical audit-re audit. Dig J Clin Med. 2023;5(4):121-3.
24. Mazhar T, Rauf S, Ambareen A, Nadir S. The efficacy and safety of magnesium sulphate for neonatal neuroprotection in patients with imminent preterm deliveries: experience at a tertiary care hospital. J Med Sci. 2023;31(2):137-42.

25. Medhi R, Das I, Boro RC, Naznin W. Fetomaternal outcome in patients with early preterm labour following administration of magnesium sulphate-a hospital based prospective study. New Indian J OBGYN. 2023;10(1):39-45.
26. Millogo-Traoré TFD, Sawadogo O, Zongo-Kondé SW. Evaluation of the Fetal Neuroprotection Protocol with Magnesium Sulphate in a University Hospital in Burkina Faso. Open J Obstet Gynecol. 2022;12:1042-55.
27. Mohan RJ, Lakshmy S, Fiji. The Effect of Antenatal Magnesium Sulphate for Fetal Neuroprotection in Threatened Preterm Labour: A Prospective Cohort Study. Int J Innov Sci Res Technol. 2023;8(9):837-93.
28. Muhammed HO, Murad SJ. Effect of Prenatal Infusion of Magnesium Sulphate on Neurological Complication in Preterm Infant. Int J Pharm Res. 2019;11(3):29-34.
29. Musiime G, Lloyd L, McCaul M, Van Zyl N, Holgate S. Outcomes of extremely low-birthweight neonates at a tertiary hospital in the Western Cape, South Africa: A retrospective cohort study. S Afr J Child Health. 2021;15(3):170-5.
30. Nunes RD, Schutz FD, Traebert JL. Association between the use of magnesium sulfate as neuroprotector in prematurity and the neonatal hemodynamic effects. J Matern Fetal Neonatal Med. 2018;31(14):1900-5.
31. Okulu E, Kraja E, Kostekci YE, Seker E, Ozisik MS, Sarisoy D, et al. Effect of Antenatal Magnesium Sulfate Exposure on Patent Ductus Arteriosus in Premature Infants. Am J Perinatol. 2024;41(12):1665-72.
32. Ozer Bekmez B, Oguz Y, Kutman HGK, Uygur D, Canpolat FE, Oguz SS, et al. The Effect of Antenatal Neuroprotective Magnesium Sulfate Treatment on Cerebral Oxygenation in Preterm Infants. Am J Perinatol. 2021;38(S 01):e64-e70.
33. Ozer Bekmez B, Kanmaz Kutman HG, Oguz Y, Uygur D, Elbayiyev S, Canpolat FE, et al. Antenatal Neuroprotective Magnesium Sulfate in Very Preterm Infants and Its Association With Feeding Intolerance. J Pediatr Gastroenterol Nutr. 2023;77(5):597-602.
34. Özlü F, Hacıoğlu C, Büyükkurt S, Yapıcıoğlu H, Satar M. Changes on preterm morbidities with antenatal magnesium. Çukurova Med J. 2019;44(2):502-8.
35. Padmapriya R, Niveditha L, Sukanya L, Saraswathi N. Role of antenatal magnesium sulphate as a fetal neuroprotection in preterm labour. Int J Acad Med Pharm. 2023;5(4):699-703.
36. Pandey D, Arja K, Inukollu PR, Kuppili M. The role of magnesium sulphate (MgSO4) in fetal neuroprotection. J Cardiovasc Dis Res. 2024;15(8):122-8.
37. Parashi S, Bordbar A, Mahmoodi Y, Jafari MR. The survey of magnesium sulfate in prevention of intraventricular hemorrhage in premature infants: a randomized clinical trial. Shiraz E Med J. 2017;18(11):e55094.
38. Patel D, Vora A, Goyal P, Dubey P. Effects of Antenatal Steroids with Magnesium Sulphate on Intraventricular Hemorrhage and Periventricular Leukomalacia in Neonates Born below 32 Weeks of Gestation. Nat J Med Res. 2024;14(1):4-10.
39. Peng S, He X, Xia S. Extremely preterm infants born outside a provincial tertiary perinatal center and transferred postnatally associated with poor outcomes: a real-world observational study. Front Pediatr. 2024;12:1287232.
40. Phrasidthideth B, Phengsavanh A, Kiatchoosakun P. Evaluation of preterm and low birth weight morbidity, mortality and standards of care in Lao PDR. Lao Med J. 2021:31-40.
41. Pirjani R, Zndi S. IRCT20120826010664N5: Evaluation of neuroprotective effects of magnesium sulfate on preterm infants with a gestational age of 32-36 weeks 2019 [cited 6 December 2023]. Available from: http://en.irct.ir/trial/41934.
42. Rauf M, Sevil E, Ebru C, Yavuz S, Cemil C. Antenatal magnesium sulfate use for fetal neuroprotection: experience from a tertiary care hospital in Turkey. Biomed Res - India 2017;28(4):1749-54.
43. Rivera-Rueda M, Fernández-Carrocera L, Michel-Macías C, Carrera-Muiños S, Arroyo-Cabrales L, Coronado-Zarco I, et al. [Morbilidad y mortalidad de neonatos< 1,500 g ingresados a la UCIN de un hospital de tercer nivel de atención]. Perinatol Reprod Hum. 2017;31(4):163-9.
44. Sarath MB, Anand MR, Remesh P, Nath D, Mohan V. Postnatal Magnesium Trend in less than 34 week Preterms who received Antenatal Neuroprotective Doses of Magnesium Sulphate. Fetus Newborn. 2021;1(2):34–7.
45. Sariati Y, Nooryanto M, Anggraini PDA. Pengaruh Penggunaan Magnesium Sulfate (MgSO4) Saat Antenatal Sebagai Neuroprotektor Bayi Prematur Terhadap Perkembangan Motorik Kasar Usia 2-3 Tahun. J Issues Midwifery. 2017;1(2):50-7.
46. Sharma K, Ranideepa, Anamika. To study the effect of antenatal magnesium sulphate for neuroprotection in preterm babies. Int J Adv Res. 2021;9(4):628-33.
47. Sheeba LM, Namboodiripad A, Varanattu MC. Efficacy of Maternal Magnesium Sulfate Administration on the Neurodevelopmental Outcome of Preterm Babies: A Randomised Controlled Trial. J Clin Diagn Res. 2022;16(12):SC06-SC9.
48. Sulistyowati S, Bachnas MA, Ekasari S, Wijayanti AS. Correlation of the Magnesium Serum Levels in Maternal and Fetal over the Fetal Brain-Derived Neurotrophic Factor (BDNF) after Antenatal Magnesium

Sulphate (MgSO4) Provision in the Preterm Birth Neuroprotection Strategy. Syst Rev Pharm. 2021;12(1):1624-9.
49. Tom SM, Manoj VC. Effect of antenatal administration of magnesium sulphate and milking of umbilical cord during delivery on the incidence of intraventricular haemorrhage in preterm infants. Int J Contemp Pediatrics. 2018;5(5):1943-6.
50. Tummala M, Upadhya R, Pai MV. Timing of administration of antenatal magnesium sulphate and correlation with umbilical cord blood magnesium levels in preterm babies. Eur J Obstet Gynecol Reprod Biol. 2024;297:197-201.
51. Üstün N, Hocaoğlu M, Turgut A, Ovalı F. Effects of antenatal magnesium sulfate use for neuroprotection on cardiorespiratory complications during the early neonatal period in preterm infants. J Surg Med. 2021;5(9):843-7.
52. Vaz Ferreira C, Caro J, Villarroel L, Munoz S, Alvarez P, Flores G, et al. Antenatal exposure to magnesium sulfate and neonatal outcomes in very low birth weight infants: a multicenter study. J Perinatol. 2024;44(11):1663-8.
53. Wang Y, Chang J, Huang Y. Effects of different doses of antenatal betamethasone alone or in combination with magnesium sulfate exposure on hearing in preterm infants: A retrospective case–control study. Research Square [Preprint]. https://doi.org/10.21203/rs.3.rs-4397672/v1]. 2024 [updated 21 May 2024 cited 4 March 2025]. Available from: https://www.researchsquare.com/article/rs-4397672/v1.
54. Wu Y, Zhong X, Zhou L, Gong H. Effects of intervention program from prenatal period to delivery room on outcomes of extremely preterm infants. Chin J Perinat Med. 2022;4:263-70.
55. Xiao T, Hu L, Chen H, Gu X, Zhou J, Zhu Y, et al. The performance of the practices associated with the occurrence of severe intraventricular hemorrhage in the very premature infants: data analysis from the Chinese neonatal network. BMC Pediatr. 2024;24(1):394.
56. Yüce E. Does MgSO4 Treatment Affect Maternal Aspartate Aminotransferase to Platelet Ratio Index (APRI) Score in Preterm Labor? Med Rec. 2023;5(3):644-7.
57. Zhou L, Liu X, Yan X, Liu Y, Xie Y, Sun C. Long-term effects of prenatal magnesium sulfate exposure on nervous system development in preterm-born children. Food Sci Nutr. 2023;11(11):7061-9.
58. Niveditha M. Role of Antenatal Magnesium Sulphate as a Fetal Neuroprotection in Preterm Babies [MS (Obstetrics and Gyanecology) thesis]: Coimbatore Medical College, Coimbatore; 2020.

References (Supporting Evidence Cited)

Guidelines

ACOG 2010:
American College of Obstetricians and Gynecologists. Committee Opinion No. 455: Magnesium sulfate before anticipated preterm birth for neuroprotection. Obstet Gynecol. 2010;115(3):669-71.

ACOG 2013:
American College of Obstetricians and Gynecologists. Committee Opinion No. 573: Magnesium sulfate use in obstetrics. Obstet Gynecol. 2013;122(3):727-8.

ACOG 2016:
American College of Obstetricians and Gynecologists. Committee Opinion No 652: Magnesium Sulfate Use in Obstetrics. Obstet Gynecol. 2016;127(1):e52-e3.

ACOG 2020:
American College of Obstetricians and Gynecologists. Prelabor Rupture of Membranes: ACOG Practice Bulletin, Number 217. Obstet Gynecol. 2020;135(3):e80-e97.

France 2017:
Sentilhes L, Senat MV, Ancel PY, Azria E, Benoist G, Blanc J, et al. Prevention of spontaneous preterm birth: Guidelines for clinical practice from the French College of Gynaecologists and Obstetricians (CNGOF). Eur J Obstet Gynecol Reprod Biol. 2017;210:217-24.

FIGO 2021:
Shennan A, Suff N, Jacobsson B, Simpson JL, Norman J, Grobman WA, et al. FIGO good practice recommendations on magnesium sulfate administration for preterm fetal neuroprotection. Int J Gynecol Obstet. 2021;155(1):31-3.

FOGSI 2017:

Federation of Obstetric and Gynaecological Societies of India (FOGSI). FOGSI Focus: Prevention of pre-term labour 2017 [cited 8 December 2023]. Available from: https://www.fogsi.org/wp-content/uploads/fogsi-focus/fogsi-focus-ptl.pdf.

IOG 2013:
Institute of Obstetricians and Gynaecologists (IOG) - Royal College of Physicians of Ireland And Directorate of Strategy and Clinical Care Health Service Executive. Clinical practice guideline: Antenatal magnesium sulphate for fetal neuroprotection. 2013.

NHS 2019:
Bisht J, Impany L, Ablett J, Philip S. Magnesium Sulphate for neuroprotection (GL868). Magsul guidelines V3 April 2019. Oxford AHSN. Regional maternity guidelines. NHS Foundation Trust. Royal Berkshire.; 2019.

NHS 2020:
NHS - Foundation Trust. MgSO4 for neonatal neuroprotection. 2020.

NICE 2015:
National Institute for Health and Care Excellence (NICE). Preterm labour and birth. NICE guideline NG25. 2015.

RCOG 2011:
Peebles D, Kenyon A. Magnesium sulphate to prevent cerebral palsy following preterm birth. RCOG Scientific Impact Paper 29, 2011. 2011.

RCOG 2019:
Thomson AJ, Royal College of Obstetricians and Gynaecologists. Care of Women Presenting with Suspected Preterm Prelabour Rupture of Membranes from 24(+0) Weeks of Gestation: Green-top Guideline No. 73. BJOG. 2019;126(9):e152-e66.

SOGC 2011:
Magee L, Sawchuck D, Synnes A, von Dadelszen P, Magnesium Sulphate For Fetal Neuroprotection Consensus C, Maternal Fetal Medicine C. SOGC Clinical Practice Guideline. Magnesium sulphate for fetal neuroprotection. J Obstet Gynaecol Can. 2011;33(5):516-29.

SOGC 2019:
Magee LA, De Silva DA, Sawchuck D, Synnes A, von Dadelszen P. No. 376-Magnesium Sulphate for Fetal Neuroprotection. J Obstet Gynaecol Can. 2019;41(4):505-22.

South Australia:
Department for Health and Wellbeing GoSA. South Australian Perinatal Practice Guideline: Magnesium Sulphate for Neuroprotection of the Fetus in Women at Risk of Preterm Birth [Available from: https://www.sahealth.sa.gov.au/wps/wcm/connect/86f3f2804ee4f45294189dd150ce4f37/Magnesium+Sulphate+for+Neuroprotection+of+the+Fetus_PPG_v4_1.pdf?MOD=AJPERES&CACHEID=ROOTWORKSPACE-86f3f2804ee4f45294189dd150ce4f37-p4bHW55.

UoA 2010:
Antenatal Magnesium Sulphate for Neuroprotection Guideline Development Panel. Antenatal Magnesium Sulphate Prior to Preterm Birth for Neuroprotection of the Fetus, Infant, and Child 2010: National Clinical Practice Guidelines. Adelaide: Australian Research Centre for Health of Women and Babies, the University of Adelaide; 2010.

WHO 2015:
World Health Organisation. WHO recommendations on interventions to improve preterm birth outcomes 2015 [cited 30 March 2023]. Available from: https://www.who.int/publications/i/item/9789241508988.

WHO 2015b:
World Health Organization. WHO recommendation on the use of magnesium sulfate for fetal protection from neurological complications. World Health Organization. 2015.

Walker S. Magnesium Sulphate in Women at risk of preterm birth for neuroprotection of the fetus [RANZCOG]. O&G Mag. 2010;12:38-41.

Systematic reviews

Bain 2012:

Bain E, Middleton P, Crowther CA. Different magnesium sulphate regimens for neuroprotection of the fetus for women at risk of preterm birth. Cochrane Database Syst Rev. 2012;2012(2):CD009302.

Bain 2013:
Bain ES, Middleton PF, Crowther CA. Maternal adverse effects of different antenatal magnesium sulphate regimens for improving maternal and infant outcomes: a systematic review. BMC Preg Childbirth. 2013;13:195.

Conde-Agudelo 2009:
Conde-Agudelo A, Romero R. Antenatal magnesium sulfate for the prevention of cerebral palsy in preterm infants less than 34 weeks' gestation: a systematic review and metaanalysis. Am J Obstet Gynecol. 2009;200(6):595-609.

Costantine 2009
Costantine MM, Weiner SJ. Effects of antenatal exposure to magnesium sulfate on neuroprotection and mortality in preterm infants: a meta-analysis. Obstet Gynecol. 2009;114(2 Pt 1):354-64.

Crowther 2017:
Crowther CA, Middleton PF, Voysey M, Askie L, Duley L, Pryde PG, et al. Assessing the neuroprotective benefits for babies of antenatal magnesium sulphate: An individual participant data meta-analysis. PLoS Med. 2017;14(10):e1002398.

Doyle 2009:
Doyle LW, Crowther CA, Middleton P, Marret S, Rouse D. Magnesium sulphate for women at risk of preterm birth for neuroprotection of the fetus. Cochrane Database Syst Rev. 2009(1):CD004661.

Galinsky 2020:
Galinsky R, Dean JM, Lingam I, Robertson NJ, Mallard C, Bennet L, et al. A Systematic Review of Magnesium Sulfate for Perinatal Neuroprotection: What Have We Learnt From the Past Decade? Front Neurol. 2020;11:449.

Jayaram 2019:
Jayaram PM, Mohan MK, Farid I, Lindow S. Antenatal magnesium sulfate for fetal neuroprotection: a critical appraisal and systematic review of clinical practice guidelines. J Perinat Med. 2019;47(3):262-9.

Moradi 2020:
Moradi Y, Khateri R, Haghighi L, Dehghani S, Hanis SM, Valipour M, et al. The effect of antenatal magnesium sulfate on intraventricular hemorrhage in premature infants: a systematic review and meta-analysis. Obstet Gynecol Sci. 2020;63(4):395-406.

Prasath 2023:
Prasath A, Aronoff N, Chandrasekharan P, Diggikar S. Antenatal Magnesium Sulfate and adverse gastrointestinal outcomes in Preterm infants-a systematic review and meta-analysis. J Perinatol. 2023;43(9):1087-100.

Shepherd 2019:
Shepherd E, Salam RA, Manhas D, Synnes A, Middleton P, Makrides M, et al. Antenatal magnesium sulphate and adverse neonatal outcomes: A systematic review and meta-analysis. PLoS Med. 2019;16(12):e1002988.

Walker 2010:
Walker S. Magnesium Sulphate in Women at risk of preterm birth for neuroprotection of the fetus [RANZCOG]. O&G Mag. 2010;12:38-41.

Zeng 2016:
Zeng X, Xue Y, Tian Q, Sun R, An R. Effects and safety of magnesium sulfate on neuroprotection: A meta-analysis based on PRISMA guidelines. Medicine (Baltimore). 2016;95(1):e2451.

RCTs

Altman 2002:
Altman D, Carroli G, Duley L, Farrell B, Moodley J, Neilson J, et al. Do women with pre-eclampsia, and their babies, benefit from magnesium sulphate? The Magpie Trial: a randomised placebo-controlled trial. Lancet. 2002;359(9321):1877-90.

Crowther 2003 [ACTOMgSO4]:
Crowther CA, Hiller JE, Doyle LW, Haslam RR. Effect of magnesium sulfate given for neuroprotection before preterm birth: a randomized controlled trial. JAMA. 2003;290(20):2669-76.

Crowther 2013:

Crowther CA, Middleton PF, Wilkinson D, Ashwood P, Haslam R, the MSG. Magnesium sulphate at 30 to 34 weeks' gestational age: neuroprotection trial (MAGENTA) - study protocol. BMC Preg Childbirth. 2013;13(1):91.

Crowther 2023 [MAGENTA]:
Crowther CA, Ashwood P, Middleton PF, McPhee A, Tran T, Harding JE, et al. Prenatal Intravenous Magnesium at 30-34 Weeks' Gestation and Neurodevelopmental Outcomes in Offspring: The MAGENTA Randomized Clinical Trial. JAMA. 2023;330(7):603-14.

Doyle 2014:
Doyle LW, Anderson PJ, Haslam R, Lee KJ, Crowther C, Australasian Collaborative Trial of Magnesium Sulphate Study G. School-age outcomes of very preterm infants after antenatal treatment with magnesium sulfate vs placebo. JAMA. 2014;312(11):1105-13.

Hirtz 2015:
Hirtz DG, Weiner SJ, Bulas D, DiPietro M, Seibert J, Rouse DJ, et al. Antenatal Magnesium and Cerebral Palsy in Preterm Infants. J Pediatr. 2015;167(4):834-9 e3.

Magpie 2007:
Magpie Trial Follow-Up Study Collaborative G. The Magpie Trial: a randomised trial comparing magnesium sulphate with placebo for pre-eclampsia. Outcome for children at 18 months. BJOG. 2007;114(3):289-99.

Marret 2007 [PREMAG]:
Marret S, Marpeau L, Zupan-Simunek V, Eurin D, Leveque C, Hellot MF, et al. Magnesium sulphate given before very-preterm birth to protect infant brain: the randomised controlled PREMAG trial*. BJOG. 2007;114(3):310-8.

Marret 2008 [PREMAG]:
Marret S, Marpeau L, Follet-Bouhamed C, Cambonie G, Astruc D, Delaporte B, et al. [Effect of magnesium sulphate on mortality and neurologic morbidity of the very-preterm newborn (of less than 33 weeks) with two-year neurological outcome: results of the prospective PREMAG trial]. Gynecol Obstet Fertil. 2008;36(3):278-88.

Marret 2015:
Marret S, Benichou J. Antenatal magnesium sulfate and outcomes for school-aged children. JAMA. 2015;313(3):306.

Mittendorf 2002 [MagNET]:
Mittendorf R, Dambrosia J, Pryde PG, Lee KS, Gianopoulos JG, Besinger RE, et al. Association between the use of antenatal magnesium sulfate in preterm labor and adverse health outcomes in infants. Am J Obstet Gynecol. 2002;186(6):1111-8.

Rouse 2008 [BEAM]:
Rouse DJ, Hirtz DG, Thom E, Varner MW, Spong CY, Mercer BM, et al. A randomized, controlled trial of magnesium sulfate for the prevention of cerebral palsy. N Engl J Med. 2008;359(9):895-905.

Rouse 2009 [BEAM]:
Rouse DJ, Hirtz DG, Thom EA. Magnesium Sulfate for the Prevention of Cerebral Palsy Reply. N Engl J Med. 2009;360(2):190-.

Wolf 2020 [MASP]:
Wolf HT, Brok J, Henriksen TB, Greisen G, Salvig JD, Pryds O, et al. Antenatal magnesium sulphate for the prevention of cerebral palsy in infants born preterm: a double-blind, randomised, placebo-controlled, multi-centre trial. BJOG 2020;127(10):1217-25.

Other

Costantine 2011:
Costantine MM, Drever N. Antenatal exposure to magnesium sulfate and neuroprotection in preterm infants. Obstet Gynecol Clin North Am. 2011;38(2):351-66, xi.

Mittendorf 2009:
Mittendorf R, Pryde PG. Magnesium sulfate for the prevention of cerebral palsy. N Engl J Med. 2009;360(2):189-90; author reply 90.

S4 Table. Characteristics of research studies (2)

Study ID	Population characteristics	MgSO4 intervention/exposure	Non MgSO4 comparator	Outcome(s) specified/reported in relation to MgSO4*	Main results relevant to MgSO4	Main conclusion relevant to MgSO4
Abdel Fattah 2015 [1]	Inclusion: pregnant women with preterm labour; < 32 weeks GA, singleton pregnancy, aged 20-40 years<br>Exclusion: contraindication or hypersensitivity to magnesium, pre-eclampsia, multiple pregnancy, intake of MgSO4 in this pregnancy<br>N = 60 women	6 g IV LD over 20 min followed by 1 g/hour IV MD for 24 hours<br><br>*IV assumed*	Placebo (sodium chloride solution over 24 hours IV)	Pre-specified primary outcome(s): changes in fetal middle cerebral artery Doppler indices (peak systolic velocity, pulsatility index, resistance index) (3 months)<br>Pre-specified secondary outcome(s): maternal adverse effects of MgSO4 (3 months)	NA	NA
Achola 2018 [2]	Inclusion: preterm births during the study period<br>N = 353 babies (77 [21%] < 34 weeks)	MgSO4 (N = 7 babies)	No MgSO4 (N = 70 babies)	Reported outcome(s)*: received MgSO4	"9% (7/77) [received] magnesium sulphate."	"Results reveal modest use of evidence-based interventions for preterm babies."
Atia 2023 [3]	Inclusion: women at risk of preterm birth between 24+0 and 33+6 weeks GA, when early preterm birth is planned or expected within 24 hours, regardless of: plurality or parity, reason for risks of preterm birth, anticipated mode of birth, whether antenatal corticosteroids have been given<br>Exclusion: women with known hypersensitivity to magnesium, caution regarding dosage for patients with renal impairment, preterm delivery after 34 weeks<br>N = 336 women	LD only: 4 g IV LD over 20 min within 1 hour before delivery<br><br>LD plus MD: 4 g IV LD over 20 min, followed by 1 g/hour IV MD until delivery or completion of 24 hours, whichever is sooner	No MgSO4	Pre-specified primary outcome(s): neurological insults during the first year of life (including cerebral palsy, brain leukomalacia, IVH, and neonatal seizures), maternal MgSO4 toxicity up to 12 hours after end of therapy (affected reflexes, respiratory and cardiac), postpartum haemorrhage, primary postpartum haemorrhage<br>Pre-specified secondary outcome(s): late appearing neurological insult (24 months after birth): risk of gross motor delay, epilepsy, impaired fine motor skills,	NA	NA

Study ID	Population characteristics	MgSO4 intervention/exposure	Non MgSO4 comparator	Outcome(s) specified/reported in relation to MgSO4*	Main results relevant to MgSO4	Main conclusion relevant to MgSO4
				sensorineural (hearing and vision) impairment, and possibly 2 years of age developmental quotient; death within first 28 days		
Bachnas 2020 [4]	Inclusion: "subjects of inevitable preterm labour in the same gestational age"<br>N = 75 women	LD only: 4 g IV bolus LD (N = NR)<br><br>LD plus MD: 4 g IV bolus LD followed by 1 g/hour MD IV (N = NR)<br><br>*IV assumed*	Nil	Pre-specified outcome(s): BDNF production (cord blood)	"antenatal magnesium sulphate dose only showed weak correlation to promote BDNF production (r2=0.254; p=0.028)."	"Giving 4 grams bolus only in consideration to promote BDNF for neuroprotection purposes has comparable efficacy as continued by 1g/ hour maintenance."
Bachnas 2022 [5]	Inclusion: pregnant women, 20-35 years of age, 28-34 weeks GA in an active phase of labour or planned for delivery due to medical reasons, agreed to participate<br>Exclusion: multiple pregnancies, intrauterine fetal death, a fetus with congenital abnormalities, born 3 hours after treatment started or more than 48 hours, and a prior history of MgSO4 adverse events<br>N = 81 women enrolled: 72 in final analysis	LD only: 4 g IV LD (N = 20 women)<br><br>Low dose LD plus MD: 4 g IV LD followed by 1 g/hour IV MD for 24 hours (N = 36 women)<br><br>High dose LD plus MD: 6 g IV LD followed by 2 g/hour IV MD for 24 hours (N = 16 women)	Nil	Pre-specified primary outcome(s): correlation between antenatal MgSO4 total dose and umbilical cord blood BDNF levels<br>Pre-specified secondary outcome(s): correlations between delivery time interval and umbilical cord blood BDNF levels<br>Other pre-specified outcome(s): association between the doses of antenatal corticosteroid (dexamethasone) used with the umbilical cord BDNF levels<br>Other reported outcome(s): factors affecting BDNF levels (maternal magnesium, umbilical cord blood magnesium), correlation of	"The total dose of MgSO4 showed a significant weak effect to increase umbilical cord blood BDNF levels (r=0.381, p<0.001). There was no significant correlation between delivery time interval and umbilical cord blood BDNF levels (r=0.092, p=0.440)."	"All protocols exist in guidelines worldwide of antenatal MgSO4 provision… gives almost comparable result on BDNF production. But as the world has already been familiar with 4 g initial continued with 1g/h maintenance, which is used in pre-eclampsia management protocols. It is probably the best choice for daily practice nowadays. However, 4 g only and 6 g followed with 2 g/h could still also be chosen, considering the administration easiness of the 4 g only, and the

Study ID	Population characteristics	MgSO4 intervention/exposure	Non MgSO4 comparator	Outcome(s) specified/reported in relation to MgSO4*	Main results relevant to MgSO4	Main conclusion relevant to MgSO4
				total MgSO4 with maternal and fetal magnesium levels		potential of a little bit higher fetal BDNF result in 6 g followed with 2g/h, resulting from higher MgSO4 total dose."
Bansal 2022 [6]	Inclusion: women who gave birth between 26-34 weeks GA due to spontaneous preterm labour and/or planned birth for fetal or maternal indications<br>Exclusion: women who delivered before they could get the benefit of 2 doses of steroids for lung maturity, multiple pregnancies and those with a major fetal abnormality<br>N = 100 women	4 g IV LD over 30 min, preferably 4 hours prior to birth (N = 50 women/infants)	No MgSO4 (N = 50 women/infants)	Reported outcome(s): mode of birth (caesarean section), maternal side effects: nausea, dizziness, flushing, tachycardia, requirement for medication or cessation of therapy, postpartum haemorrhage, massive postpartum haemorrhage, maternal mortality; birthweight, 5 min Apgar score < 5, < 7 and average, methods of resuscitation (not required, oxygen by hood, CPAP, intubation), NICU admission, death before discharge, number of days in NICU, respiratory support including mechanical ventilation of day 1, requirement for ongoing respiratory support (e.g., for > 10 days, > 25 days), cranial ultrasound within first 14 days: PVL, IVH (and grade 1-4), neonatal signs and symptoms (weak cry, tachypnoea, chest retraction, hypoglycaemia, hypothermia, hypocalcaemia,	"There was no significant difference in terms of maternal mortality or serious morbidity including postpartum hemorrhage, caesarian section rates or length of hospital stay among women receiving MgSO4 versus no MgSO4. Mild maternal side effects secondary to magnesium sulfate were experienced in 8% cases. There were no significant differences between both groups for low 5 min APGAR, need for NICU admission, neonatal convulsions, hyperbilirubinemia, necrotizing enterocolitis, periventricular leukomalacia and septicemia. There was a trend toward reduced risk in the magnesium sulfate group for need for mechanical ventilation and ongoing respiratory support, intraventricular	"MgSO4 is a safe drug to use in antenatal women at risk for impending preterm. Antenatal magnesium sulfate given to women in established preterm labor conferred significant neuroprotective advantage to the neonate. MgSO4 also has protective effect on the need of invasive ventilatory support in preterm infants. Given the breadth of evidence in its favor, it is time for us to start using MgSO4 in clinical practice for neuroprotective intent in all our extreme preterm births."

Study ID	Population characteristics	MgSO4 intervention/exposure	Non MgSO4 comparator	Outcome(s) specified/reported in relation to MgSO4*	Main results relevant to MgSO4	Main conclusion relevant to MgSO4
				lethargy, hypotension, bradycardia, apnoea, seizure, weak suck, nasal flaring, sepsis), neonatal morbidity (neonatal jaundice and need for phototherapy, septicaemia, seizures, NEC)	hemorrhage, neonatal hypotension, hypothermia, length of NICU stay. IVH was less frequent and less severe in babies exposed to antenatal MgSO4 (8%) as compared to non-MgSO4 group (16%). Neonatal morbidities were more when antenatal MgSO4 was given less than 4 h from delivery."	
Cavalcanti 2018 [7]	Inclusion: women who gave birth from March to December<br>N = 587 women	"magnesium sulfate administration in preterm births" (N = NR, 100% in preterm births)	NA	Reported outcome(s)*: received MgSO4	"In March… 100%... magnesium sulfate administration in preterm births… The goal of 100%... for the clinical indicators was sustained."	"Our work shows that despite an unfavorable setting, continuing medical education is the key for improving our practices."
Chandran 2021 [8]	Inclusion: preterm (< 32 weeks GA) or VLBW (< 1500 g) inborn neonates<br>Exclusion: neonates with major congenital anomalies, severe growth restriction (< 3rd centile) and perinatal asphyxia (Apgar < 3 at 5 min) were excluded<br>N = 84 neonates	Protocol used in the department was the same for preterm neuroprotection and maternal severe pre-eclampsia: 4 g IV LD over 30 min followed by 1 g/hour IV MD until delivery (max 24 hours); repeat doses were not administered unless maternally indicated (N = 56 neonates)<br><br>*Use was for fetal neuroprotection (N =*	No MgSO4 (due to inadequate time for administration or selective decision by Department of Obstetrics – antenatal MgSO4 was not a uniform policy initially; there was a change midway through the study which made treatment mandatory < 32 weeks GA) (N = 28 neonates)	Pre-specified primary outcome(s): time to reach full feeds (time to reach 150 mL/kg/day enteral feeds)<br>Pre-specified secondary outcome(s): feed intolerance (either of the 3: increase in abdominal girth by 2 cm; pre-feed gastric residuals > 50% of cumulative feeds and bilious vomiting or aspirates), NEC, hsPDA<br>Other pre-specified outcome(s): significant hypermagnesemia (serum magnesium level > 4.5 mg/dL) (cord magnesium,	"The mean time to reach full feeds was the same in both groups (10.5 days). Feed intolerance episodes were similar in the first week of life between the exposed and unexposed groups (48.2% vs. 46.4%; p.0.88). Univariate analysis revealed no difference between groups concerning rates of NEC (p.0.17) or mortality (p.0.39). There was no significant difference in SMA Doppler parameters and	"Our study found no significant impact on postnatal feed tolerance and mesenteric blood flow among preterm VLBW neonates with antenatal MgSO4 exposure… This study reiterates that the recommended dose of Mg in the widely used antenatal MgSO4 protocol is safe in preterm neonates."

Study ID	Population characteristics	MgSO4 intervention/exposure	Non MgSO4 comparator	Outcome(s) specified/reported in relation to MgSO4*	Main results relevant to MgSO4	Main conclusion relevant to MgSO4
		30 neonates) OR pre-eclampsia (N = 26 neonates)		24-28 hour serum magnesium, and day 4-5 magnesium)<br>Other reported outcome(s): time of initiation of feeds, RDS, apnoea of prematurity, chronic oxygen dependency, inotrope requirement, sepsis, mortality, SMA Doppler velocity parameters, change in velocities from days 1-2 to 4-5	hypermagnesemia between the two groups."	
Daneji 2022 [9]	Inclusion: pregnant women with spontaneous rupture of membrane between 28 and 36 weeks 6 days GA before onset of labour<br>Exclusion: women with ruptured membranes but with the additional complications: antepartum haemorrhage, severe pre-eclampsia/eclampsia, multiple gestation, intrauterine fetal death, fetal congenital anomaly, or admission in established labour<br>N = 60 women (65% of the 92 cases of PPROM whose case notes were retrieved), assume N = 14 were < 32 weeks GA	Audit standard/clinical practice guideline for the management of PPROM included: for women with PPROM at 28-34 weeks GA and in established labour/having planned birth within 24 hours: IV MgSO4 for neuroprotection and tocolysis "to allow some time for corticosteroids to act" (N = 12 women)<br><br>*Use for fetal neuroprotection or tocolysis*	No MgSO4 (N = 2 women)	Pre-specified outcome(s)*: received MgSO4 (in women with GA ≤ 32 weeks)	"12 patients (85.7%) of those with gestational age of less than 32 weeks received MgSO4."	"Conclusion: despite high fidelity to institutional clinical practice guidelines in Aminu Kano Teaching Hospital there seems to be poor maternal and neonatal outcome with high perinatal mortality."
Diggikar 2021 [10]	Inclusion: all potential participating centres of level 2 and 3 NICUs in hospitals across India (including public and private sector hospitals);	"The most common regimen used was as per recommendation from FOGSI5 in 80 (91%) centres"	No MgSO4	Reported outcome(s): average uptake of MgSO4, written policy to manage babies < 32 weeks, aware of benefit of MgSO4, regimen	"uptake of antenatal magnesium sulfate for babies <32 weeks is about 75.2% (67/89) of centres in India, more in private	"Our survey showed that despite strong recommendations based on moderate-quality evidence, the use of

Study ID	Population characteristics	MgSO4 intervention/exposure	Non MgSO4 comparator	Outcome(s) specified/reported in relation to MgSO4*	Main results relevant to MgSO4	Main conclusion relevant to MgSO4
	invitation sent to lead clinicians (Neonatologist/Paediatrician, Obstetrician)<br>N = 100 neonatal units contacted (89 responded)			used for MgSO4, complications (hypotension), serious incident while administering the medication (mortality – assumed maternal), hurdles (knowledge, attitude, practice) in implementing a standard protocol for MgSO4, adequate facilities and monitoring to administer MgSO4, knowledge of WHO endorsing MgSO4 for neuroprotection	sector hospitals 53/66 (80.3%) compared to public sector hospitals 14/23 (60.8%). A written policy for managing babies <32 weeks is available in 62.9% of units. Adequate facilities were available in 94.3% of units to administer magnesium sulfate."	antenatal magnesium is still not widely practised in India. There is an urgent need for national guidelines and quality improvement strategies for better clinical translation of the evidence on magnesium sulfate for fetal neuroprotection."
Dolly 2022 [11]	Inclusion: all women aged 20-30 years, who came to the hospital outpatient department or emergency that were in labour < 34 weeks GA<br>Exclusion: women above 35 weeks GA, whose data were not clear<br>N = 63 women	MgSO4<br><br>"Physicians opted to administer magnesium sulfate for neuroprotection in accordance with the larger randomized trial" (N = 63 women)<br><br>*Indication unclear (fetal neuroprotection versus tocolysis): "Our study aimed in assessing magnesium sulfate as a tocolytic agent which helps in preventing cerebral palsy and other neonatal birth deficits in case of preterm births and also to*	Nil	Pre-specified outcome(s): birth weight, NICU admission - days of admission, Apgar score, complications<br>Other reported outcome(s): maternal side effects: hypotension, diarrhoea, seizure, confusion, muscle weakness, tachycardia, hypoglycaemia; neonatal outcomes: birthweight, NICU admission, intrauterine death, stillbirth, days of admission, Apgar score < 7 at 1 and 5 min, other neonatal complications: none, RDS, neonatal death, hypoglycaemia, hypotonia, hyperbilirubinaemia, jaundice, intrauterine growth	"Out of 565 pregnancies, 75 were found to be preterm labor cases in which 63 patients were given magnesium sulfate making an incidence of 11.15% as preterm labor and preterm birth."<br><br>"Observed side effects in the study population with the use of magnesium sulphate were Hypotension, Diarrhea, Seizure, Confusion, Muscle Weakness, Tachycardia, and Hypoglycemia where 47.5% was associated with Hypotension."	"The neonatal outcomes with the use of magnesium sulphate were found and shown the morbidity, mortality or complications. Early Admission in NICU promoted to prevent neonatal morbidity and mortality."

Study ID	Population characteristics	MgSO4 intervention/exposure	Non MgSO4 comparator	Outcome(s) specified/reported in relation to MgSO4*	Main results relevant to MgSO4	Main conclusion relevant to MgSO4
		assess comorbidities occurred in neonates with the maternal use of the drug during preterm labor in the area of study"… "Majority of use of magnesium sulphate as observed in patents admitted with the risk factor of pre eclampsia"		restriction, hypocalcaemia, sepsis		
Gathwala 2018 [12]	Inclusion: women aged 19-35 years, at ≤ 32 weeks GA, singleton or multiple pregnancy at risk for preterm delivery within next 24 hours; active preterm labour with cervix 4-8 cm dilated or PPROM or indicated preterm birth within next 24 hours<br>Exclusion: maternal contraindications to MgSO4 (renal failure, cardiac arrhythmia, myasthenia gravis, hypersensitivity to drug, heart block, diabetic coma or electrolyte disorder), pre-eclampsia or eclampsia, fetal distress, abruptio placenta, severe antepartum haemorrhage where urgent delivery is indicated; precipitate labour, chorioamnionitis, major fetal abnormalities, intrauterine fetal death<br>N = 30 women	4 g IV bolus LD over 20 min followed by 1 g/hour IV MD until birth or up to 12 hours whichever is earlier	Standard of care (no MgSO4)	Pre-specified primary outcome(s): combined outcome of mortality or abnormal neurodevelopmental outcome at 6 months CA as determined by DASII<br>Pre-specified secondary outcome(s): maternal adverse events following MgSO4 infusion (to maternal discharge), neurodevelopmental outcome as determined by DASII (6 months), neonatal mortality (after birth)	NA	NA

Study ID	Population characteristics	MgSO4 intervention/exposure	Non MgSO4 comparator	Outcome(s) specified/reported in relation to MgSO4*	Main results relevant to MgSO4	Main conclusion relevant to MgSO4
Gupta 2021 [13]	Inclusion: pregnant women with single or multiple pregnancy < 37 weeks (28-36 weeks 6 days) GA, in labour if the birth was planned or expected within 24 hours<br>Exclusion: women in the second stage of labour or when delivery is imminent within 2 hours; women who received MgSO4 therapy in pregnancy for other reasons; contraindication to MgSO4; women who met at least 1 of the following criteria: hypotension, cardiac rhythm abnormalities, electrolyte abnormalities, ingestion of calcium channel blockers, indomethacin or digitalis in last 24 hours, myasthenia gravis, or indication for emergency caesarean section; women with growth restriction, haemolysis, elevated liver function test results, low platelet syndrome, and retro placental hematoma; severe fetal malformation or chromosomal abnormalities; intrauterine fetal demise<br>N = 100 women	4 g IV bolus LD over 20-30 min followed by 1 g/hour IV MD (discontinued if not delivered by 12 hours) (N = 50 women)<br><br>*IV assumed*	No MgSO4 (N = 50 women)	Reported outcome(s): maternal side effects (flushing, nausea, sweating, hypotension, tachycardia, postpartum haemorrhage, serious side effects), birthweight, Apgar score at 1 and 5 min, neonatal outcomes (neonatal seizures, NICU admission, respiratory distress, mechanical ventilation, NEC, intensive resuscitation, intrauterine growth restriction, neonatal mortality), IVH, PVL, paediatric follow-up 'neurodevelopmental process'	"Neonatal outcomes among women administered MgSO4 and women not administered MgSO4 include the following: Neonatal seizures (2 vs 4%), respiratory distress (46 vs 60%), mechanical ventilation (48 vs 62%), and neonatal enterocolitis (6 vs 0.5%). The difference between Apgar scores of the two groups is statistically insignificant. Resuscitation was needed, 4 versus 6%, in group 2. There were 1 mortality in group 1 and 2 in group 2. In group 1, 44% of neonates needed neonatal intensive care unit (NICU) admission, whereas in group 2, 62.5% of neonates needed NICU admission with a p value of 0.03095, which is found to be not significant between the two groups. In neonates of group 1, the cases administered MgSO4 did not show the sign of intraventricular hemorrhage or	"MgSO4 is the drug currently administered to mothers at the risk of preterm labor for fetal neuroprotection. Further multicenter studies with larger sample sizes exploring immediate adverse outcomes in magnesium-exposed neonates correlated with their serum magnesium concentrations are needed. Further prospective studies must be performed to determine the optimal dose of maternal magnesium for different subgroups of mothers to provide fetal neuroprotection with minimal neonatal adverse outcomes. In the meantime, there is a need for guidelines on the use of MgSO4."

Study ID	Population characteristics	MgSO4 intervention/exposure	Non MgSO4 comparator	Outcome(s) specified/reported in relation to MgSO4*	Main results relevant to MgSO4	Main conclusion relevant to MgSO4
					periventricular leukomalacia, while on the contrary, in neonates of group 2, 4% had ultrasonography suggestive of intraventricular hemorrhage and 6% had periventricular leukomalacia with a p value of 0.14, which is statistically insignificant. Maternal side effects, such as flushing (66 vs 6%), nausea (16 vs 2%), sweating (28 vs 4%), hypotension (2%), tachycardia (4% in group 1), and postpartum hemorrhage, were seen in 4% of women administered MgSO4 and 2% of cases in group 2. No serious side effects were attributed to MgSO4, and the commonest side effect was flushing”	
Gupta 2023 [14]	Inclusion: pregnant women with preterm labour pains at 28-36 weeks GA, with no associated complications<br>Exclusion: second stage of labour, already received MgSO4 during current pregnancy, allergy to drug, renal failure,	4 g IV LD over 20 min followed by 1 g/hour IV for 24 hours or up to delivery (N = 50 women)	No MgSO4 (N = 50 women)	Pre-specified outcome(s): assessment of neonatal wellbeing (Apgar score at 1, 5 and 10 min; NICU admission); all neonates underwent CUS (IVH if any graded and noted); neonatal assessment: Moro’s reflex (at	“APGAR score was better in women who received MgSO4, but the difference was found to be statistically significant only for 5 minutes. Although IVH and periventricular	“Despite these limitations, the findings of present study showed a protective impact of magnesium sulphate against respiratory depression as depicted by lower incidence of

Study ID	Population characteristics	MgSO4 intervention/exposure	Non MgSO4 comparator	Outcome(s) specified/reported in relation to MgSO4*	Main results relevant to MgSO4	Main conclusion relevant to MgSO4
	hypocalaemia, any contraindication to drug (RR < 16/min, absent patellar reflex, urine output < 100 mL during previous 4 hours, neuromuscular disorder)<br>N = 100 women			birth and 6 months), rooting and sucking reflex (at birth) and neck holding reflex (at 4 months); followed up for next 6 months "for outcome"<br>Other reported outcome(s): PVL; neonatal outcome (alive, early neonatal death, late neonatal death, loss to follow up), diagnosis at NICU admission (respiratory distress, perinatal asphyxia, neonatal septicaemia, meconium aspiration syndrome)	leukomalacia rate was higher in group I as compared to that in group II. Although proportion of alive neonates was higher in group I as compared to that in group II yet there was no significant difference between two groups (p=0.961). Incidence of respiratory distress, followed by perinatal asphyxia were most common in both the groups but was not found to be significant statistically (p>0.05)"	Apgar <7 at 5 minutes in exposed group as compared to unexposed group. Moreover, CUS outcome and other neuromotor outcomes were slightly better in magnesium sulphate exposed group as compared to unexposed group, thus showing a possible neuroprotective effect. The study also showed a better survival rate in magnesium sulphate exposed group as compared to unexposed group."
Iqbal 2023 [15]	Inclusion: pregnant women between 28-36 weeks GA, whether carrying a single or multiple pregnancy, in labour, if delivery was planned or expected within 24 hours<br>Exclusion: women in the second stage of labour or whose birth was expected to occur in next 2 hours, pregnant women who got MgSO4 for other reasons, where MgSO4 was contraindicated (RR < 100 mL over preceding 4 hours), women with retroplacental haematoma, low	4 g IV bolus LD over 20-30 min, then 1 g/hour IV MD (N = 199 women)<br><br>*IV assumed*	No MgSO4 (N = 320 women)	Reported outcome(s): neonatal outcomes: mechanical ventilation, respiratory distress, NEC, intensive resuscitation, neonatal seizures, NICU admission, mortality, IVH, PVL; maternal side effects: flushing, sweating, nausea, hypotension, tachycardia	**"**Neonatal outcomes of groups include the following: Mechanical ventilation in group A was 45(22.6%) & 85(26.5%) in group B, Respiratory distress 83(41.7%) vs 191(59.7%), neonatal enterocolitis 11(5.5%) vs 9(2.8%), intensive resuscitation 6(3%) vs 12(3.7%), neonatal seizures 7(3.5%) vs 11(3.4%), NICU	"The development of cerebral palsy in preterm infants may be prevented by administering MgSO4 with a wide margin of safety. Even though MgSO4 has only moderate benefits, it is a secure, affordable drug, so the risk-benefit ratio favors using it."

Study ID	Population characteristics	MgSO4 intervention/exposure	Non MgSO4 comparator	Outcome(s) specified/reported in relation to MgSO4*	Main results relevant to MgSO4	Main conclusion relevant to MgSO4
	platelet syndrome, increase liver function test results, haemolysis, and growth restriction, serious genetic anomalies or fetal malformations, intrauterine fetal death<br>N = 519 women				admission required in group A was 85(42.7%) vs 183(57.1%) in group B, mortality in both groups was 2(1%) vs 4(1.2%).”	
Jamileh 2018 [16]	Inclusion: pregnant women eligible to receive MgSO4 for any indication including pre-eclampsia prevention or neuro-protection, and pregnancy termination < 12 hours, up to 32 weeks GA (confirmed by last menstrual period and first trimester ultrasonography)<br>Exclusion: multiple pregnancy, congenital fetal abnormality, myasthenia gravis, chronic renal failure, < 12 hours before MgSO4 administration, pregnancy termination due to any cause<br>N = 70 women	6 g IV LD over 30 min and 2 g/hour IV for MD 12 hours (N = 70 women)<br><br>*MgSO4 for any indication including fetal neuroprotection*	Nil	Pre-specified outcome(s): parameters from Doppler ultrasonography: “Before and after administration of Magnesium sulfate: Doppler evaluation of uterine (UA), umbilical (UMA) and middle cerebral artery (MCA) performed for pulsatility index (PI), resistance index (RI) and systole/diastole ratio (S/D)”<br>Other reported outcome(s): mother and fetal heart rate; maternal blood pressure	“Umbilical artery pulsatility index (UPI) was significantly decreased after MgSO4 injection (P= 0.001), umbilical resistance index in umbilical artery (URI) and S/D also were significantly decreased (P<0.0001). In fetal MCA Doppler, middle cerebral artery pulsatility index (MPI) (P<0.0001) and middle cerebral artery resistance index (MRI) (P<0.0001) were increased significantly after MgSO4 injection and peak systolic velocity (PSV) had not changed significantly. In uterine artery Doppler, uterine artery pulsatility index (UTPI) and uterine artery resistance index (UTRI) were significantly decreased after drug injection (P<0.0001 in both).”	“Intravenous administration of magnesium sulfate in pregnant women up to 32 gestational weeks resulted in a decrease in PI and RI in umbilical artery and uterine artery, and increased in MCA Doppler indices.”

Study ID	Population characteristics	MgSO4 intervention/exposure	Non MgSO4 comparator	Outcome(s) specified/reported in relation to MgSO4*	Main results relevant to MgSO4	Main conclusion relevant to MgSO4
Jayashree 2021 [17]	Inclusion: all pregnant women of 28-34 weeks GA in labour or anticipated to deliver in next 24 hours for fetal or maternal indications<br>Exclusion: pregnant women with renal disease and liver disease, myasthenia gravis, mitochondrial myopathy, diabetes mellitus, patient refusal<br>N = 50 women	4 g IV LD over 20 min followed by (a) 1 g/hour MD IV for at least 24 hours or until delivery; after 24 hours, unless delivery anticipated the infusion stopped OR (b) 2 g/hour IV MD for at least 12 hours or until delivery; after 12 hours, unless delivery anticipated infusion stopped (N = 50 women)<br><br>*Uncertainty regarding indication – fetal neuroprotection versus tocolysis (mentions tocolysis, but also includes indicated births)*	Nil (all patients received MgSO4)	Pre-specified outcomes: "perinatal outcome for neuro protection, perinatal death and seizure were observed"<br>Other reported outcome(s): Thompson score (normal, mild neuronal abnormality, moderate neuronal abnormality); RDS; MgSO4 infusion delivery interval (<= 12, ≥ 13 hours); Apgar score at 1 and 5 min; perinatal outcome (mortality, normal)	"Among all the preterm in our study, 90% delivered between 30 and 34 weeks. Thompson score revealed that 60 % of the new-born had normal neuronal development. Considering infusion (MgSO4) –delivery interval, 80% of delivery occurring after 13 hours was predominant. The APGAR score at 1 minute was 5.78±1.64 and at 5 minutes 7.44±1.49 was observed. About 92% of the neonates were normal."	"Antenatal magnesium sulphate given to women in established preterm labour conferred significant neuroprotective advantage to the neonate and it is a safe drug to use in antenatal women."
Jin 2022 [18]	Inclusion: VPI born at 24+0 to 31+6 weeks GA and admitted to participating Chinese Neonatal Network NICUs within 24 hours after birth from January 2019 to December 2019<br>Exclusion: infants with major congenital anomalies according to the Chinese Neonatal Network data definition manual, infants with missing information on the 4 practices, infants with missing outcome data	"MgSO4, defined as administration of magnesium sulfate before delivery for any reason" (N = 3094 infants)<br><br>*Any indication, including fetal neuroprotection*	No MgSO4 (N = 2941 infants)	Pre-specified primary outcome(s): early death (within 7 days of age before discharge from the NICU) or severe brain injury (IVH grade III or IV according to Papile's classification)	"Among the 4 evidence-based practices, the incidence of exposure to … MgSO4 was 51.3% (*n* = 3,094)."<br>"The lowest incidence of early death or severe brain injury occurred with exposure to ACS + MgSO4 (aOR 0.38 [95% CI: 0.18, 0.81]) and inborn + ACS + NT (aOR	"More comprehensive use of evidence-based practices was associated with improved survival without severe brain injury among very preterm infants born at <32 weeks gestational age."

Study ID	Population characteristics	MgSO4 intervention/exposure	Non MgSO4 comparator	Outcome(s) specified/reported in relation to MgSO4*	Main results relevant to MgSO4	Main conclusion relevant to MgSO4
	N = 6035 infants				0.39 [95% CI: 0.23, 0.65]).” “Exposure to ACS only was associated with significant lower incidence of death and/or severe brain injury than none (aOR, 0.71; 95% CI: 0.57–0.88), but not MgSO4 only (aOR, 0.97; 95% CI: 0.81–1.17), NT only (aOR, 0.91; 95% CI: 0.76–1.08), or inborn only (aOR, 0.91; 95% CI: 0.72–1.15). The association between number of practices and incidence of early death and/or severe brain injury is as follows: none = 23% (31/138), any 1 = 14% (84/592), any 2 = 12% (185/1,538), any 3 = 9% (202/2,285), and all 4 = 9% (140/1,482).”	
Kasapoğlu 2020 [19]	Inclusion: preterm infants (< 37 weeks GA), born to mothers who were administered antenatal MgSO4 for at least 8 hours before delivery for various indications (neuroprotection, tocolysis or prophylaxis for eclampsia) (exposed group); born to mothers who did not receive MgSO4 (control group)	4.5 g IV LD followed by 2 g/hour MD for at least 8 hours (N = 92 newborns) *MgSO4 for various indications including fetal neuroprotection*	No MgSo4 (N = 147 newborns)	Pre-specified primary outcome(s): failure rate on ABR hearing screening in newborns Other pre-specified outcome(s): neonatal parameters evaluated within first 28 days such as microcephaly, and incidence of postnatal morbidities: requirement for mechanical	“Our results showed that the failure rate in the ABR screening test was lower in the group of newborns exposed to antenatal MgSO4 than in the group of neonates who were not exposed to antenatal MgSO4 (p=0.034). Nevertheless, after adjustment for	“Our results do not suggest a clear and definite benefit from antenatal MgSO4 infusion in respect of hearing impairment in premature newborns. For the usage of MgSO4 as a neuroprotective medication against

Study ID	Population characteristics	MgSO4 intervention/exposure	Non MgSO4 comparator	Outcome(s) specified/reported in relation to MgSO4*	Main results relevant to MgSO4	Main conclusion relevant to MgSO4
	Exclusion: newborns who had a history of intrauterine infections, lethal congenital abnormalities, craniofacial anomalies, and who died after birth<br>N = 239 newborns			ventilation (intubation), neonatal sepsis, exposure to ototoxic agents, meningitis, IVH, ROP, RDS, phototherapy requirement, BPD; rate of pathological EEG, visual evoked potential and early Denver Developmental Screening test<br>Other reported outcomes: being treated in NICU, small for gestational age	covariates, including betamethasone and gestational age at delivery (day), there was no statistically significant association between AAM and the failure rate in ABR screening (p=0.07)."	hearing impairment in premature newborns, further large scale and carefully designed studies are warranted to reach a definite conclusion."
Kumar 2023 [20]	Inclusion: women with established preterm labour (regular uterine contraction with cervical dilatation of ≥ 4 cm) at < 32 weeks GA and women requiring iatrogenic preterm labour/caesarean at < 32 weeks GA<br>Exclusion: myasthenia gravis, acute renal failure, false preterm labour, congenital malformation, congenital infection, > 32 weeks GA and drug delivery interval of less than 4 hours<br>N = 211 women	4 g IV over 20 min (N = 116 women)	No MgSO4 (N = 95 women)	Pre-specified/reported outcome(s) (from flowchart): fetal outcome (Apgar at 5 min (reports < 7), mechanical ventilation/tracheal intubation (also reports on resuscitation – bag and mask only, chest compression, hypotension, early onset sepsis, surfactant), seizures, NEC (reports stage > 2), IVH (reports grade 1-2 and 3-4)or PVL), neonatal imaging (CUS), maternal outcome (hypotension, absent reflexes, blurred vision, flushing, nausea, vomiting, sweating, postpartum haemorrhage), lab reports (cultures/CRP), follow-up (neonatal death, cerebral palsy, milestones and neurodevelopmental	"Fewer neonates in the MgSO4 group required intubation at birth (32% vs. 52%) or chest compression (4% vs. 6%); however, the difference was not statistically significantly (p = 0.175 and p = 0.329). Neonatal brain ultrasound done in first month showed a significant reduction intra-ventricular haemorrhage of severe grade 3–4 IVH in the MgSO4 group (p = 0.016). MgSO4 administration was associated with a decrease in neonatal mortality before discharge (p = 0.039). Follow-up at 3 years	"The benefits from single smaller dose magnesium sulphate 4 gram prove its potential to be used for foetal neuroprotection in any healthcare setting without any maternal concerns. While the lower dose did not prove any significant difference in mild brain lesion, however there was significant difference in severe brain lesion, cerebral palsy, delayed milestones, and mental development index. Large-scale studies are still required to compare smaller doses of magnesium sulphate

Study ID	Population characteristics	MgSO4 intervention/exposure	Non MgSO4 comparator	Outcome(s) specified/reported in relation to MgSO4*	Main results relevant to MgSO4	Main conclusion relevant to MgSO4
				performance at 3 years) (reports on delayed milestones, visual impairment, mental development index < 85 on Bayley Scales of Infant Development, cerebral palsy)	showed a significant reduction in delayed milestones, visual impairment, Bayley score < 85 (p = 0.015). MgSO4 treatment antenatally was associated with lower risk of Cerebral Palsy (2.6% vs. 23.2%, p < 0.001)."	with current standard one in order to ascertain the effective dose of drug required for neuroprotection."
Mamatha 2023 [21]	Inclusion: pregnancy period of < 34 weeks GA due to maternal and fetal indications; preterm labour and threatened preterm labour; PPROM<br>Exclusion: full established preterm labour (active stage), stage 3 and 4 fetal growth restriction, severe oligohydramnios, chorioamnionitis, fetal distress, abruptio placenta/bleeding placenta praevia, eclampsia/DIC: severe pre-eclampsia with imminent symptoms<br>N = 52 women	4 g IV LD over 20 min followed by 10 g in 500 mL at 20 mL/hour for 24 hours (0.4 g/hour) IV MD<br>Total dose 14 g<br>(N = 52 women)	Nil	Pre-specified outcome(s): monitoring (pulse, BP, RR < SpO2, knee jerk, urine output, FHR monitoring); adverse effects (itching, flushing, giddiness, disorientation, chills, fever, hypotension, difficulty in breathing); details regarding delivery (gestational age at birth, mode of birth, Apgar score, birthweight, condition of the baby, SNCU admission, discharge, follow up of baby – in 3rd and 6th postnatal months for developmental milestones [3 months: developmental milestones normal, delayed development, atypical febrile convulsions, death, hearing defect; 6 months: severely delayed development, death]; neurosonogram (only for few cases at birth)	"96.2% had no reactions, minor reactions noted were tachycardia, blurring of vision, absent knee jerk only among 2 persons. Among 23 babies had normal birth weight,27 were born with low birth weight and 5 babies were born with very low birth weight. In our study 3 perinatal deaths occurred. 2 cases developed delay in development. 1 at 3 months and another 1 case had severe gross motor dysfunction at 6 months postnatal follow up."	"In present study, MgSO4 introduction in the protocol of management of preterm gestation for admitted antenatal cases for prevention of neurological abnormalities is very much effective and it is feasible in a tertiary obstetric healthcare. Administration of MgSO4 in preterm management protocol is safety and cost effective with less side effects to the mother and neuroprotective effects to the newborn."

Study ID	Population characteristics	MgSO4 intervention/exposure	Non MgSO4 comparator	Outcome(s) specified/reported in relation to MgSO4*	Main results relevant to MgSO4	Main conclusion relevant to MgSO4
Manoj 2017 [22]	Inclusion: preterm babies born to eligible pregnant women with singleton, twin, or triplet fetuses, between 24 and 34 weeks GA, if birth was expected or planned within 24 hours<br>Exclusion: fetus with severe malformations or chromosomal abnormalities, maternal hypotension, cardiac rhythm or electrolyte abnormalities, renal or hepatic insufficiency, maternal contraindications to MgSO4, unwillingness of the obstetrician to intervene for fetal benefit, and receipt of MgSO4 within the previous 12 hours<br>N = 126 women, 134 neonates; 16 were excluded, leaving 118 neonates	4 g IV bolus LD followed by 1 g/hour for 24 hours or until birth, whichever comes first (N = 58 neonates)	Matching placebo (normal saline) (N = 60 neonates)	Pre-specified primary outcome(s): the composite incidence of death (neonatal prior to discharge) and IVH (CUS in surviving preterm infants)<br>Pre-specified secondary outcome(s): resuscitation at birth, RDS, surfactant administration, type and duration of ventilator support (CPAP, invasive ventilation), hypoxic ischemic encephalopathy, apnoea of prematurity, sepsis, anaemia requiring transfusion, NEC (stage 2 or more), PDA<br>Other reported outcome(s): maternal adverse events, periventricular echodensity, 5 min Apgar < 6	"There were 6 deaths in cases and 11 deaths in controls (relative risk [RR]: 0.54; 95% confidence interval [CI]: 0.2173-1.369; p=0.18). A statistically significant reduction in the number of IVH was observed in cases (n=1) compared to controls (n=9) (RR: 0.11; 95% CI: 0.0145-0.897; p=0.016) with a number needed to treat of 7."	"We conclude that antenatal MgSO4 resulted in a significant reduction in the risk of IVH and may be considered for the primary prevention of IVH in preterm infants of gestational age 34 weeks and below. The above treatment is cost-effective, without neonatal side effects and clinically significant for a prophylactic intervention."
Marnal 2023 [23]	Inclusion criteria: all patients admitted with preterm labour with < 34 weeks GA<br>Exclusion criteria: intrauterine deaths<br>N = 37 women first period<br>N = 47 women second period	According to FOGSI guidelines i.e. 4 g IV bolus LD over 20 min followed by 1 g/hour MD for 24 hours or until delivery, whichever is earlier (N = 37 women and 47 women)	No MgSO4	Pre-specified outcome(s): number of patients who received MgSO4, reasons for not being able to follow the recommendations, measures taken to correct it, changes in clinical practice	First period: "only 6 patients (16%) received the drug. 31 patients (84%) had not received the drug due to various reasons… to which they were delivered immediately and did not receive MgSO4. Lack of awareness about the need of MgSO4 as neuro prophylaxis among labor room staff was also an important factor"	"Formulation of protocols, formation of a dedicated team, and constant auditing to ensure adherence to protocol benefits the patient outcome."

Study ID	Population characteristics	MgSO4 intervention/exposure	Non MgSO4 comparator	Outcome(s) specified/reported in relation to MgSO4*	Main results relevant to MgSO4	Main conclusion relevant to MgSO4
					Second period: "43 patients (93%) were given neuro prophylaxis. 02 patients presented in 2nd stage of labor and were delivered within 30 min of admission, and 02 patients had APH due to abruption and needed emergency LSCS. These 04 patients (07%) did not receive MgSO4. Even though 100% coverage could not be achieved significant change in coverage was noted."	
Mazhar 2023 [24]	Inclusion: women admitted with active imminent preterm labour with/without PPROM (cervical dilatation > 4 cm with/without PROM) or those with planned preterm birth for fetal or maternal indication at 28-34 weeks GA; included women with severe pre-eclampsia/eclampsia with preterm labour<br>Exclusion: major fetal abnormalities or intrauterine fetal death; women with relative contraindications to MgSO4 such as electrolyte disorders maternal cardiac or renal disease<br>N = "The sample size was 88" (assume this is women); 114 neonates	4 g IV LD over 30 min; if birth delayed > 12 hours a repeat bolus recommended [but no case of prolonged labor, so a second bolus was not given within study (88 women)	Nil (all patients received MgSO4)	Pre-specified other outcome(s): maternal monitoring (pulse rate, BP, RR, tendon reflexes), fetal heart rate through labour; "dependent variables were": Apgar score, nursery and NICU admissions, monitoring for HIE, frequency of neonatal seizures; NICU admissions, neonatal cranial ultrasound finding of IVH; PVL<br>Other reported outcome(s): neonatal resuscitation, neonatal mortality; maternal size effects: major side effects, hypotension, flushing and sweating, palpitation,	"and the mean Apgar score at 5 minutes was recorded as 7.11 (±1.208)… Neonatal seizures were observed in 3 (2.6%), intraventricular hemorrhage in 2 (1.754%), Periventricular leukomalacia (PVL) 1(0.877%), and neonatal mortality in 5 (4.38%).	"Magnesium Sulphate is a safe drug that plays an important role in protecting immature brains. Four-gram bolus is a sufficient dose as compared with infusion, which requires additional human resources and risks attached to prolonged infusions."<br>"Magnesium Sulphate is a safe and effective molecule that plays a key role in protecting the immature brain. Further large-scale multicenter trials are needed to signify the

Study ID	Population characteristics	MgSO4 intervention/exposure	Non MgSO4 comparator	Outcome(s) specified/reported in relation to MgSO4*	Main results relevant to MgSO4	Main conclusion relevant to MgSO4
				headache, nausea and vomiting, hyporeflexia		dose, duration, and effectiveness of this regimen.”
Medhi 2023 [25]	Inclusion: pregnant women with preterm labour, planned preterm birth due to maternal or fetal indication between 28 to 32 weeks GA<br>Exclusion: NR; however, women who could not be followed up were not included<br>N = 72 women	4 g IV LD over 15 min, followed by 1 g/hour MD infusion until birth or up to 24 hours, whichever came first (as per FOGSI 2017) (N = 37 women)	No MgSO4 (N = 35 women)	Pre-specified outcome(s): maternal side effects: hot flushes, nausea, vomiting, muscle weakness, discomfort at injection site, respiratory depression; neonatal: birth weight, Apgar score at 1 and 5 mins, NICU admission, requirement for respiratory support (reported on: CPAP, intubated, oxygen by hood), presence of IVH (CUS within 14 days of birth), morbidities (RDS, NEC, septicaemia), signs and symptoms (weak cry, tachypnoea, chest retraction, lathery, seizure, hypotension, apnoea, sepsis), condition at discharge; longer-term outcomes: 2 months CA: social smile; 4 months CA: neck holding; 6 months CA: sitting with support	"The number of babies who developed IVH in the non-MgSO4 group was significantly greater (5/35, 14.3 %) than in the MgSO4 group (3/35, 8.6%) (*p* value = 0.452). In this study 3 (8.6%) ELBW babies in nonMgSO4 group had IVH compared to 2 (5.7%) in MgSO4 group. 2 (5.7%) VLBW babies in nonMgSO4 group had IVH while 1 (2.9%) VLBW babies in MgSO4 group had IVH. According to the gestational age in nonMgSO4 group 20% (7/35) of the babies between 28 to 30 weeks required intubation compared to 11.4% (4/35) babies in MgSO4 group. 14.3% (5/35) of the babies between 30 to 32 weeks in nonMgSO4 group required intubation where only 5.7% (2/35) babies in MgSO4 group required intubation. (*p* value = 0.017). 5.7%	“Antenatal MgSO4 appears to minimize the likelihood of invasive mechanical ventilation, the need for continuing respiratory support, intraventricular hemorrhage. Antenatal MgSO4 has similar effects across a range of preterm gestational ages.”<br>“Magnesium sulphate has great potential for use in resource restricted settings due to its ease of storage, widespread availability and low cost. We believe that the introduction of such an inexpensive, easy-to-administer treatment with no significant maternal side effects, in the national guidelines, may lead to an important improvement of the preterm infant prognosis and it should be considered a component part of the prenatal prophylactic

Study ID	Population characteristics	MgSO4 intervention/exposure	Non MgSO4 comparator	Outcome(s) specified/reported in relation to MgSO4*	Main results relevant to MgSO4	Main conclusion relevant to MgSO4
					(2/35) babies between 30 to 32 weeks of gestation in nonMgSO4 group had delayed milestones, while no baby showed delayed milestones in MgSO4 group.”	treatment of preterm infants.”
Millogo-Traoré 2022 [26]	Inclusion (both groups): presented with imminent risk of preterm delivery between 28 and 32+6 weeks GA, single or multiple pregnancy, informed consent<br>Inclusion (exposed group): patients at Yalgado Ouédraogo University Hospital (where the brain maturation protocol is applied), received MgSO4 for fetal neuroprotection, newborns delivered at UHC Yalgado Ouédraogo<br>Inclusion (unexposed group, matched to exposed group): UHC-Bogodogo patients (where the brain maturation protocol is not yet applied); did not receive MgSO4 for fetal neuroprotection, newborns delivered at the Bogodogo University Hospital<br>Exclusion: known chromosomal or genetic abnormalities or malformations, fetal death in utero, contraindication to the use of MgSO4<br>N = 210 women	4 g IV LD over 30 min followed by 4 g IM every 4 hours for 12 hours (N = 70 women)<br><br>*“During our study, 85.7% received a course of magnesium sulphate in the context of brain maturation.”*	No MgSO4 (N = 140 women)	Pre-specified outcome(s): neonatal mortality (first 28 days) and neonatal morbidity; neurological abnormalities (tonus anomaly, archaic reflex anomaly, consciousness anomaly) at day 7 and 28<br>Other reported outcome(s): aspects of MgSO4 treatment (place of administration, cures for cerebral maturation, monitoring, side effects, management of side effects and signs of overdose); Apgar score < 7 at 10 min, resuscitation at birth, hospitalisation after transfer to paediatrics, neurological disorders at day 7 and 21	“The mortality rate, as well as neonatal neurologic complications, was higher with unexposed than with exposed. Although antenatal exposure to magnesium sulfate was not statistically associated with mortality and morbidity in newborns at a threshold of 0.05%, it has shown an overall good neurological prognosis in newborns exposed.”	“Our study shows that although prematurity is a real problem in newborns because of its high morbidity and mortality, the administration of magnesium sulphate to pregnant women between 28 and 32 weeks’ gestation is a real asset in reducing these neonatal complications. However, a study on a larger population is necessary to better appreciate the determinants of this influence. A generalisation of the use of magnesium sulphate for neuroprotective purposes could even be envisaged in our countries.”

Study ID	Population characteristics	MgSO4 intervention/exposure	Non MgSO4 comparator	Outcome(s) specified/reported in relation to MgSO4*	Main results relevant to MgSO4	Main conclusion relevant to MgSO4
Mohan 2023 [27]	Inclusion: women at risk of preterm birth (spontaneous/induced) between 28-32 weeks GA where birth is planned or definitely expected within 24 hours, singleton or twin pregnancy, including women with severe pre-eclampsia, eclampsia, induced preterm for maternal or fetal indications<br>Exclusion: multi-fetal gestation > 2 fetuses, women with preterm labour induced from outside hospitals, preterm delivery from outside hospitals<br>N = 150 women	4 g IV LD over 20 min followed by 1 g/hour MD IV for 24 hours (N = 75 women)	No MgSO4 (N = 75 women)	Pre-specified outcome(s): maternal obstetric outcome, neonatal outcomes including IVH (all grades), PVL, neonatal seizures, RDS, need for supplemental oxygen at 36 weeks, BPD, need for mechanical ventilation, NEC, neonatal death, infant neurodevelopmental outcomes up to 1 year (6 weeks, 6 months, 1 year) (Trivandrum Developmental Screening Chart - substantial gross motor dysfunction, major neurological disability, blindness, deafness, infant death).<br>Other reported outcome(s): mode of birth, gestational age at birth, birthweight (and small/large for gestational age), Apgar < 7 at 1 and 5 min, duration of NICU stay, respiratory distress needed CPAP, neonatal sepsis, moderate HIE, severe HIE, ASD, PDA, DIC, neonatal shock, neonatal depression, intrinsic AKI, prerenal AKI, neonatal hyperbilrubinaemia, brain abscess communicating hydrocephalus, transient tachypnea of newborn, pneumothroax, anemia of prematurity, cause of death,	"The neonatal mortality (6% versus 12.5%; relative risk [RR],0.48; 95% confidence interval [CI], 0.17-1.3), residual neonatal morbidity(3.8% v/s 11.6%; RR, 0.32;95% CI, 0.09-1.16), the neurological disabilities like cerebral palsy(1.3% v/s 2.18%; RR,0.47;95% CI, 0.04-5.17) and the combined death or cerebral palsy (8.1% v/s 18.3%; RR,0.44;95% CI, 0.17-1.10) were less frequent in the Group A who received magnesium for neuroprotection, but the differences are not significant statistically. Respiratory distress (78.4% v/s 55.4%~; RR,0.72; 95% CI, 0.5-0.9) and necrotizing enterocolitis (3.6% v/s 14.7%; RR,0.24; 95% CI, 0.07-0.82) were significantly reduced in the Group A."<br><br>~Likely to be an error in reporting of group order	"Magnesium sulphate for fetal neuroprotection is found to reduce the incidence of neonatal mortality, total neonatal morbidity and neurological disabilities but there is no statistical significance. However, significant reduction is seen in the respiratory distress and necrotizing enterocolitis in the neuroprotection group. Thus concluding there is definitely a role of magnesium sulphate clinically, in having a favourable neonatal outcome, although statistically insignificant."

Study ID	Population characteristics	MgSO4 intervention/exposure	Non MgSO4 comparator	Outcome(s) specified/reported in relation to MgSO4*	Main results relevant to MgSO4	Main conclusion relevant to MgSO4
				combined death or cerebral palsy		
Muhammed 2019 [28]	Inclusion: pregnant women 28 to 34 weeks GA, Sulaimani Maternity Teaching Hospital, in preterm labour<br>Exclusion: NR<br>N = 60 women	4 g IV bolus LD over 20 min followed by 1 g/hour MD (max dose 12 g) (N = 28 women)	Placebo (normal saline) (N = 32 women)	Pre-specified outcome(s): Apgar score 1 min and 5 min, NICU admission (time/admission period), brain ultrasound at 1-week (intracranial haemorrhage), neurological assessment at 28 days (abnormal signs like hypotony, abnormal tone, abnormal reflexes and state of consciousness)<br>Other reported outcome(s): death/mortality during follow up, cost of hospital stay (difference)	"The Apgar score for the group who received the drug was more both at one and five minutes. Mortality rate in this group was (3.5%) at the time the group with placebo had mortality rate of (22%) (Pvalue was 0.02), duration of the intensive care unit stay was less in the first group. Intraventricular hemorrhage was present in 1 case of the second Group."	"Intravenous magnesium administration is cost effective as saved 40000 $ during the period of follow up. Magnesium sulphate has neuroprotective effect on neonate. There was decrease in mortality in the group who received it. Duration of the intensive care unit stay was less, too. Larger sample size may be necessary to have a better estimate of outcome."
Musiime 2021 [29]	Inclusion: all live-born neonates with birthweight < 1000 g, admitted to Tygerberg Hospital between 1 January 2016 and 31 December 2016<br>Exclusion: Outborn neonates admitted to Tygerberg Hospital after day 28 of life<br>N = 256 infants	MgSO4 (delivery and perinatal intervention) (N = 122 infants)	No MgSO4 (N = 134 infants)	Pre-specified outcome(s)*: received antenatal MgSO4	Summary of Table 1: antenatal MgSO4 was administered to 47.7% of infants 23-34 weeks (44.0% (23-26 weeks), 50.6% (27-30 weeks) and 38.9% (31-34 weeks)).	"Morbidity and mortality rates remain high among ELBW neonates… administration of antenatal magnesium sulphate and antenatal steroids should be optimised to minimise morbidity."
Nunes 2018 [30]	Inclusion: patients with premature newborn deliveries between 24- and 32-weeks GA<br>Exclusion: NR	4 g IV LD over 30 min (N = 19 women)	No MgSO4 (N = 56 women)	Pre-specified outcome(s): "Hemodynamic effects were studied as outcomes": HR (normal, abnormal), RR	"There was no significance relation detected between the use of MgSO4 and the	"Conclusions: MgSO4 does not appear to influence hemodynamic factors as a cause of the

Study ID	Population characteristics	MgSO4 intervention/exposure	Non MgSO4 comparator	Outcome(s) specified/reported in relation to MgSO4*	Main results relevant to MgSO4	Main conclusion relevant to MgSO4
	N = 75 women, 99 newborns; 5 newborns excluded, leaving 95 newborns			(normal, abnormal), temperature (normal, abnormal), oxygen saturation (≥ 95%, < 95%), haemoglucotest (normal, abnormal), haemoglobin (≥ 16.4 g/dL, < 16.4 g/dL), type of mechanical ventilation (non-invasive, endotracheal)	hemodynamic characteristics."	neuroprotection in premature NB."<br>"Despite the lack of statistical significance in the relations between the studied variables, it is recommended to carry out further researches with continuous hemodynamic monitoring to identify changes that may guide the development of preventive approaches. MgSO4 alone may not be able to block all paths leading to brain palsy, since the injury appears to be related to the interaction of multiple factors rather than a single cause."
Okulu 2024 [31]	Inclusion: infants born between 24+0 and 31+6 weeks GA and admitted to NICU; had at least one echocardiographic assessment<br>Exclusion: infants with major congenital malformations, infants who died within the first 72 hours of life<br>N = 300 infants (after excluding 34 infants)	4 g IV LD over 30 min, followed by 1 g/hour MD until delivery, discontinued if delivery was no longer imminent or maximum 24 hours (N = 98 infants)	No MgSO4 (N = 202 infants)	Pre-specified outcome(s): neonatal serum Mg2+ level at 24 hours of age, neonatal outcomes such as: RDS, surfactant use, IVH, PDA, NEC, ROP, BPD, length of hospital stay, mortality; any PDA, moderate-to-large PDA, medical treatment (hsPDA), PDA ligation, open PDA at discharge	"hsPDA rates were similar in the infants exposed and not exposed to antenatal MgSO4, when adjusted for antenatal steroid administration, gestational age, and birth weight (OR: 1.6, 95% CI: 0.849–3.118, p ¼ 0.146). The rates of PDA ligation and open PDA at discharge were similar	"Although antenatal MgSO4 exposure may increase the incidence of hsPDA, it may not affect the rates of PDA ligation or open PDA at discharge. Further studies are required to better evaluate the dose-dependent outcomes and identify the MgSO4 dose that not only provides

Study ID	Population characteristics	MgSO4 intervention/exposure	Non MgSO4 comparator	Outcome(s) specified/reported in relation to MgSO4*	Main results relevant to MgSO4	Main conclusion relevant to MgSO4
					between the groups. A cumulative MgSO4 dose of >20 g was associated with an increased risk of hsPDA (crude OR: 2.476, 95% CI: 0.893–6.864, p ¼ 0.076; adjusted OR: 3.829, 95% CI: 1.068–13.728, p ¼ 0.039). However, the cumulative dose had no effect on the rates of PDA ligation or open PDA at discharge." "There were no differences in the rates of RDS, surfactant use, NEC, IVH, BPD, andmortality between the groups (p > 0.05). Infants exposed to antenatal MgSO4 required treatment for ROP more frequently than those not exposed to MgSO4 (p = 0.010). The mean length of hospital stay was longer in infants exposed to antenatal MgSO4 than in those not exposed to MgSO4 (p = 0.004…"	neuroprotection but also has the lowest risk of adverse effects."
Ozer Bekmez 2021 [32]	Inclusion: preterm infants born 24 to 32 weeks GA<br>Exclusion: advanced resuscitation in the delivery room, cardiovascular instability requiring treatment with	4 g IV LD over 30 min followed by 1 g/hour MD until delivery (N = 66 infants)	No MgSO4 (N = 64 infants)<br><br>(women who refused MgSO4, had prenatal care in another centre	Pre-specified primary outcome(s): near-infrared spectroscopy: rcSO2 and cFTOE levels during the first 72 hours of life	"There was no statistically significant difference in rcSO2 and cFTOE levels between the groups."	"We could not detect any difference in cerebral oxygenation or oxygen extraction between preterm infants who received fetal

Study ID	Population characteristics	MgSO4 intervention/exposure	Non MgSO4 comparator	Outcome(s) specified/reported in relation to MgSO4*	Main results relevant to MgSO4	Main conclusion relevant to MgSO4
	inotropes, > 12 hours since discontinuation of previous MgSO4 infusion, anaemia, major congenital and/or chromosomal anomalies<br>N = 130 infants	(included MgSO4 for neuroprotection of pre-eclampsia)	and were referred for level III NICU, were admitted to emergency department and gave birth immediately)	Pre-specified secondary outcome(s): severe IVH (grade 3-4) and mortality<br>Other pre-specified outcome(s): neonatal morbidities and mortality: RDS, duration of non-invasive and mechanical ventilation, NEC, BPD, duration of hospitalisation, hsPDA<br>Other reported outcome(s): late onset sepsis, SIP	"We observed that MgSO4 treatment has a significant effect both on severe IVH (adjusted odds ratio: 0.12, 95% CI: 0.02–0.6, p= 0.009) and mortality rates (adjusted odds ratio: 0.12, 95% CI: 0.017–0.99, p= 0.049) but not on cerebral hypoxia (adjusted odds ratio: 0.78, 95% CI: 0.25–2.4, p= 0.68) when corrected for GA and BW in regression analysis."	MgSO4 treatment or not during the first 72 hours of life. Although it is not the primary outcome of our study, fetal MgSO4 therapy was found to be an independent factor that reduces severe IVH rates despite the lower GA and BW in the treatment group."<br>"In early postnatal life, the effect of antenatal MgSO4 treatment on cerebral tissue oxygenation could not be demonstrated with NIRS readings. The reduction in IVH rates with MgSO4 therapy should be supported with adequately powered further clinical studies. Well-designed studies that focus on to clarify the underlying mechanisms of neuroprotection are still required."
Ozer Bekmez 2023 [33]	Inclusion: inborn preterm infants with 24+0 to 31+6 weeks GA were enrolled<br>Exclusion: babies who underwent advanced resuscitation in the delivery room, inotropic	4 g IV LD over 30 min followed by 1 g/hour IV MD for up to 24 hours (N = 310 infants)	No MgSO4 (women who declined to receive MgSO4, received prenatal care in another centre and referred to level III NICU,	Pre-specified primary outcome(s): incidence of gastrointestinal complications, including feeding intolerance, NEC (stage 2 or 3), and SIP	"The MgSO4 group was more likely to have bronchopulmonary dysplasia, prolonged invasive ventilation, necrotizing enterocolitis,	"This study highlighted the effect of MgSO4 treatment and the potential superiority of serum Mg level as a predictor of immediate

Study ID	Population characteristics	MgSO4 intervention/exposure	Non MgSO4 comparator	Outcome(s) specified/reported in relation to MgSO4*	Main results relevant to MgSO4	Main conclusion relevant to MgSO4
	treatment due to haemodynamic instability in the first 7 days of life, > 12 hours since the discontinuation of MgSO4 treatment, severe anaemia, and major congenital/chromosomal anomalies<br>N = 584 infants	Of babies with recorded serum magnesium level at 6th hour (N = 231 infants): MgSO4 concentration ≥ 2.5 mg/dL (N = 85 infants) vs < 2.5 mg/dL (N = 146 infants)	admitted to emergency department for urgent delivery) (N = 274 infants)	Pre-specified secondary outcome(s): "other preterm morbidities"<br>Other reported outcome(s): clinical characteristics based on MgSO4 versus control and high vs. low serum magnesium advanced resuscitation in delivery room, RDS, BPD, hsPDA, full enteral nutrition on day 14, time to full feeds, duration of invasive ventilation, and duration of non-invasive ventilation	delayed enteral nutrition, and feeding intolerance (P < 0.05). MgSO4 treatment was shown as an independent risk factor for feeding intolerance when corrected for confounders (odds ratio 2.13, 95% confidence interval: 1.4–3.1, P = 0.001). Furthermore, serum Mg level significantly correlated with feeding intolerance (r = 0.21, P = 0.002)."	neonatal outcomes, particularly delayed enteral nutrition and feeding intolerance. Further studies are warranted to ascertain the optimal serum Mg concentration of preterm infants in early life to provide maximum benefit with minimal side effects."<br><br>"This study indicates an association between high concentrations of serum magnesium and feeding intolerance, delayed full enteral nutrition, and prolonged mechanical ventilation."
Özlü 2019 [34]	Inclusion: infants ≤ 32 weeks GA, completed antenatal steroid doses, inborn infants hospitalised in our NICU, births 2011-2012 and 2014-2016 (MgSO4 introduced in 2013)<br>Exclusion: multi-fetal pregnancies (except diamniotic twins), infants with congenital abnormalities<br>N = 280 infants	6 g IV LD over 30 min, followed by 2 g/hour MD (discontinued after 24 hours if delivery had not occurred and not determined imminent) (N = 108 infants)	No MgSO4 (historic group) (N = 172 infants)	Pre-specified other outcome(s): neonatal variables: resuscitation at birth, need for mechanical ventilation, duration of mechanical ventilation, infection (early onset sepsis), symptomatic PDA, starting day of enteral feeding, time of full enteral feeding, NEC, ROP, CLD/BPD, duration of hospitalisation, IVH including grade, feeding intolerance,	"Respiratory Distress Syndrome (RDS), intraventricular hemorrhage, severe intraventricular hemorrhage (grade 3 and 4), ROP and BPD were less in Mg (+) group compared to Mg (-) group but it is not significant statistically (p>0,05) except RDS(p<0,05)"	"This retrospective study showed that antenatal exposure to MgSO4 did not have any effect on either morbidities or mortality in premature infants."<br>"In conclusion, although improvements of neonatal outcome obtained with MgSO4 are of potential clinical significance, other managements to

Study ID	Population characteristics	MgSO4 intervention/exposure	Non MgSO4 comparator	Outcome(s) specified/reported in relation to MgSO4*	Main results relevant to MgSO4	Main conclusion relevant to MgSO4
				Other reported outcome(s): death, duration of oxygen therapy, duration of ventilator support		decrease preterm morbidities are needed. More research is needed to assess the protective effect of MgSO4 alone or in combination with other neuroprotective therapy. Local protocols should be developed with respect to its dosing and indications."
Padmapriya 2023 [35] (and Niveditha 2020) [58]	Inclusion: all women with singleton, twin or triplet pregnancies admitted for preterm labour and induced preterm birth (fetal or maternal indications) for delivery between 24- and 33-weeks GA<br>*Note: thesis specifies < 33 weeks*<br>Exclusion: major fetal abnormalities, intrauterine fetal death between 24 and 33 weeks, incomplete medical records; relative contraindications for MgSO4 in protocol: electrolyte disorders, renal failure, defined as rapidly progressive loss of renal function characterized by oliguria (quantified as less than 400 mL/day), maternal cardiac arrhythmia during this pregnancy, myasthenia, ingestion of calcium channel blockers during the	4 g IV LD over 30 min, followed by 1 g/hour MD until birth, for maximum 12 hours (N = 100 women)<br>*Note: thesis reports 24 hours maximum*<br><br>Comparisons made between 2 MgSO4 duration groups: < 6 hours (N = 16 women) versus > 6 hours (N = 84 women)	Nil (all patients received MgSO4)<br>*Note: thesis reports a No MgSO4 group (N = 100 women)*	Publication:<br>Pre-specified outcome(s): cerebral palsy and achievement of milestones (2, 4, 6 months)<br><br>Thesis:<br>Flow diagram: monitor: maternal pulse, BP, RR, tendon reflexes; after delivery assessments of neonate: neonatal death, Apgar score, NEC, IVH, PVL<br>Other reported outcome(s): Apgar at 1 min, 5 min, birthweight, neurological development of babies at end of 2 months (achieved, delayed), 4 months, 6 months (achieved, delayed, cerebral palsy)	"Among 100 patients with preterm labour who was given magnesium sulphate, only 14 % shows features of cerebral palsy and 20 % babies shows neurological abnormalities at 6 months compared to groups who has not given magnesium sulphate where > 30 % of babies at 6 months shows neurological abnormalities. Finally neuroprotection was better in mothers without PPROM, those who got magnesium sulphate infusion > 6 hours and in babies whose weight > 1.5 kg."	"This study demonstrates the feasibility of implementing a protocol to use magnesium sulphate among gravidas at imminent risk of delivery before 33 weeks of gestation to prevent cerebral palsy."<br><br>Thesis: "Widespread adoption worldwide of this relatively inexpensive, easy-to-administer treatment would lead to important global health benefits for infants born preterm."

Study ID	Population characteristics	MgSO4 intervention/exposure	Non MgSO4 comparator	Outcome(s) specified/reported in relation to MgSO4*	Main results relevant to MgSO4	Main conclusion relevant to MgSO4
	previous 2 hours, and "urgent delivery"<br>*Note: thesis specifies < 28 weeks*<br>N = 100 women<br>*Note: thesis reports N = 200 women*				Thesis: "Mgso4 given for > 6 hours shows improved neuroproctection in babies compared to less duration of mgso4 infusion at 2 months… at 4 months… even at 6 months…"	
Pandey 2024 [36]	Inclusion: pregnant women between 28-32 weeks GA, presenting to the emergency department with abdominal pain associated with cervical dilatation (> 4 cm) at risk of imminent preterm birth (delivery definitely planned within 24 hours) during a period of 2 years<br>Exclusion: pregnant women < 28 weeks GA and > 32 weeks GA<br>N = 20 women	4 g IV LD over 20-30 min, followed by 1 g/hour IV MD for 24 hours before delivery or up to delivery (N = 20 women)	Nil (all women received MgSO4 – outcomes reported by GA group)	Pre-specified outcome(s): neonatal seizures, deaths and gross motor dysfunction (followed up for 2 years)	"At our study among the preterm neonates 15 cases (75%) shows better outcome, 2 cases(10%) of neonatal seizures, 2 cases (10%) shows neonatal deaths, and 1 case(5%) of Gross Motor dysfunction noticed."<br>"Antenatal MGSO4 has better role when given at 28-32 weeks of gestation as only 2 neonates (10% of total preterms) born at 32-34 weeks gestation developed neonatal seizures."	"When antenatal MgSO4 administered at an appropriate dose with proper monitoring, there is no evidence of harm to the fetus, neonatal or mother and it helps in neuroprotection of the preterms by preventing neonatal seizures, neonatal deaths, Gross Motor dysfunction in preterms"
Parashi 2017 [37]	Inclusion: pregnant women with PPROM at 34 weeks GA, at Akbarabadi hospital, with informed consent<br>Exclusion: hypertension, pre-eclampsia, trauma, gestational or aggravated diabetes, any type of metabolic disease affecting the pregnancy outcome, long-term	6 g IV LD over 20-30 min, followed by 2 g/hour over 12 hours before labour (N = 60 women)	Control (conventional treatment with normal saline infusion) (N = 60 women)	Pre-specified primary outcome(s): IVH (and grade)<br>Other reported outcome(s): laboratory results: platelet, haemoglobin, leukocyte, sodium, potassium, calcium, blood sugar, ICU stay, stillbirth ("not seen")	Any grade IVH: "the rate of IVH as the main outcome was 5% and 1.6% in the intervention and control groups, respectively (P = 0.981)."<br>Grade III-IV:<br>"No intraventricular brain hemorrhage occurred in	"Magnesium sulfate needs more evaluation in prevention of intraventricular hemorrhage in infants of mothers with premature rupture of membranes at 34 weeks."

Study ID	Population characteristics	MgSO4 intervention/exposure	Non MgSO4 comparator	Outcome(s) specified/reported in relation to MgSO4*	Main results relevant to MgSO4	Main conclusion relevant to MgSO4
	drug use, and gestational histories affecting the pregnancy outcomes<br>N = 120 women				the infants of the two groups."	
Patel 2024 [38]	Inclusion: babies born at GA < 32 weeks, being admitted immediately after birth<br>Exclusion: babies with congenital malformation, known chromosomal abnormality, severe perinatal asphyxia (Apgar score 0-3 for more than 5 min, a cord blood pH of less than 7 or both), or expected to die shortly after birth<br>N = 144 neonates (3 groups) (12 exclusions; N = 132 for analysis)	Group 3: 4 g IV bolus followed by 1 g/hour infusion until delivery or 24 hours whichever was earlier, and complete course antenatal steroid (N = 62 neonates)	Group 1: No steroids and no MgSO4 in antenatal period (N = 42 neonates)<br><br>Group 2: Complete and incomplete course of antenatal steroid, no MgSO4 (N = 40 neonates)	Pre-specified primary outcome(s): occurrence of IVH and/or PVL in the neonates during hospital stay<br>Pre-specified secondary outcome(s): occurrence of other morbidities like RDS, NEC, BPD, ROP, PDA, mortality<br>Other reported outcome(s): sepsis, mean ventilation days, mean CPAP days	"No intervention group (No antenatal steroids/ antenatal MgSo4) had highest chances of development of IVH/PVL. Significantly lower rate of IVH/PVL recorded in 'Steroid and MgSO4' group compared to only 'Steroid' group. The chances of development of RDS, BPD and NEC in no intervention group (No antenatal steroids and antenatal MgSo4) was significantly higher compared to steroid and and MgSO4 group and Steroid only group."	"It demonstrated that Antenatal steroids when used along with antenatal magnesium sulphate to mother expected to deliver preterm babies reduce IVH and White matter brain injury significantly than mothers receiving only antenatal steroids. It was also observed that antenatal steroids and MgSO4 combination offers more protection against NEC, and reduces mortality significantly."
Peng 2024 [39]	Inclusion: extremely preterm infants (< 28 weeks GA) admitted to the Department of Neonatology, Maternal and Child Health Hospital of Hubei Providence January 2013 to December 2022<br>Exclusion: repeated records of the same patient; incomplete records (patients still in hospital at time of manuscript submission); records of patients readmitted to the hospital for other diseases	MgSO4<br><br>Control (N = 94 neonates)<br>Transfer (N = 45 neonates)	No MgSO4<br><br>Control (N = 80 neonates)<br>Transfer (N = 64 neonates)	Reported outcome(s)*: received antenatal MgSO4 (neuroprotective measure)	"the proportions of… antenatal magnesium sulfate… were lower in the transfer group than in the control group (all P < 0.05)."<br>Transfer group: 41.3%<br>Control group: 54.0%	"EPIs born outside a provincial tertiary perinatal center and transferred postnatally do not have significantly higher mortality and rates of severe complications (severe PIVH, severe ROP, and sBPD), but there may be an increased risk of severe asphyxia, PIVH and

Study ID	Population characteristics	MgSO4 intervention/exposure	Non MgSO4 comparator	Outcome(s) specified/reported in relation to MgSO4*	Main results relevant to MgSO4	Main conclusion relevant to MgSO4
	after discharge (pneumonia, jaundice, anaemia, etc.)<br>N = 283 neonates (N = 174 control, N = 109 transfer)					EUGR. This may be caused by differences in maternal and neonatal characteristics (such as maternal disease, neonatal birth weight, and severe birth asphyxia) and management (such as antenatal medication and neonatal utilization of SRT)… Moreover, the perinatal management of EPIs, including cooperation between obstetricians and neonatal care, resuscitation in the delivery room, neuroprotection and nutritional support in the NICU, needs to be further improved in non-tertiary hospitals. Continuous quality improvement should be emphasized in all NICUs, with additional opportunities prior to high-risk mother-infant transfers”
Phrasidthideth 2021 [40]	Inclusion: preterm and low birthweight infants admitted to the hospital<br>Exclusion: NR	MgSO4 (N = NR)	No MgSO4 (N = NR)	Reported outcome(s)*: received MgSO4 (< 32 weeks)	“Patient care. Antenatal 5.9% of preterm births <32 weeks received magnesium sulfate.”	“Care can be improved, including antenatal administration of magnesium sulfate… that needs to be vastly

Study ID	Population characteristics	MgSO4 intervention/exposure	Non MgSO4 comparator	Outcome(s) specified/reported in relation to MgSO4*	Main results relevant to MgSO4	Main conclusion relevant to MgSO4
	N = 93 infants (including 86 preterm infants)					increased for eligible mother in preterm labor.”
Pirjani 2019 [41]	Inclusion: singleton pregnancy, spontaneous labour, 32-36 weeks GA, women 15-45 years<br>Exclusion: maternal underlying diseases, fetal abnormalities, intrauterine growth restriction, contraindications for use of MgSO4, iatrogenic preterm birth<br>N = 246 women	2 g/hour IV for 12 hours	No MgSO4 (receiving routine care including only control of labour progress and fetal heart rate monitoring)	Pre-specified primary outcome(s): neurodevelopmental status of infants (including Apgar score, arterial blood pH after the birth, and neurodevelopment status after the age of 4 months), measured with blood test and standard Ages & Stages Questionnaires (0, 4, 6, 8, 10, and 12 months after the birth)	NA	NA
Rauf 2017 [42]	Inclusion: maternal age 18-39 years, singleton pregnancies delivered before 32 weeks GA, MgSO4 group: treated with MgSO4 for fetal neuroprotection according to the standard protocol because of anticipated delivery within 24 hours<br>Exclusion: multiple pregnancies, fetal death, associated fatal congenital anomalies or chromosomal abnormalities, patients treated with MgSO4 for any indication during pregnancy period, patients who had contraindications for MgSO4 use<br>N = 107 women	6 g LD IV over 30 min, followed by 2 g/hour MD until delivery up to 12 hours) (N = 46 women)<br><br>Receipt of LD only was required to be considered as treated for fetal neuroprotection	No MgSO4 for fetal neuroprotection (N = 61 women)	Pre-specified other outcome(s): cranial ultrasound for IVH (within 7 days) and PVL (within first 4 weeks), ROP<br>Other reported outcome(s): neonatal outcomes of groups: birthweight, 1 min Apgar score, 5 min Apgar score, gender, need for active resuscitation at birth (respiratory support with endotracheal intubation), duration of hospitalisation in NICU, need for respiratory support, mechanical ventilation, nasal CPAP, nasal SIMV, oxygen hood, neonatal convulsion, neonatal hypotonia, neonatal	“Intraventricular hemorrhage was more common in control group compared with the MgSO4 group [7/61 (11.4%) versus 3/46 (6.5%); p=0.049]. For the periventricular leukomalacia [1 (2.2%) versus 0 (0%) respectively; p=0.430], neonatal convulsion [1 (2.2%) versus 3 (4.9%) respectively; P=0.630] and neonatal encephalopathy [0 (0%) versus 1 (1.6%) respectively; p=0.570], no substantial differences	“In conclusion, in this study, we demonstrated the effect of MgSO4 for fetal neuroprotection on maternal and neonatal outcomes of pregnants delivered before 32 weeks. The results of this study suggest that MgSO4 treatment for fetal neuroprotection has a beneficial effect on intraventricular hemorrhage rate, but there was no significant difference in IVH grade and periventricular leukomalacia rate between the groups. The widespread use of

Study ID	Population characteristics	MgSO4 intervention/exposure	Non MgSO4 comparator	Outcome(s) specified/reported in relation to MgSO4*	Main results relevant to MgSO4	Main conclusion relevant to MgSO4
				encephalopathy, neonatal death, major maternal adverse effects (death, respiratory arrest, cardiac arrest), minor adverse effects (nausea, vomiting, flushing, dry mouth, sweating, dizziness, blurred vision)	were seen between the groups."	prenatal MgSO4 for the purpose of fetal neuroprotection before 32 weeks of pregnancy at a standard dose protocol could improve the neonatal neurological outcomes."
Rivera-Rueda 2017 [43]	Inclusion: newborns < 1500 g admitted to the NICU of the Instituto Nacional de Perinatologia, Mexico (divided into those who survived and died)<br>Exclusion: NR<br>N = 135 neonates (divided into those who survived, N = 113 neonates, and those who died, N = 22 neonates)	MgSO4 (use as 'neuroprotective' from translation) (N = 52 neonates who survived; N = 4 neonates who died)	No MgSO4	Pre-specified outcome(s)*: received MgSO4, mortality	"The use of sulfate of magnesium applied in the mother as a neurological protector for the neonate was significantly higher in the patients who survived (p = 0.01)" (from translation)	"in our work there was a significant difference in the use of magnesium sulfate for those who survived. The work design is not enough to be able to observe neurological protection and death in the neonates" (from translation)
Sarath 2021 [44]	Inclusion: inborn babies 24 to 34 weeks GA<br>Exclusion: babies with congenital malformations and genetic syndromes<br>N = 70 infants	4 g IV LD over 30 min followed by 1 g/hour MD for 24 hours or until delivery, whichever occurred earlier (N = 70 infants)	Nil	Pre-specified primary outcome(s) (objective): serum magnesium (24 and 72 hours): hypermagnesemia; serum calcium: hypocalaemia<br>Pre-specified secondary outcome(s) (objective): serum magnesium in babies with and without neonatal morbidities: need for delivery room resuscitation, mechanical ventilation within 24 hours, hsPDA, need for inotropes within 24 hours, pulmonary haemorrhage,	"No baby had hyper-magnesemia. The mean cord blood magnesium level was 2.76 mg/dl which came down to 2.37 mg/dl by 24 hours of life. Babies who needed labour room resuscitation had slightly elevated cord blood magnesium."	"The results from our study indicate that hypermagnesemia does not pose a clinically significant problem, as none of the babies had clinical symptoms attributable to the same."

Study ID	Population characteristics	MgSO4 intervention/exposure	Non MgSO4 comparator	Outcome(s) specified/reported in relation to MgSO4*	Main results relevant to MgSO4	Main conclusion relevant to MgSO4
				hypocalcaemia, sepsis, NEC, abnormal neurosonograph findings (day 2, 7), mortality<br>Other pre-specified outcome(s):<br>Apgar scores at 1 and 5 mins, hypermagnesemia symptoms (apnoea, hypotonia, hyporeflexia, abdominal distention, delayed passage of meconium), RDS, ROP		
Sariati 2017 [45]	Inclusion: premature babies (with or without a history of antenatal MgSO4 therapy to the mother)<br>Exclusion: NR<br>N = 30 babies	IV MgSO4 (“minimal termination of pregnancy by cesarean is carried out more than 4 hours after the initial administration;” taken from translation) (N = 15 babies)<br><br>*Indication not clearly specified, however translated title suggests ‘neuroprotector of premature infants’*	No MgSO4 (N = 15 babies)	Pre-specified outcome(s): gross motor development - Denver II checklist according to the child’s age (normal (no delay or ≤ 1 alert), suspect (≥ 2 alerts and or ≥ 1 delay), untestable (≥ 2 delays)	"The research data is obtained as much as 43.3% of respondents with a history of antenatal magnesium sulphate diagnosed normal gross motor development, 6.7% suspect and no diagnosed untestable. While the respondents without a history of antenatal magnesium sulphate showed 16.7% of children diagnosed normal gross motor development and 33.3% suspect and no respondents who were diagnosed untestable. The results showed that there was a significant effect (p value = 0.008))”	“so that it can be concluded that a history of antenatal magnesium sulfate use in premature babies have a better effect than the group that did not have a history of use of antenatal magnesium sulfate.“
Sharma 2021 [46]	Inclusion: pregnant women with single or twin fetuses, < 32 weeks GA, with preterm labour, consent to participate	4 g LD IV over 20 min followed by 1 g/hour IV MD until birth (if occurred within 24	Placebo (sodium chloride solution/saline) (N = 300 women	Pre-specified outcome(s): (abstract and methods): procedure efficacy (incidence of neurological disabilities,	“Babies developing neurological disabilities were less in magnesium sulphate group than	“Although various studies have suggested that magnesium sulphate is cost

Study ID	Population characteristics	MgSO4 intervention/exposure	Non MgSO4 comparator	Outcome(s) specified/reported in relation to MgSO4*	Main results relevant to MgSO4	Main conclusion relevant to MgSO4
	Exclusion: women with GA more than 32 weeks, women in second stage of labour, history of receiving MgSO4 in this pregnancy (e.g. for eclampsia, hypertensive disease of pregnancy), contraindications to MgSO4 (RR <16/min, absent patellar reflex, urine output < 100 mL in previous 4 hours, renal failure, hypocalcaemia)<br>N = 600 women randomised; 586 women who received treatment as allocated	hours) or up to 24 hours (N = 300 women randomised, 294 received treatment)	randomised, 292 received treatment)	mortality and resuscitative measures), safety and side effects; maternal adverse effects, CUS within first 7 days (IVH) and beyond 4 weeks/at discharge (PVL)<br>Other reported outcome(s): complications due to intervention: headache, hypotension, nausea and vomiting, flushing and sweating, respiratory depression, hyporeflexia, palpitation, postpartum haemorrhage; characteristics at birth and neonatal morbidities: gestational age, birthweight, head circumference, total NICU admission, Apgar at 5 min < 7, intubation, external cardiac massage, epinephrine, NEC, IVH, fetal infection	sodium chloride group which is statistically insignificant. Total mortality in group A was 137 whereas in group B is 169 which is statistically insignificant. 1 neonate had intraventricular hemorrhage in group A while 4 in group B which is statistically insignificant."	effective and efficient neuroprotective agent in preterm babies but in our study we could not find significant difference between magnesium and placebo group , but it is proved to be efficient in preventing intraventricular hemorrhage and overall mortality. More study is needed to clarify the impact of magnesium on the cognitive outcome."
Sheeba 2022 [47]	Inclusion: expectant mothers, 24-34 weeks GA, if birth was expected within 24 hours<br>Exclusion: fetuses with severe malformations such as neural tube defects, and chromosomal abnormalities like trisomies, cases of maternal hypotension, renal insufficiency, hepatic insufficiency, and cardiac rhythm or electrolyte abnormalities	4 g IV bolus LD, followed by 1 g/hour IV MD for 24 hours or until birth, whichever came first (N = 45 women randomised, N = 40 analysed)	Placebo (IV normal saline) (N = 45 women randomized, N = 43 analysed)	Pre-specified primary outcome(s): neonatal death<br>Other pre-specified outcome(s): Amiel-Tison angles (for detecting abnormalities of tone at 6 months CA); Trivandrum Development Screening chart (to detect developmental delay, 6 months CA)	"MgSO4 use in mothers was not significantly associated with reduction in neonatal mortality (p-value=0.205). At six months of age, use of MgSO4 was associated with significant reduction in Amiel-Tison angle abnormalities (p-value <0.001), and reduction in developmental delay as	"Antenatal MgSO4 is a relatively inexpensive treatment. This study adds to the body of evidence supporting the role of MgSO4 in reducing the incidence of cerebral palsy in preterm infants and emphasises the need for large multicentric trials in India."

Study ID	Population characteristics	MgSO4 intervention/exposure	Non MgSO4 comparator	Outcome(s) specified/reported in relation to MgSO4*	Main results relevant to MgSO4	Main conclusion relevant to MgSO4
	N = 90 women randomised (somewhat unclear), 83 women/babies analysed				assessed by Trivandrum Development Screening Chart (TDSC) (p-value <0.001), showing that MgSO4 has a neuroprotective role."	"Although the percentage of neonatal deaths in the MgSO4 group were less, it was not statistically significant. Amiel-Tison angle abnormalities were significantly less in the group which received MgSO4. Neurodevelopmental outcome as assessed by TDSC was also significantly less in the group which received MgSO4."
Sulistyowati 2021 [48]	Inclusion: patients in the maternal ward or delivery room at Dr. Moewardi General Hospital, with inevitable preterm labour who agreed with consent; women aged 20-25 years, 28-34 weeks GA in threatened labour or with pregnancy termination for medical reasons<br>Exclusion: multiple pregnancies, intrauterine fetal death, fetuses with major and minor congenital abnormalities, pregnancies with severe renal insufficiency, and who refused to attend the study<br>N = 72 women	4 g IV LD followed by 1-2 g/hour IV MD until birth or for a maximum of 24 hours (N = 72 women)<br><br>*IV assumed*	Nil	Pre-specified outcomes: correlations between maternal serum magnesium levels/umbilical cord blood magnesium levels, and umbilical cord blood BDNF levels<br>Other reported outcomes: maternal and neonatal side effects ("did not aim to evaluate… did not receive any")	"The results showed there was a modest positive correlation between maternal serum magnesium levels to umbilical cord blood BDNF levels (p <0.05; r=0.367), and week positive correlation between umbilical cord blood magnesium levels to umbilical cord blood BDNF levels (p< 0.05; r=0.269). The positive correlation coefficient suggests that the increasing maternal magnesium blood levels and umbilical cord	"The antenatal MgSO4 for neuroprotection strategy in preterm birth gives impact on the increasing production of BDNF via higher maternal and fetal magnesium serum levels, and a higher dose is supposed to give higher production."

Study ID	Population characteristics	MgSO4 intervention/exposure	Non MgSO4 comparator	Outcome(s) specified/reported in relation to MgSO4*	Main results relevant to MgSO4	Main conclusion relevant to MgSO4
					magnesium levels will result in increasing BDNF levels."	
Tom 2018 [49]	Inclusion: pregnant women with single, twin and triplet fetus less than 33 weeks GA<br>Exclusion: not reported<br>N = 80 women/infants	4 g IV LD over 20-30 min followed by 1 g/hour IV MD for 4 hours (N = 25 women/infants)	No intervention (N = 39 women/infants)<br>(MgSO4/cord milking)<br>Cord milking (N = 14 women/infants)<br>Both interventions (N = 2 women/infants)	Pre-specified primary outcome(s): IVH (CUS day 28)<br>Other reported outcome(s): haemoglobin level, PCV levels	"The primary outcome showed only 1 infant who received magnesium sulphate developed IVH, whereas 3 infants with no intervention done developed IVH. None of the preterm infants who had undergone milking of umbilical cord developed IVH."	"We report that antenatal magnesium sulphate and milking of umbilical cord resulted in a significant reduction in the incidence of IVH. These low-cost interventions may be of immense importance in the improvement of neurologic outcome of preterm infants in resource limited countries and hence may be considered for the primary prevention of IVH in preterm infants born below 33 weeks of age" "Further studies using more number of samples are required to understand the role of the interventions for long-term implications."
Tummala 2024 [50]	Inclusion: all preterms between 26 to 33+6 weeks GA, multiple pregnancies (sample from 1st twin was taken), PPROM, women with pre-eclampsia who will be receiving neuroprophylaxis regimen	4 g IV LD over 20 min, followed by 6 g in 500 ml at 80 mL/hour (~1 g/hour IV MD) (N = 85 women)	Nil	Pre-specified outcome(s): cord blood magnesium level within 4-6 hours, unfavourable neonatal outcome: low Apgar, prolonged respiratory support required (reports: room air,	"The mean cord blood magnesium (n = 85) was 3.8 mg/dl. The AUROC for Gestational Age at Administration predicting Baby Outcome: 0.699, It was statistically	"Mean cord blood magnesium levels served as a tool to determine the timing and duration of Neuroprophylaxis. Mean cord blood

Study ID	Population characteristics	MgSO4 intervention/exposure	Non MgSO4 comparator	Outcome(s) specified/reported in relation to MgSO4*	Main results relevant to MgSO4	Main conclusion relevant to MgSO4
	Exclusion: H/O allergy for magnesium, renal failure or renal impairment in current pregnancy<br>N = 85 women	Few received bolus only, few bolus + infusion, duration of administration varied depending on time to delivery.		NCPAP/BCPAP, high frequency ventilation, intubated), baby succumbed (death) before discharge; ROC curve comparing cord blood magnesium levels and neonatal outcomes (and with GA)<br>Other reported outcome(s): birthweight, resuscitation (room air, NCPAP/BCPAP, high frequency ventilation, intubated)	significant (p = 0.034). The AUROC for Cord Blood Mg predicting Baby Outcome: 0.606, It was not statistically significant (p = 0.262).”	magnesium of 3.8 mg/dl should be achieved to serve the purpose of Neuroprotection. To achieve this, Bolus followed by Infusion should be administered for at-least 6 h prior to delivery."
Üstün 2021 [51]	Inclusion: all inborn infants ≤ 32 weeks admitted to the NICU during the study period<br>Exclusion: infants with major congenital anomalies, periviable infants who died within the first day of life<br>N = 340 infants	4 g IV LD followed by 2 g/hour IV MD; discontinued if delivery did not occur in 12 hours (N = 186 infants)	No MgSO4 (N = 154 infants)	Pre-specified primary outcome(s): acute cardiorespiratory events such as intubation at birth, respiratory support (invasive mechanical and nasal intermittent positive pressure, or CPAP), and hypotension within first day of life, hsPDA.<br>Pre-specified other outcome(s):<br>IVH, duration of invasive ventilation, age at full enteral feeds, NEC stage ≥ 2, moderate to severe BPD, ROP, length of NICU stay and mortality<br>Other reported outcome(s): IVH grade ≥ 3, PVL, sepsis (culture proven)	“Multivariate regression analysis showed that antenatal MgSO4 exposure was significantly associated with decreased mechanical ventilation (odds ratio [OR] 0.45 95% confidence interval [CI] 0.25-0.81, P=0.008), hypotension (OR 0.47, 95% CI 0.24-0.90, P=0.023) and HsPDA (OR 0.52, 95% CI 0.28-0.97, P=0.039). There was no significant association between antenatal MgSO4 exposure and intubation at birth (OR 1.06 95% CI 0.62-1.82, P=0.828).”	“The use of antenatal MgSO4 for neuroprotection was not associated with an increase in cardiorespiratory complications in preterm infants born ≤ 32 weeks of gestation. Moreover, infants exposed to antenatal MgSO4 had significantly less invasive mechanical ventilation, and hypotension treatment in the first day of life. Antenatal Mg appears to have a protective role in helping with ductal closure. Further studies with a larger population are needed to clarify the

Study ID	Population characteristics	MgSO4 intervention/exposure	Non MgSO4 comparator	Outcome(s) specified/reported in relation to MgSO4*	Main results relevant to MgSO4	Main conclusion relevant to MgSO4
						effect of antenatal MgSO4 on acute cardiorespiratory events and HsPDA.”
Vaz Ferreira 2024 [52]	Inclusion: VLBW infants ≤ 32 weeks GA and ≤ 1500 g, born at any of the 26 centres of the NEOCOSUR Neonatal Network<br>Exclusion: infants born < 24 weeks GA or < 500 g, as well as those born with any major congenital abnormality<br>N = 7418 infants	MgSO4 (infants with antenatal MgSO4 exposure) (N = 3399 infants)<br><br>*“we were not able to evaluate the administrated dose, or treatment timing, neither the reason for indicating it: for neuroprotection, as tocolytic or as part of severe preeclampsia syndrome treatment”*	No MgSO4 (infants not exposed) (N = 4019 infants)	Pre-specified primary outcome(s): death in delivery room, death after admission, severe IVH (grade III-IV, by CUS and according to Papile’s criteria), combined outcome grade III-IV IVH/death<br>Pre-specified secondary outcome(s): intubation, mask ventilation and cardiac massage in delivery room, 5 min Apgar score ≤ 3, surfactant, rate of mechanical ventilation, rate of PDA requiring treatment, NEC, surgical NEC, age at start of enteral feeds, age at 100 mL/kg/day of enteral feeds, PVL, late-onset sepsis, BPD, global ROP<br>Other reported outcomes: IVH global	“Antenatal MgSO4 was associated with a significantly decreased death rate after admission (aOR 0.67 [95% CI, 0.49–0.94]) and severe IVH/ death (aOR 0.68 [95% CI, 0.50–0.93]). No significant reduction in severe IVH was observed (aOR 1.11 [95% CI, 0.72–1.71]). No differences between groups were observed in rates of morbidities.”	“antenatal MgSO4 was associated with a decreased death rate after admission and combined severe IVH/ death in VLBW infants. However, these results must be interpreted with caution as they may represent an overall improved antenatal care in the MgSO4 exposed group.”
Wang 2024 [53]	Inclusion: preterm infants; cases = preterm infants with hearing loss; controls = preterm infants with normal hearing (matched 1:4 ratio according to GA and diagnosis of sepsis)<br>Exclusion: congenital brain/neural tube defects or chromosomal abnormalities; ear	Group 2: MgSO4 only (N = 22 infants)<br>Group 4: MgSO4 partial treatment (IM 1 dose of betamethasone combined with MgSO4) (N = 39 infants)	Group 1: no MgSO4 or betamethasone (N = 130 infants)<br>Group 3: partial treatment no MgSO4 (IM 1 dose of betamethasone) (N = 30 infants)	Pre-specified outcome(s): hearing loss, defined as preterm infants who failed both hearing screenings (initial hearing screening within 2-3 days after birth, those who failed were rescreened at 42 days after birth). Otoacoustic emission	"The antenatal use of magnesium sulfate had no effect on the hearing of preterm infants… Specifically, a single antenatal course (2-dose) of betamethasone had no effect on the hearing of preterm infants, but a	“In summary, this study revealed that antenatal betamethasone combined with magnesium sulfate treatment is a factor that affects the risk of hearing loss in preterm infants.”

Study ID	Population characteristics	MgSO4 intervention/exposure	Non MgSO4 comparator	Outcome(s) specified/reported in relation to MgSO4*	Main results relevant to MgSO4	Main conclusion relevant to MgSO4
	fistula/ear canal deformity; a family history of deafness; maxillofacial deformity; cCMV infection<br>N = 360 infants	Group 6: MgSO4 single course of treatment (IM 2 doses of betamethasone at 12-hour intervals combined with MgSO4) (N = 73 infants)<br>Group 8: multiple courses of treatment MgSO4 (IM > 2 doses of betamethasone combined with MgSO4) (N = 19 infants)	Group 5: single course of treatment, no MgSO4 (IM 2 doses of betamethasone at 12-hour intervals before delivery) (N = 35 infants)<br>Group 7: multiple courses of treatment, no MgSO4 (IM > 2 doses of betamethasone) (N = 12 infants)	test (OEA) or automated auditory brainstem response (AABR) used as the main diagnostic methods for hearing impairment	single antenatal course (2-dose) of betamethasone combined with magnesium sulfate treatment reduced the risk of hearing loss in preterm infants (OR = 0.21). Multiple courses (> 2 doses) of betamethasone had no effect on the hearing of preterm infants, but multiple courses (> 2 doses) of betamethasone combined with magnesium sulfate treatment increased the risk of hearing loss in preterm infants (OR = 4.46."	
Wu 2022 [54]	Inclusion: extremely preterm infants admitted to NICU (range reported 23+3 to 27+6 weeks GA)<br>Exclusion: NR<br>N = 185 infants (N = 140 infants in intervention period, N = 45 infants in historical period)	MgSO4 (N = 94 infants in intervention period, N = 22 infants in historical period)	No MgSO4 (N = 46 infants in intervention period, N = 23 infants in historical period)	Other reported outcome(s): received MgSO4; 1-minute Apgar score <= 3, endotracheal intubation in the delivery room or mechanical ventilation within 72 hours after birth; overall survival	"After the intervention, the proportion of patients in whom the neonatologists were involved in prenatal consultation, women who received… magnesium sulfate… increased significantly… 67.1% (94/140) vs 48.9% (22/45)… all P<0.05]… After the intervention, the overall survival rate of EPIs and that among those with gestational age from 27 to 27+6 were significantly improved"	NR (not clear from abstract)

Study ID	Population characteristics	MgSO4 intervention/exposure	Non MgSO4 comparator	Outcome(s) specified/reported in relation to MgSO4*	Main results relevant to MgSO4	Main conclusion relevant to MgSO4
Xiao 2024 [55]	Inclusion: all infants born of 24+0 to 31+6 weeks GA, inborn and admitted into NICU within 24 hours of life, data of each infant was complete, only included NICUs with average 50 very preterm infant admissions per year or more<br>Exclusion: infants with major congenital anomalies, infants without the results of head ultrasound<br>N = 24226 infants	MgSO4 (N = 13672 infants)	No MgSO4 (N = 10554 infants)	Pre-specified primary outcome(s): sIVH (equal to grade 3 Papile criteria) (versus non-sIVH (without IVH or grade 1 or grade 2 IVH)<br>Other pre-specified other outcome(s): treatment with eleven practices including MgSO4	"Regarding the eleven practices (Fig. 2A), … 56.4% (13,672/24,226) had antenatal MgSO4 therapy"<br>Figure 3 shows the risk-adjusted odds ratios of the care practice (MgSO4) for severe intraventricular haemorrhage after adjustment for the perinatal clinical Characteristics: OR 0.99 (95% CI 0.88 - 1.11); p=0.82	"The current evidence-based practices including the antenatal corticosteroids, MgSO4 therapy, and caffeine given within 3 DOL were not performed in each VPI as expected. Moreover, there were practical variations across multiple NICUs in China."<br>"we did not find the practices of the antenatal corticosteroids and MgSO4 therapy were associated with the lower rate of sIVH."
Yuce 2023 [56]	Inclusion: pregnant women hospitalised and received MgSO4 for preterm birth risk<br>Exclusion: women who gave birth before completion of 24 hours of MgSO4 administration, multiple pregnancies, patients with comorbid deteriorated liver or kidney functions, pre-eclampsia, intrauterine growth retardation, fetal abnormalities, gestational diabetes mellitus, chorioamnionitis, adolescent and advanced age pregnancies or any other obstetric complications were excluded from analyses<br>N = 31 women	MgSO4 – the fetal neuroprotective treatment protocol included 4 g IV LD over 30 minutes followed by 1 g/hour MD for 24 hours (N = 31 women)	Nil	Pre-specified primary outcome(s) [from aim]: maternal APRI score, calculated from the results of the biochemical analyses performed at initiation (basal) and 12th hour of MgSO4 administration<br>Pre-specified other outcome(s): hemogram and biochemical test results (magnesium, haemoglobin, haematocrit, platelet, AST)	"The mean APRI score at the 12th hour of administration (0.45±0.07) was significantly higher than the basal values (0.31±0.07) ($p<0.001$), but hemoglobin, hematocrit, and platelet values were similar ($p>0.05$ for all)."	"Magnesium sulfate treatment for preterm birth threat significantly increases APRI score at the 12th hour of administration."

Study ID	Population characteristics	MgSO4 intervention/exposure	Non MgSO4 comparator	Outcome(s) specified/reported in relation to MgSO4*	Main results relevant to MgSO4	Main conclusion relevant to MgSO4
Zhou 2023 [57]	Inclusion: preterm born group: born at ≥ 28 weeks and < 32 weeks GA, Apgar score > 7 at 5 min, transferred to the NICU for treatment within 12 hours after birth, normal results of CUS or MRI; complete obstetrics and neonatal paediatrics clinical data available, right-hand dominance, Chinese as the primary language, and good compliance<br>Term born group: born at ≥ 37 and < 42 weeks GA, birth weight > 2500 and < 4000 g; Apgar score > 7 at 5 min, MgSO4 not used during pregnancy, right-hand dominance, Chinese as the primary language, and intelligence scale and MRI scan completed between the ages of 10 and 16 years<br>Exclusion (all groups): complications during pregnancy such as pregnancy-induced hypertension, heart disease, thyroid disease, haematological diseases, multiple gestation, chorioamnionitis, PPROM, fetal growth restriction, fetal distress, and other diseases; long-term medication use by the mother during pregnancy because of chronic disease; smoking, alcohol consumption, or illegal drug use by the mother during pregnancy; mental illness experienced by the	MgSO4 (PTB)<br>4 g IV LD over 30 minutes reduced to 1 g/hour IV MD – lasting for 1-6 days (N = 17 children)<br><br>*Indication not clearly specified, however paper suggests neuroprotection focus*	No MgSO4 (PTB) (N = 11 children)<br>No MgSO4 (TB) (N = 23 children)	Pre-specified primary outcome [from aim] grey matter morphology/GMV [MRI])<br>Other pre-specified outcome(s): IQ - full scale, verbal, performance score (C-WISC)	"Verbal and full-scale intelligence quotient scores were significantly lower for PTB children without magnesium than for TB children; however, the scores of PTB children with magnesium and TB children were almost identical."<br>"The influence of magnesium sulfate treatment was not significant, but the cognitive levels of these children were significantly increased and almost identical to those of TB children. Initiation of magnesium sulfate treatment during gestation is negatively correlated with the left inferior frontal gyrus GMV."	"The influence of MgSO4 treatment on cerebral structures is not significant; however, the cognitive level of the subjects increased substantially."

Study ID	Population characteristics	MgSO4 intervention/exposure	Non MgSO4 comparator	Outcome(s) specified/reported in relation to MgSO4*	Main results relevant to MgSO4	Main conclusion relevant to MgSO4
	mother during pregnancy; newborns with congenital malformations of important organs, genetic or metabolic diseases, chromosomal abnormalities, nervous system diseases, retinopathy of preterm infants, congenital or acquired severe infection, hypoglycaemia, and other diseases or treatment including mechanical ventilation or blood transfusion in the NICU; severe head trauma; serious diseases or disabilities of important organs and limbs; and abnormal brain structures found with MRI during the structural phase.<br>N = 51 children					

**Additional outcomes (not assessed in relation to effects of MgSO4) reported within the study*

Abbreviations: ABS: auditory brain stem; ACS: antenatal corticosteroids; ACTOMgSO4: Australasian Collaborative Trial of Magnesium Sulphate; APRI: Aspartate Aminotransferase to Platelet Ratio Index; BDP: bronchopulmonary dysplasia; BDNF: brain-derived neurotrophic factor; BEAM: Beneficial Effects of Antenatal Magnesium; BP: blood pressure; CA; corrected age; cCMV: congenital cytomegalovirus; CLD: chronic lung disease; cm: centimetres; CPAP: continuous positive airway pressure; CUS: cranial ultrasound; C-WISC: Chinese Wechsler Intelligence Scale for Children; g: grams; DASII: Developmental Assessment Scale for Indian Infants; DIC: Disseminated intravascular coagulation; dL: decilitre; EEG; electroencephalopathy; FHR: fetal heart rate; FOGSI: Federation of Obstetric and Gynaecological Societies of India; g: grams; GA: gestational age; GMV: gray matter volume; HR: heart rate; hsPDA: haemodynamically significant patent ductus arteriosus; ICU: intensive care unit; IM: intramuscular; IQ: intelligence quotient; IV: intravenous; IVH: intraventricular haemorrhage; LD: loading dose; MAGENTA: Magnesium Sulphate at 30 to 34 Weeks' Gestational Age; MagNET: Magnesium and Neurologic Endpoints Trial; MASP: Magnesium Sulphate for Preterm Birth; MCA: middle cerebral artery; MD: maintenance dose; mg: milligrams; MgSO4: magnesium sulphate; min: minutes; mL: millilitres; MRI: magnetic resonance imaging; NA: not applicable; NEC: necrotising enterocolitis; NICU: neonatal intensive care unit; NR: not reported; NT: normal body temperature; PDA: patent ductus arteriosus; pH: potential of hydrogen; PI: pulsatility index; PPROM: preterm premature rupture of membranes; PREMAG: PREterm brain protection by MAGnesium sulphate; PTB: preterm born; PVL: periventricular leukomalacia; RDS: respiratory distress syndrome; RCT: randomised controlled trial; RI: resistance index; ROP: retinopathy of prematurity; RR: respiratory rate; SIP: spontaneous intestinal perforation; SIMW: synchronized intermittent mandatory ventilation; SMA: superior mesenteric artery; SNCU: special newborn care unit; S/D: systole/diastole ratio; TB: term born; UA: uterine artery; UMA: umbilical artery; VLBW: very low birthweight; WHO: World Health Organization

References (Included Research Studies)

1. Abdel Fattah G. NCT02506894: Fetal Middle Cerebral Artery Doppler in Preterm Births Receiving Magnesium Sulfate for Neuroprotection 2015 [cited 4 December 2023]. Available from: https://clinicaltrials.gov/study/NCT02506894.
2. Achola KA, Lester F, Lihanda P, Olack B, Otare C, Miller L, et al. Adapting the WHO Safe Childbirth Checklist for preterm birth in Migori County, Western Kenya Int J Gynaecol Obstet. 2018;143(S3):158.
3. Atia H. NCT05674565: Magnesium Sulphate Neuroprotective Strategies for Preterm Deliveries 2023 [cited 9 November 2023]. Available from: https://clinicaltrials.gov/study/NCT05674565.
4. Bachnas MA, Sulistyowati S, Akbar U. Dose dependency in antenatal magnesium sulphate to promote brain-derived neurotrophic factor production. Ultrasound Obstet Gynecol. 2020;56(Suppl 1):147.
5. Bachnas MA, Sulistyowati S, Akbar U. Correlation between antenatal magnesium sulfate (MgSO4) total dose and delivery time interval with umbilical cord blood brain-derived neurotrophic factor (BDNF) levels as a neuroprotection strategy in preterm birth. Bali Med J. 2022;11(1):293-8.
6. Bansal V, Desai A. Efficacy of Antenatal Magnesium Sulfate for Neuroprotection in Extreme Prematurity: A Comparative Observational Study. J Obstet Gynaecol India. 2022;72(Suppl 1):36-47.
7. Cavalcanti RM, Sciamareli N, Campos F, Morato R, Souza AC, Silva R, et al. Tips and challenges to implement good childbirth care practices at an university hospital in Brazil. Int J Gynaecol Obstet. 2018;143(Suppl 3):158.
8. Chandran S, Tergestina M, Ross B, Joshi A, Rebekah G, Kumar M. Effects of Antenatal Magnesium Sulfate on the Gut Function of Preterm (<32 weeks) Very Low Birth Weight Neonates: Experience from a Tertiary Institute in South India. J Trop Pediatr. 2021;67(2):1-9.
9. Daneji SM, Kasim NI, Takai IU. Clinical audit of the management and outcomes of preterm pre-labour rupture of membranes at Aminu Kano Teaching Hospital. Pan Afr Med J. 2022;43:41.
10. Diggikar S, Kulkarni S, Aradhya AS, Venkatagiri P. Use of antenatal magnesium sulfate for neuroprotection: a survey of national practices. Int J Contemp Pediatrics. 2021;8(4):744-6.
11. Dolly M, Mammen MV. Retrospective Study of Neonatal Outcomes with the Use of Magnesium Sulfate in Case of Preterm Labor. Int J Res Publ Rev. 2022;3(10):886-94.
12. Gathwala G. CTRI/2018/06/014386: Antenatal magnesium sulphate for neuroprotection in preterm infants: An open label randomized control trial 2018 [cited 4 December 2023]. Available from: http://www.ctri.nic.in/Clinicaltrials/pmaindet2.php?trialid=22360.
13. Gupta N, Garg R, Gupta A, Mishra S. Magnesium Sulfate for Fetal Neuroprotection in Women at Risk of PretermBirth: Analysis of its Effect on Cerebral Palsy. J South Asian Fed Obstet Gynecol. 2021;13(3):90-3.
14. Gupta N, Jahan U, Seep S, Singh CP, Shukla S. Role of magnesium sulphate as a neuroprotective agent on neonatal outcome in preterm deliveries. Int J Reprod Contracept Obstet Gynecol. 2023;12(3):658-64.
15. Iqbal M, Muhammad Z, Iqbal S. Role of magnesium sulfate for fetal neuroprotection in women at risk of preterm birth. J Peoples U Med Health Sci. 2023;13(1):124-8.
16. Jamileh S, Masoumeh S, Nezamaddin E, Jalileh S, Gheshlagh Reza G, Sedigheh B, et al. Effect of magnesium sulfate on middle cerebral, umbilical artery and uterine arteries Doppler parameters in pregnancy up to 32 weeks. Med Sci. 2018;22(94):549-52.
17. Jayashree V, Vanathi R, Athmika N. Effect of magnesium sulphate for antenatal mothers in preterm labour for neuroprotection in infants. Int J Clin Obstet Gynaecol. 2021;6(6):243-9.
18. Jin D, Gu X, Jiang S, Wang Y, Yang T, Lu Y, et al. The Association between Evidence-Based Healthcare Practices and Outcomes among Preterm Births in China. Neonatology. 2022;119(1):26-32.
19. Kasapoglu I, Cetinkaya Demir B, Atalay MA, Orhan A, Ozkan H, Cakir SC, et al. Does antenatal magnesium sulphate improve hearing function in premature newborns? J Turk Ger Gynecol Assoc. 2020;21(3):187-92.
20. Kumar N, Kumar P, Kumar S, Tulsiyan P. Antenatal Low Dose Magnesium Sulphate for Foetal Neuroprotection in Preterm Birth Versus Control: A Comparative Prospective Cohort Study in Tertiary Care Centre in India. J Obstet Gynaecol India. 2025;75(1):46-52.
21. Mamatha S, Ananda M, Shaziya S, Manjunatha S, Somashekar HK. Maternal and Fetal outcome in usage of magnesium sulphate for fetal neuroprotection in pregnancy less than 34 weeks of gestation. Int J Life Sci Biotechnol Pharma Res. 2023;12(1):488-97.
22. Manoj Varanattu C, Menon B, Sankar A, Anand M, Manikoth P. Randomized controlled trial of antenatal magnesium sulfate for short-term neuroprotection in premature neonates. Indian J Child Health. 2017;4(2):199-202.
23. Marnal A, Poornima M, Suma KB, Sahana K. MgSO4 for neuroprophylaxis in preterm birth - clinical audit-re audit. Dig J Clin Med. 2023;5(4):121-3.
24. Mazhar T, Rauf S, Ambareen A, Nadir S. The efficacy and safety of magnesium sulphate for neonatal neuroprotection in patients with imminent preterm deliveries: experience at a tertiary care hospital. J Med Sci. 2023;31(2):137-42.

25. Medhi R, Das I, Boro RC, Naznin W. Fetomaternal outcome in patients with early preterm labour following administration of magnesium sulphate-a hospital based prospective study. New Indian J OBGYN. 2023;10(1):39-45.
26. Millogo-Traoré TFD, Sawadogo O, Zongo-Kondé SW. Evaluation of the Fetal Neuroprotection Protocol with Magnesium Sulphate in a University Hospital in Burkina Faso. Open J Obstet Gynecol. 2022;12:1042-55.
27. Mohan RJ, Lakshmy S, Fiji. The Effect of Antenatal Magnesium Sulphate for Fetal Neuroprotection in Threatened Preterm Labour: A Prospective Cohort Study. Int J Innov Sci Res Technol. 2023;8(9):837-93.
28. Muhammed HO, Murad SJ. Effect of Prenatal Infusion of Magnesium Sulphate on Neurological Complication in Preterm Infant. Int J Pharm Res. 2019;11(3):29-34.
29. Musiime G, Lloyd L, McCaul M, Van Zyl N, Holgate S. Outcomes of extremely low-birthweight neonates at a tertiary hospital in the Western Cape, South Africa: A retrospective cohort study. S Afr J Child Health. 2021;15(3):170-5.
30. Nunes RD, Schutz FD, Traebert JL. Association between the use of magnesium sulfate as neuroprotector in prematurity and the neonatal hemodynamic effects. J Matern Fetal Neonatal Med. 2018;31(14):1900-5.
31. Okulu E, Kraja E, Kostekci YE, Seker E, Ozisik MS, Sarisoy D, et al. Effect of Antenatal Magnesium Sulfate Exposure on Patent Ductus Arteriosus in Premature Infants. Am J Perinatol. 2024;41(12):1665-72.
32. Ozer Bekmez B, Oguz Y, Kutman HGK, Uygur D, Canpolat FE, Oguz SS, et al. The Effect of Antenatal Neuroprotective Magnesium Sulfate Treatment on Cerebral Oxygenation in Preterm Infants. Am J Perinatol. 2021;38(S 01):e64-e70.
33. Ozer Bekmez B, Kanmaz Kutman HG, Oguz Y, Uygur D, Elbayiyev S, Canpolat FE, et al. Antenatal Neuroprotective Magnesium Sulfate in Very Preterm Infants and Its Association With Feeding Intolerance. J Pediatr Gastroenterol Nutr. 2023;77(5):597-602.
34. Özlü F, Hacıoğlu C, Büyükkurt S, Yapıcıoğlu H, Satar M. Changes on preterm morbidities with antenatal magnesium. Çukurova Med J. 2019;44(2):502-8.
35. Padmapriya R, Niveditha L, Sukanya L, Saraswathi N. Role of antenatal magnesium sulphate as a fetal neuroprotection in preterm labour. Int J Acad Med Pharm. 2023;5(4):699-703.
36. Pandey D, Arja K, Inukollu PR, Kuppili M. The role of magnesium sulphate (MgSO4) in fetal neuroprotection. J Cardiovasc Dis Res. 2024;15(8):122-8.
37. Parashi S, Bordbar A, Mahmoodi Y, Jafari MR. The survey of magnesium sulfate in prevention of intraventricular hemorrhage in premature infants: a randomized clinical trial. Shiraz E Med J. 2017;18(11):e55094.
38. Patel D, Vora A, Goyal P, Dubey P. Effects of Antenatal Steroids with Magnesium Sulphate on Intraventricular Hemorrhage and Periventricular Leukomalacia in Neonates Born below 32 Weeks of Gestation. Nat J Med Res. 2024;14(1):4-10.
39. Peng S, He X, Xia S. Extremely preterm infants born outside a provincial tertiary perinatal center and transferred postnatally associated with poor outcomes: a real-world observational study. Front Pediatr. 2024;12:1287232.
40. Phrasidthideth B, Phengsavanh A, Kiatchoosakun P. Evaluation of preterm and low birth weight morbidity, mortality and standards of care in Lao PDR. Lao Med J. 2021:31-40.
41. Pirjani R, Zndi S. IRCT20120826010664N5: Evaluation of neuroprotective effects of magnesium sulfate on preterm infants with a gestational age of 32-36 weeks 2019 [cited 6 December 2023]. Available from: http://en.irct.ir/trial/41934.
42. Rauf M, Sevil E, Ebru C, Yavuz S, Cemil C. Antenatal magnesium sulfate use for fetal neuroprotection: experience from a tertiary care hospital in Turkey. Biomed Res - India 2017;28(4):1749-54.
43. Rivera-Rueda M, Fernández-Carrocera L, Michel-Macías C, Carrera-Muiños S, Arroyo-Cabrales L, Coronado-Zarco I, et al. [Morbilidad y mortalidad de neonatos< 1,500 g ingresados a la UCIN de un hospital de tercer nivel de atención]. Perinatol Reprod Hum. 2017;31(4):163-9.
44. Sarath MB, Anand MR, Remesh P, Nath D, Mohan V. Postnatal Magnesium Trend in less than 34 week Preterms who received Antenatal Neuroprotective Doses of Magnesium Sulphate. Fetus Newborn. 2021;1(2):34–7.
45. Sariati Y, Nooryanto M, Anggraini PDA. Pengaruh Penggunaan Magnesium Sulfate (MgSO4) Saat Antenatal Sebagai Neuroprotektor Bayi Prematur Terhadap Perkembangan Motorik Kasar Usia 2-3 Tahun. J Issues Midwifery. 2017;1(2):50-7.
46. Sharma K, Ranideepa, Anamika. To study the effect of antenatal magnesium sulphate for neuroprotection in preterm babies. Int J Adv Res. 2021;9(4):628-33.
47. Sheeba LM, Namboodiripad A, Varanattu MC. Efficacy of Maternal Magnesium Sulfate Administration on the Neurodevelopmental Outcome of Preterm Babies: A Randomised Controlled Trial. J Clin Diagn Res. 2022;16(12):SC06-SC9.
48. Sulistyowati S, Bachnas MA, Ekasari S, Wijayanti AS. Correlation of the Magnesium Serum Levels in Maternal and Fetal over the Fetal Brain-Derived Neurotrophic Factor (BDNF) after Antenatal Magnesium

Sulphate (MgSO4) Provision in the Preterm Birth Neuroprotection Strategy. Syst Rev Pharm. 2021;12(1):1624-9.
49. Tom SM, Manoj VC. Effect of antenatal administration of magnesium sulphate and milking of umbilical cord during delivery on the incidence of intraventricular haemorrhage in preterm infants. Int J Contemp Pediatrics. 2018;5(5):1943-6.
50. Tummala M, Upadhya R, Pai MV. Timing of administration of antenatal magnesium sulphate and correlation with umbilical cord blood magnesium levels in preterm babies. Eur J Obstet Gynecol Reprod Biol. 2024;297:197-201.
51. Üstün N, Hocaoğlu M, Turgut A, Ovalı F. Effects of antenatal magnesium sulfate use for neuroprotection on cardiorespiratory complications during the early neonatal period in preterm infants. J Surg Med. 2021;5(9):843-7.
52. Vaz Ferreira C, Caro J, Villarroel L, Munoz S, Alvarez P, Flores G, et al. Antenatal exposure to magnesium sulfate and neonatal outcomes in very low birth weight infants: a multicenter study. J Perinatol. 2024;44(11):1663-8.
53. Wang Y, Chang J, Huang Y. Effects of different doses of antenatal betamethasone alone or in combination with magnesium sulfate exposure on hearing in preterm infants: A retrospective case–control study. Research Square [Preprint]. https://doi.org/10.21203/rs.3.rs-4397672/v1]. 2024 [updated 21 May 2024 cited 4 March 2025]. Available from: https://www.researchsquare.com/article/rs-4397672/v1.
54. Wu Y, Zhong X, Zhou L, Gong H. Effects of intervention program from prenatal period to delivery room on outcomes of extremely preterm infants. Chin J Perinat Med. 2022;4:263-70.
55. Xiao T, Hu L, Chen H, Gu X, Zhou J, Zhu Y, et al. The performance of the practices associated with the occurrence of severe intraventricular hemorrhage in the very premature infants: data analysis from the Chinese neonatal network. BMC Pediatr. 2024;24(1):394.
56. Yüce E. Does MgSO4 Treatment Affect Maternal Aspartate Aminotransferase to Platelet Ratio Index (APRI) Score in Preterm Labor? Med Rec. 2023;5(3):644-7.
57. Zhou L, Liu X, Yan X, Liu Y, Xie Y, Sun C. Long-term effects of prenatal magnesium sulfate exposure on nervous system development in preterm-born children. Food Sci Nutr. 2023;11(11):7061-9.
58. Niveditha M. Role of Antenatal Magnesium Sulphate as a Fetal Neuroprotection in Preterm Babies [MS (Obstetrics and Gyanecology) thesis]: Coimbatore Medical College, Coimbatore; 2020.

S5 Table. Characteristics of guidelines (1)

Guideline ID	Author/ Developer	Language	Country: World Bank Classificatio n	Guideline type: scope	Document length	Methodology	Supporting MgSO4 evidence cited
**International**							
FIGO 2021 [1]	FIGO Working Group for Preterm Birth	English	International	Specific	3 pages (journal article)	Methods not described in detail; authors and FIGO Working Group for Preterm Birth drafted the concept and idea, with authors writing the manuscript, and all authors and working group members approving the final version. Described as 'good practice recommendations'	In-text/recommendation citations<br>RCTs: Crowther 2003, Marret 2007<br>SRs: Conde-Agudelo 2009, Doyle 2009, Crowther 2017, Shepherd 2019
FIGO 2023 [2]	FIGO Childbirth and Postpartum Haemorrhage Committee	English	International	General: preterm labour and PPROM	11 pages (journal article)	Methods not described in detail; article provides "summary of evidence-based management of preterm labor and PPROM."<br>Described as 'good practice recommendations.'	In-text/recommendation citations<br>Guidelines: ACOG 2016b, FIGO 2021, NICE 2015, SOGC 2019, WHO 2015<br>SR: Bain 2012, Conde-Agudelo 2009, Costantine 2009, Doyle 2009
ISUOG 2020 [3]	ISUOG	English	International	General: diagnosis and management of small-for-gestational-age fetus and fetal growth restriction	15 pages (journal article)	Methods not described in detail, although details of 'grades of recommendation' are provided. Described as 'practice guidelines'	In-text/recommendation citations:<br>Guidelines: ACOG 2019, IOG 2017, UoA 2010, Vayssiere 2015<br>SR: Wolf 2020<br>Other: Stockley 2018, Ting 2018

Guideline ID	Author/ Developer	Language	Country: World Bank Classification	Guideline type: scope	Document length	Methodology	Supporting MgSO4 evidence cited
WAPM & PMF 2023 [4]	WAPM & PMF	English	International	General: management of preterm labour	10 pages (journal article)	Provides summary of guideline methods: Delphi consensus methodology (panel of international experts), informed by a thorough scoping literature review, with the use of GRADE/WHO guidance to provide strength and level of evidence. Described as 'clinical practice guideline and recommendation'	In-text/recommendation citations:<br>SR: Doyle 2009, Shepherd 2019, Tsakiridis 2020<br>RCTs: Altman 2002, Crowther 2023
WHO 2017 [5]	WHO	English	International	General: maternal health	36 pages	Summary of guideline process provided, with reference to 'WHO Handbook for guideline development' – includes synthesis and assessment of quality of evidence (based on GRADE approach), the formulation of recommendations by an expert group using evidence to decision framework, and decisions on strength of the recommendations. Described as 'recommendations' and 'guidelines'	In-text/recommendation citation:<br>Guideline: WHO 2015
**National/multi-national**							
FASGO 2022 [6]	FASGO	Spanish	Argentina: Upper-middle income	General: premature ROM	18 pages	Methods not described; a 'consulted bibliography' is provided at the end of the document. This is an	General reference list<br>Not cited in-text; potentially relevant citations:

Guideline ID	Author/ Developer	Language	Country: World Bank Classification	Guideline type: scope	Document length	Methodology	Supporting MgSO4 evidence cited
						update to the 2018 consensus. One listed goal is to "Establish the existing controversies on the subject that require further research and scientific evidence for its final recommendation". Described as 'FASGO CONSENSUS'	SR: Crowther 2017<br>Guidelines: NICE 2015, RCOG 2019, ACOG 2016, ACOG 2018
FECASOG 2018 [7]	FECASOG (technical execution by IHCAI)	Spanish	Central America:<br><br>Costa Rica, Dominican Republic, Guatemala: upper-middle income<br><br>Honduras, Nicaragua: lower-middle income<br><br>[Panama: high-income]	General: preterm labour management	195 pages	Methods described in detail, provides a list of guidelines selected for methodological adaptation, and the use of the ADAPT guidance. Described as a 'clinical practice guideline'	<u>General reference list (adapted guidelines)</u><br>Guidelines: ACOG 2016, NICE 2015, WHO 2015<br><br><u>In-text/recommendation citation</u><br>SR: Doyle 2009
FOGSI 2017 [8]	FOGSI	English	India: Lower-middle income	General: preterm labour	74 pages	Methods not described; a 'literature review' of guidelines in preterm labour provided at the end of the document. Provided in a list of 'FOGSI & ICOG Good Clinical Practice Recommendations'	<u>General reference list</u><br>Guidelines: ACOG 2016, NICE 2015, WHO 2015<br><br><u>In-text/recommendation citation</u><br>Guideline: SOGC 2011

Guideline ID	Author/ Developer	Language	Country: World Bank Classificatio n	Guideline type: scope	Document length	Methodology	Supporting MgSO4 evidence cited
Gov Nepal MoH 2022 [9]	Gov Nepal, MoH and Population, Department of Health Services, Family Welfare Division	English	Nepal: Lower-middle income	General: maternal and newborn care	330 pages	Detailed methods not provided in detail, however a summary of the revision process was provided "align[ed] with WHO's normative function a rigorous approach to revising standards [was] followed", which included a technical working group, literature review and analysis, consultative meetings/workshops. Described as 'national medical standard'	General reference list<br>Not cited in-text/chapter; potentially relevant citations:<br>Guidelines: ACOG 2016, WHO 2016, WHO 2017b
Gov Pakistan 2023 [10]	Gov Pakistan, Ministry of National Health Services, Regulation & Coordination (supported by UNF)	English	Pakistan: Lower-middle income	General: emergency obstetric and newborn care	59 pages	Methods not provided in detail, however a summary of the systematic process to update the clinical protocols provided; this included establishment of multidisciplinary working group, a comprehensive literature review, gaps/areas for improvement identified, local context/resources identified, stakeholders and experts engaged, protocols drafted, and reviewed/validated. Described as 'Clinical protocols and guidelines'	Nil; methods described a comprehensive literature review: "Relevant studies, systematic reviews, clinical trials, and guidelines published by reputable organizations and institutions were identified"

Guideline ID	Author/ Developer	Language	Country: World Bank Classificatio n	Guideline type: scope	Document length	Methodology	Supporting MgSO4 evidence cited
INPer 2021 [11]	INPer	Spanish	Mexico: Upper-middle income	General: obstetric standards and procedures	338 pages	Methods not provided; literature/references provided at the end of each chapter. Described as 'standards and procedures'	General reference list (chapter)<br>Guidelines: ACOG 2010, SOGC 2019<br>SRs: Galinsky 2020, Shepherd 2017, Wolf 2020<br>Other: Chollat 2018,<br><br>Refers to 'several observational studies… several randomized clinical trials…"; 'The MAGENTA trial' and guidelines from: ANCP, CNGOF, KCE, NICE, WHO, RCOG, SOGC in text/tables without citations provided
MoH Kenya 2024 [12]	MoH [Kenya]	English	Kenya: Lower-middle income	General: obstetrics and perinatal care	836 pages	Methods described, including desk review and scoping of the existing national guideline (2012); search per topic for guidelines guided by the ADAPTE process; with further review of the guideline by stakeholders (policy makers, professionals, academia, researchers, implementer of services, and community at technical workshops), with external review using the Delphi technique, a final version	In-text/recommendation citations<br>Guideline: SA 2015, QLD 2016, SOGC 2011 [citation should be provided for WHO 2015, Rundell 2017 in error, as provides WHO 2015 summary table of recommendations as an image]

Guideline ID	Author/ Developer	Language	Country: World Bank Classification	Guideline type: scope	Document length	Methodology	Supporting MgSO4 evidence cited
						and validation meeting. Described as 'national guidelines'	
MoH Malaysia 2023 [13]	MoH [Malaysia], JPPOBG	English	Malaysia: Upper-middle income	General: prevention and management of preterm birth	39 pages	Methods not detailed; "based on evidence obtained from published scientific literature, international guidelines and the experience of the guideline committee members"; described as 'guidelines'	In-text/recommendation citations<br>Guidelines: NICE 2015<br>SR: Doyle 2009
MoH Nicaragua 2022 [14]	MoH Nicaragua	Spanish	Nicaragua: Lower-middle income	General: obstetric complications	321 pages	Methods not described in detail; "based on the latest available scientific evidence"	No specific in-text citations provided<br><br>General reference list (relevant sections)<br>Not cited in-text/chapter; potentially relevant guidelines:<br>Placenta praevia: INPer 2021<br>PROM: ACOG 2016b, WHO 2017b<br>RDS: Di Renzo 2017, Sweet 2016
MoH Palestine 2024 [15]	MoH of Palestine, Women's Health and Development Unit	English	West Bank and Gaza: lower-middle income	General: obstetric guidelines and labour ward protocols	236 pages	Methods not described; "extensive national collaboration among expert obstetricians, and midwives from the West Bank and Gaza strip", with a National expert committee who updated the guidelines named. Described as 'Guidelines	General reference list<br>Guideline: RCOG 2019

Guideline ID	Author/ Developer	Language	Country: World Bank Classification	Guideline type: scope	Document length	Methodology	Supporting MgSO4 evidence cited
						and Labor Ward Protocols'	
OGSB 2019 [16]	OGSB	English	Bangladesh: Lower-middle income	General: emergency obstetric and neonatal care	136 pages	Process for protocol development provided – involving core committee, working group committee, EC members of OGSB, relevant experts of medical colleges and other experienced persons (series of expert consultation meetings); with protocols developed "taking help of literature review and evidence based practices"; "updated using international guideline e.g. WHO, NICE, & RCOG guideline with references". Described as 'standard clinical management protocols and flowcharts'	General reference list (for section)<br>Guideline: WHO 2017<br><br>Also, in methods text, describes use of "international guidelines e.g. WHO, NICE & RCOG"
OGS MMA 2024 [17]	OGS, MMA	English	Myanmar Lower-middle income	General: obstetrics and gynaecology management	269 pages	Methods not described in detail; "Management Guidelines are systematically developed recommendations… Guideline Review and Constitution Committee and Editorial committee were formed with senior members of Myanmar Obstetrical and Gynaecological society in 2019 to update the	Nil

Guideline ID	Author/ Developer	Language	Country: World Bank Classification	Guideline type: scope	Document length	Methodology	Supporting MgSO4 evidence cited
						management guidelines…"Described as 'management guidelines'	
PNG O&G Society 2018 [18]	Glen DL Mola, President of PNG O&G Society	English	Papua New Guinea: Lower-middle income	General: management in obstetrics and gynaecology	258 pages	Detailed methods not provided (PNG O&G Society, with input from WHO). Described as manual of statement managements for doctors, HEOs and nurses in PNG	Nil
POGI 2019 [19]	POGI, HKFM Indonesia, Dinas Kesehatan Indonesia	Indonesian	Indonesia: Upper-middle income	General: preterm labour/birth	87 pages	Methods described include a literature search, assessment and critical review of literature, evidence rating (level of evidence), and degree of recommendations. Described as 'guide' and 'guidelines'	In-text/recommendation citations<br>Guidelines: ACOG 2010, NICE 2015, SOGC 2011<br>SR: Conde-Agudelo 2009<br>RCT: Crowther [in Table], Marret [in Table], Rouse 2008
PSNbM 2019 [20]	PSNbM Consensus Working Group	English	Philippines: Lower-middle income	General: decision-making around resuscitation of extremely preterm infants	6 pages (journal article)	Consensus development methods described ("Where relevant, evidence from randomised controlled trials and systematic reviews was used as the basis for practice"). Described as a 'consensus guideline'	In-text/recommendation citations:<br>Guideline: WHO 2015<br>SR: Doyle 2009
PUDER 2020 [21]	PUDER	English	Turkey: Upper-middle income	General: preterm labour and delivery	13 pages (journal article)	Methods not provided. Described as a 'guideline'	In-text/recommendation citation<br>Guideline: ACOG 2013
SASOG 2023a [22]	SASOG and Better Obs	English	South Africa: Upper-middle income	General: preterm labour with	8 pages	Methods not provided in detail ("drafted by a clinical team… reviewed by a panel of experts";	Nil

Guideline ID	Author/ Developer	Language	Country: World Bank Classification	Guideline type: scope	Document length	Methodology	Supporting MgSO4 evidence cited
				intact membranes		"using best available evidence and resources…"). Described as 'guidelines'	
SASOG 2023b [23]	SASOG and Better Obs	English	South Africa: Upper-middle income	General: premature ROM	8 pages	Methods not provided in detail ("drafted by a clinical team… reviewed by a panel of experts"; "using best available evidence and resources…"). Described as 'guidelines'	Nil
SLCOG 2022 [24]	SLCOG	English	Sri Lanka: Lower-middle income	General: PROM	6 pages (journal article)	Detailed methods not provided: "guidance in management that have been validated by outcome-based research when available taking Sri Lankan infrastructure facilities into consideration. Where comprehensive and solid evidence is not yet available, recommendations are made on expert opinion and conclusions are made based on limited and inconsistent scientific evidence… Some of the guidance is based on consensus and expert opinion." Described as a 'guideline'	In-text/recommendation citations<br>Notes: "Cochrane review and another two meta-analysis", and describes "the recommendation of RCOG" recommendation – however incorrect citations provided in reference list

Guideline ID	Author/ Developer	Language	Country: World Bank Classificatio n	Guideline type: scope	Document length	Methodology	Supporting MgSO4 evidence cited
SOGOG 2022 [25]	SOGOG	English	Ghana: lower-middle income	General: caesarean section	36 pages	Detailed methods not provided; a Committee of Experts who developed the guideline are named and refers to the evidence that was reviewed. Described as a 'guideline'	General statement regarding evidence "Statements from professional organisations including that of the National Institutes of Health, the American College of Obstetricians and Gynaecologists, the Society for Maternal Foetal Medicine, the Royal College of Obstetrician and Gynaecologists, and the Canadian Paediatric Society were reviewed for additional references."

Abbreviations: ACOG: American College of Obstetricians and Gynecologist; ANCP: Australian National Clinical Practice; CNGOF: French College of Obstetricians and Gynaecologist; FASGO: Federación Argentina de Sociedades de Ginecología y Obstetricia (Argentina Federation of Obstetrics and Gynecology Societies); FECASOG: Federación Centroamericana de Asociaciones y Sociedades de Obstetricia y Ginecología (Central American Federation of Associations and Societies of Obstetrics and Gynecology); FIGO: International Federation of Gynecology and Obstetrics; FOGSI: Federation of Obstetric and Gynaecological Societies of India; Gov: Government; HKFM: Himpunan Kedokteran Feto Maternal (Association of Feto Maternal Medicine); IHCAI: International Health Central American Institute; INPer: Instituto Nacional de Perinatologia (National Institute of Perinataology); IOG: Institute of Obstetricians and Gynaecologists; ISUOG: International Society of Ultrasound in Obstetrics and Gynecology; JPPOBG: Jawatankuasa Pengurusan dan Perkembangan O&G KKM; KCE: Belgian Healthcare Knowledge Centre; MoH: Ministry of Health; NICE: National Institute for Health and Care Excellence; OGSB: Obstetrical and Gynaecological Society of Bangladesh; OGS MMA: Obsetrical and Gynaecological Society, Myanmar Medical Association; PMF: Perinatal Medicine Foundation; PNG O&G Society: Papua New Guinea Obstetrics and Gynecology Society; POGI: Perkumpulan Obstetri dan Ginekologi Indonesia (Indonesian Society of Obstetricians and Gynecologists); PROM: pre-labour rupture of membranes; PSNbM: Philippine Society of Newborn Medicine; PUDER: Perinatoloji Uzmanları Derneği, Turkey (Society of Specialists in Perinatology); QLD: Queensland; RCOG: Royal College of Obstetricians and Gynaecologists; RCTs: randomised controlled trials; ROM: rupture of membranes; SA: South Australia; SASOG: South African Society of Obstetricians and Gynaecologists; SLCOG: Sri Lanka College of Obstetricians and Gynaecologists; SOGC: Society of Obstetricians and Gynaecologists of Canada; SOGOG: Society of Gynaecologists and Obstetricians of Ghana; SRs: systematic reviews; UNPF: United Nations Population Fund; UoA: University of Adelaide; WAPM: World Association of Perinatal Medicine; WHO: World Health Organization

References – Included Guidelines

1. Shennan A, Suff N, Jacobsson B, Simpson JL, Norman J, Grobman WA, et al. FIGO good practice recommendations on magnesium sulfate administration for preterm fetal neuroprotection. International Journal of Gynecology & Obstetrics. 2021;155(1):31-3.
2. Ubom AE, Vatish M, Barnea ER, Childbirth F, Postpartum Hemorrhage C. FIGO good practice recommendations for preterm labor and preterm prelabor rupture of membranes: Prep-for-Labor triage to minimize risks and maximize favorable outcomes. Int J Gynaecol Obstet. 2023;163 Suppl 2:40-50.
3. Lees CC, Stampalija T, Baschat A, da Silva Costa F, Ferrazzi E, Figueras F, et al. ISUOG Practice Guidelines: diagnosis and management of small-for-gestational-age fetus and fetal growth restriction. Ultrasound Obstet Gynecol. 2020;56(2):298-312.
4. Dagklis T, Akolekar R, Villalain C, Tsakiridis I, Kesrouani A, Tekay A, et al. Management of preterm labor: Clinical practice guideline and recommendation by the WAPM-World Association of Perinatal Medicine and the PMF-Perinatal Medicine Foundation. Eur J Obstet Gynecol Reprod Biol. 2023;291:196-205.
5. World Health Organization. WHO recommendations on maternal health: guidelines approved by the WHO Guidelines Review Committee [Licence: CC BY-NC-SA 3.0 IGO.]. 2017 [cited 7 December 2023]. Available from: https://www.who.int/publications/i/item/WHO-MCA-17.10.
6. Federación Argentina de Sociedades de Ginecología y Obstetricia (FASGO). [Rotura prematura de membranas: Actualizado: Consenso FASGO XXXVI] Premature Rupture of Membranes: Updated FASGO Consensus XXXVI 2022 [cited 3 June 2025]. Available from: http://fasgo.org.ar/images/Actualizacion_Consenso_de_RPM.pdf.
7. Federación Centroamericana de Asociaciones y Sociedades de Obstetricia y Ginecología (FECASOG). [Guia de practica clinica para el manejo de parto pretermino] Clinical Practice Guide for the Management of Preterm Labor 2018 [cited 7 December 2023]. Available from: https://aogcr.com/wp-content/uploads/2020/05/GPC_PARTO-PRETE%CC%81RMINO_FECASOG_IHCAI.pdf.
8. Federation of Obstetric and Gynaecological Societies of India (FOGSI). FOGSI Focus: Prevention of pre-term labour 2017 [cited 8 December 2023]. Available from: https://www.fogsi.org/wp-content/uploads/fogsi-focus/fogsi-focus-ptl.pdf.
9. Government of Nepal Ministry of Health and Population Department of Health Services Family Welfare Division. National medical standard for maternal and newborn care, Volume III: Maternal and Newborn Care 2022 [cited 14 April 2025]. Available from: https://fwd.gov.np/wp-content/uploads/2022/10/NMS-Vol-III.pdf.
10. Government of Pakistan: Ministry of National Health Services RC. Clinical protocols and guidelines for emergency obstetric and newborn care 2023 [cited 8 May 2025]. Available from: https://www.nhsrc.gov.pk/Publications.
11. National Institute of Perinatology Mexico (INPer). [Normas y procedimientos de obstetricia 2021] Regulations and obstetric procedures 2021 [cited 06 May 2025]. Available from: https://www.gob.mx/salud/inper/articulos/manual-de-normas-y-procedimientos-de-obstetricia-2021?idiom=es.
12. Ministry of Health (Kenya). National guidelines on quality obstetrics and perinatal care 2024 [cited 14 April 2025]. Available from: TBA.
13. Obstetrical & Gynaecological and Paediatric Services Unit of the Medical Services Development Section, Medical Development Division Ministry of Health Malaysia, Jawatankuasa Pengurusan dan Perkembangan O&G KKM (JPPOBG). Guideline on prevention and management of preterm birth 2023 [cited 6 May 2025]. Available from: https://www.moh.gov.my/moh/resources/Penerbitan/Perkhidmatan%20OnG%20&%20Ped/O%20&%20G/Guideline_on_Prevention_and_Management_(30Aug23).pdf.
14. Ministerio de Salud (Ministry of Health) (Nicaragua). [Normativa 109: Protocolo para la atencion de complicaciones obstetricas] Protocol for the management of obstetric complications 2022 [cited 14 April 2025]. Available from: https://www.scribd.com/document/645344222/Norma-109-17-10-2022-Revisada-y-aprobada-085854-pdf.
15. Ministry of Health of Palestine Women's Health and Development Unit. Obstetric Guidelines and Labor Ward Protocols; Updated version March, 2024 2024 [cited 8 May 2025]. Available from: https://palestine.unfpa.org/en/publications/obstetric-guidelines-and-labor-ward-protocols%C2%A0.
16. Obstetrical and Gynaecological Society of Bangladesh (OGSB). Standard clinical management protocols and flowcharts on emergency obstetric and neonatal care 2019 [cited 14 April 2025]. Available from: https://file-chittagong.portal.gov.bd/uploads/6b9a9df8-2650-4326-bbfd-55418d77e00d//66d/6ef/0b1/66d6ef0b196fa815778442.pdf.

17. Obstetrical & Gynaecological Society Myanmar Medical Association. Obstetrics and Gyanecology Management Guidelines, Second Edition 2024 [cited 14 April 2025]. Available from: https://www.mmacentral.org/societies/obstetrical-and-gynaecological-society/.
18. Papua New Guinea Society of Obstetrics and Gynaecology, Mola GDL. Manual of Standard Managements in Obstetrics and Gynaecology for Doctors, HEOs and Nurses in Papua New Guinea. 7th Edition 2016, with minor amendments 2018 [cited 27 November 2023]. Available from: https://pngpaediatricsociety.org/wp-content/uploads/2022/04/PNG-Standard-Treatment-Manual-for-Obstetrics-and-Gynaecology-7th-Edition-2018.pdf
19. Pengurus Pusat Perkumpulan Obstetri dan Ginekologi Indonesia (Indonesian Obstetrics and Gynecology Association) (POGI), Indonesian Maternal Feto Medicine Association, Indonesian Health Service. [Panduan Persalinan Preterm] Guide to Preterm Childbirth 2019 [cited 27 November 2023]. Available from: https://www.pogi.or.id/document/17/detail.
20. Wilkinson DJ, Villanueva-Uy ME, Hayden D, McTavish J, PSNbM Consensus Working Group. Decision-making around resuscitation of extremely preterm infants in the Philippines: A consensus guideline. J Paediatr Child Health. 2019;55(9):1023-8.
21. Altay M, Bayram M, Biri A, Esim Büyükbayrak E, Deren Ö, Ercan F, et al. Guideline on preterm labor and delivery by the Society of Specialists in Perinatology (Perinatoloji Uzmanlari Dernegi-PUDER), Turkey. J Clin Obstet Gynecol. 2020;30(3):118-30.
22. BetterObs™ South African Society of Obstetricians & Gynaecologists (SASOG). Premature (prelabour) rupture of membranes 2.0 2023 [cited 27 November 2023]. Available from: https://sasog.co.za/betterobs/.
23. BetterObs™ South African Society of Obstetricians & Gynaecologists (SASOG). Preterm labour with intact membranes 2.0 2023 [cited 27 November 2023]. Available from: https://sasog.co.za/betterobs/.
24. De Silva P, Lanerolle S, Dodampahala S, Silva R, Mathota C, on behalf of Sri Lanka College of Obstetricians and Gynaecologists (SLCOG). Prelabour rupture of membranes. Sri Lanka J Obstet Gynaecol. 2022;44(1):75-9.
25. Society of Gynaecologists & Obstetricians of Ghana (SOGOG). National Caesarean Section Guidelines 2022 [cited 8 December 2023]. Available from: https://www.sogog.com/guidelines/.

References (Supporting Evidence Cited)

Guidelines

ACOG 2010:
American College of Obstetricians and Gynecologists. Committee Opinion No. 455: Magnesium sulfate before anticipated preterm birth for neuroprotection. Obstet Gynecol. 2010;115(3):669-71.

ACOG 2013:
American College of Obstetricians and Gynecologists. Committee Opinion No. 573: Magnesium sulfate use in obstetrics. Obstet Gynecol. 2013;122(3):727-8.

ACOG 2016:
American College of Obstetricians and Gynecologists. Practice Bulletin No. 171: Management of Preterm Labor. Obstet Gynecol. 2016;128(4):e155-64.

ACOG 2016b:
American College of Obstetricians and Gynecologists. Practice Bulletin No. 172: Premature Rupture of Membranes. Obstet Gynecol. 2016;128(4):e165-77.

ACOG 2018:
American College of Obstetricians and Gynecologists. Practice Bulletin No. 188: Prelabor Rupture of Membranes. Obstet Gynecol. 2018;131(1):e1-e14.

ACOG 2019:
American College of Obstetricians and Gynecologists. Practice Bulletin No. 204: Fetal Growth Restriction. Obstet Gynecol. 2019;133(2):e97-e109.

Di Renzo 2017:

Di Renzo GC, Cabero Roura L, Facchinetti F, Helmer H, Hubinont C, Jacobsson B, et al. Preterm Labor and Birth Management: Recommendations from the European Association of Perinatal Medicine. J Matern Fetal Neonatal Med. 2017;30(17):2011-30.

FIGO 2021:
Shennan A, Suff N, Jacobsson B, Simpson JL, Norman J, Grobman WA, et al. FIGO good practice recommendations on magnesium sulfate administration for preterm fetal neuroprotection. Int J Gynecol Obstet. 2021;155(1):31-3.

INPer 2021:
National Institute of Perinatology Mexico (INPer). [Normas y procedimientos de obstetricia 2021] Regulations and obstetric procedures 2021 [cited 06 May 2025]. Available from: https://www.gob.mx/salud/inper/articulos/manual-de-normas-y-procedimientos-de-obstetricia-2021?idiom=es.

IOG 2017:
Institute of Obstetricians and Gynaecologists (IOG) - Royal College of Physicians of Ireland And Directorate of Strategy and Clinical Care Health Service Executive. Guideline No 28. Fetal growth restriction- recognition, diagnosis & management. 2017.

NICE 2015:
National Institute for Health and Care Excellence (NICE). Preterm labour and birth. NICE guideline NG25. 2015.

QLD 2016:
Queensland Health. Queensland maternal and neonatal clinical guidelines. Preterm labour. Queensland, Australia; 2016.

RCOG 2019:
Thomson AJ, Royal College of Obstetricians and Gynaecologists. Care of Women Presenting with Suspected Preterm Prelabour Rupture of Membranes from 24(+0) Weeks of Gestation: Green-top Guideline No. 73. BJOG. 2019;126(9):e152-e66.

SA 2015:
South Australia Health Government of South Australia. Preterm labour clinical guidelines: preterm labour. 2015.

SOGC 2011:
Magee L, Sawchuck D, Synnes A, von Dadelszen P, Magnesium Sulphate For Fetal Neuroprotection Consensus C, Maternal Fetal Medicine C. SOGC Clinical Practice Guideline. Magnesium sulphate for fetal neuroprotection. J Obstet Gynaecol Can. 2011;33(5):516-29.

SOGC 2019:
Magee LA, De Silva DA, Sawchuck D, Synnes A, von Dadelszen P. No. 376-Magnesium Sulphate for Fetal Neuroprotection. J Obstet Gynaecol Can. 2019;41(4):505-22.

Sweet 2016:
Sweet DG, Carnielli V, Greisen G, Hallman M, Ozek E, Plavka R, et al. European Consensus Guidelines on the Management of Respiratory Distress Syndrome - 2016 Update. Neonatol. 2017;111(2):107-25.

UoA 2010:
Antenatal Magnesium Sulphate for Neuroprotection Guideline Development Panel. Antenatal Magnesium Sulphate Prior to Preterm Birth for Neuroprotection of the Fetus, Infant, and Child 2010: National Clinical Practice Guidelines. Adelaide: Australian Research Centre for Health of Women and Babies, the University of Adelaide; 2010.

Vayssiere 2015:
Vayssière C, Sentilhes L, Ego A, Bernard C, Cambourieu D, Flamant C, et al. Fetal growth restriction and intra-uterine growth restriction: guidelines for clinical practice from the French College of Gynaecologists and Obstetricians. Eur J Obstet Gynecol Reprod Biol. 2015;193:10-8.

WHO 2015:
World Health Organisation. WHO recommendations on interventions to improve preterm birth outcomes 2015 [cited 30 March 2023]. Available from: https://www.who.int/publications/i/item/9789241508988.

WHO 2016:
World Health Organization. Standards for improving maternal and newborn quality of care in health facilities. Geneva, Swizerland: World Health Organization; 2016.

WHO 2017:
World Health Organization. Guidelines for management of common maternal conditions; Pocket book of hospital care for mothers. New Delhi: World Health Organization, Regional Office for South-East Asia; 2017.

WHO 2017b:
World Health Organization. Managing complications in pregnancy and childbirth: a guide for midwives and doctors – 2nd ed. Geneva: World Health Organization; 2017.

Systematic reviews

Conde-Augdelo 2009:
Conde-Agudelo A, Romero R. Antenatal magnesium sulfate for the prevention of cerebral palsy in preterm infants less than 34 weeks' gestation: a systematic review and metaanalysis. Am J Obstet Gynecol. 2009;200(6):595-609.

Conde-Augdelo 2009:
Costantine MM, Weiner SJ. Effects of antenatal exposure to magnesium sulfate on neuroprotection and mortality in preterm infants: a meta-analysis. Obstet Gynecol. 2009;114(2 Pt 1):354-64.

Doyle 2009:
Doyle LW, Crowther CA, Middleton P, Marret S, Rouse D. Magnesium sulphate for women at risk of preterm birth for neuroprotection of the fetus. Cochrane Database Syst Rev. 2009(1):CD004661.

Galinksy 2020:
Galinsky R, Dean JM, Lingam I, Robertson NJ, Mallard C, Bennet L, et al. A Systematic Review of Magnesium Sulfate for Perinatal Neuroprotection: What Have We Learnt From the Past Decade? Front Neurol. 2020;11:449.

Shepherd 2017:
Shepherd E, Salam RA, Middleton P, Makrides M, McIntyre S, Badawi N, et al. Antenatal and intrapartum interventions for preventing cerebral palsy: an overview of Cochrane systematic reviews. Cochrane Database Syst Rev. 2017;8(8):CD012077.

Shepherd 2019:
Shepherd E, Salam RA, Manhas D, Synnes A, Middleton P, Makrides M, et al. Antenatal magnesium sulphate and adverse neonatal outcomes: A systematic review and meta-analysis. PLoS Med. 2019;16(12):e1002988.

Tsakiridis 2020:
Tsakiridis I, Mamopoulos A, Athanasiadis A, Dagklis T. Antenatal Corticosteroids and Magnesium Sulfate for Improved Preterm Neonatal Outcomes: A Review of Guidelines. Obstet Gynecol Surv. 2020;75(5):298-307.

Wolf 2020:
Wolf HT, Huusom LD, Henriksen TB, Hegaard HK, Brok J, Pinborg A. Magnesium sulphate for fetal neuroprotection at imminent risk for preterm delivery: a systematic review with meta-analysis and trial sequential analysis. BJOG. 2020;127(10):1180-8.

RCTs

Altman 2002:
Altman D, Carroli G, Duley L, Farrell B, Moodley J, Neilson J, et al. Do women with pre-eclampsia, and their babies, benefit from magnesium sulphate? The Magpie Trial: a randomised placebo-controlled trial. Lancet. 2002;359(9321):1877-90.

Crowther 2003:

Crowther CA, Hiller JE, Doyle LW, Haslam RR. Effect of magnesium sulfate given for neuroprotection before preterm birth: a randomized controlled trial. JAMA. 2003;290(20):2669-76.

Crowther 2023:
Crowther CA, Ashwood P, Middleton PF, McPhee A, Tran T, Harding JE, et al. Prenatal Intravenous Magnesium at 30-34 Weeks' Gestation and Neurodevelopmental Outcomes in Offspring: The MAGENTA Randomized Clinical Trial. JAMA. 2023;330(7):603-14.

Marret 2007:
Marret S, Marpeau L, Zupan-Simunek V, Eurin D, Leveque C, Hellot MF, et al. Magnesium sulphate given before very-preterm birth to protect infant brain: the randomised controlled PREMAG trial*. BJOG. 2007;114(3):310-8.

Rouse 2008:
Rouse DJ, Hirtz DG, Thom E, Varner MW, Spong CY, Mercer BM, et al. A randomized, controlled trial of magnesium sulfate for the prevention of cerebral palsy. N Engl J Med. 2008;359(9):895-905.

<u>Other</u>

Chang 2015:
Chang E. Preterm birth and the role of neuroprotection. BMJ. 2015;350:g6661.

Chollat 2018:
Chollat C, Sentilhes L, Marret S. Fetal Neuroprotection by Magnesium Sulfate: From Translational Research to Clinical Application. Front Neurol. 2018;9:247.

Stockley 2018:
Stockley EL, Ting JY, Kingdom JC, McDonald SD, Barrett JF, Synnes AR, et al. Intrapartum magnesium sulfate is associated with neuroprotection in growth-restricted fetuses. Am J Obstet Gynecol. 2018;219(6):606.e1-.e8.

Ting 2018:
Ting JY, Kingdom JC, Shah PS. Antenatal glucocorticoids, magnesium sulfate, and mode of birth in preterm fetal small for gestational age. Am J Obstet Gynecol. 2018;218(2s):S818-s28.

S6 Table. Characteristics of guidelines (2)

Guideline ID (Country)	MgSO4 recommendation characteristics							
	**Primary reason for preterm birth risk**	**GA**	**No. babies in utero**	**Mode**	**LD**	**MD**	**Repeat treatment**	**Time before birth**
**International**								
FIGO 2021 [1]	Women at risk of early preterm imminent birth, regardless of the cause for preterm birth	Recommended: from viability to 30 weeks' GA<br><br>Considered: < 32 to 34 weeks' GA	Regardless of the number of babies in utero	IV	4 g over 20-30 min	1 g/hour until birth, but stop after 24 hours if undelivered	Not specified	When preterm birth is planned or expected within 24 hours; when birth is planned, administer as close as possible to 4 hours before birth; if birth expected sooner than 4 hours, still administer
FIGO 2023 [2]	Women with established preterm labour or PPROM at risk of imminent preterm birth within 24 hours	Viability (> 23 weeks' GA) to 33+6 weeks' GA	Singleton or multiple pregnancy	*LMIC setting simplified regimen*			No repeat MgSO4 after an initial course	Not specified, though recommended when there is risk of imminent birth within 24 hours
				IV & IM	4 g IV over 20-30 min and 10 g IM	5 g IM every 4 hours for 24 hours		
				High-resource setting regimen				
				IV	4 g over 20-30 min	1 g/hour until delivery or for up to 24 hours		
ISUOG 2020 [3]	Not specified; however, guideline relates specifically to small-for-gestational age and fetal growth restriction	Refer to local or national guidelines (variation notes: < 32-33, < 32, < 30, < 29 weeks' GA)	Not specified	Not specified	Not specified	Not specified	Not specified	Not specified

Guideline ID (Country)	MgSO4 recommendation characteristics							
	**Primary reason for preterm birth risk**	**GA**	**No. babies in utero**	**Mode**	**LD**	**MD**	**Repeat treatment**	**Time before birth**
WAPM & PMF 2023 [4]	Guideline relates to preterm labour; specifically mentions small-for gestational fetuses	< 32 weeks' GA (and < 34 weeks' GA in small-for-gestational age fetuses < 5th centile)	Not specified	IV	4 g over 15-30 min	1 g/hour until delivery or for up to 24 hours	Not specified	Not specified (mentions "Magnesium sulfate offers fetal neuroprotection when administered for at least 4 h…")
WHO 2017 [5]	Women at risk of imminent preterm birth	< 32 weeks' GA	Not specified	Not specified	Not specified	Not specified	Not specified	Not specified
**National/multi-national**								
FASGO 2022 (Argentina) [6]	Guideline relates to premature ROM: specifies recommendation for pregnancies with imminent premature delivery, spontaneous or indicated by maternal and/or fetal causes	24 to 32.0 weeks' GA (also: consider indication for 23.0 to 23.6 weeks' GA)	Not specified	IV	5 g over 30 min	1 g/hour until delivery no longer imminent or for up to 24 hours	No (do not repeat regimen if premature delivery does not occur and is restared later)	Not specified, though recommended for pregnancies with imminent premature delivery; (mentions "for a period of 4 or more hours")
FECASOG 2018 (Central America) [7]	Not specified	24 to 32 weeks' GA	Not specified	Not specified	Not specified	Not specified	Not specified	Not specified
FOGSI 2017 (India) [8]	Intranatal management of preterm labour: women in established preterm	24 to 31 + 6 weeks' GA	Intranatal management of preterm labour: no specified	IV	4 g over 15 minutes	1 g/hour until birth or for 24 hours (whichever is sooner)	Not specified	Not specified

Guideline ID (Country)	MgSO4 recommendation characteristics							
	**Primary reason for preterm birth risk**	**GA**	**No. babies in utero**	**Mode**	**LD**	**MD**	**Repeat treatment**	**Time before birth**
	labour or having a planned preterm birth within 24 hours; excluding: active labour at greater than 8 cm dilatation, major fetal abnormalities, maternal contraindications to MgSO4 (pulmonary hypertension, myasthenia gravis, class II-IV cardiac disease, severe acute pulmonary disease, pulmonary insufficiency)		Recommend ation provided separately for twin also					
Gov Nepal MoH 2022 [9]	In a section on 'Standard for management of RDS':<br>Women with imminent preterm birth (active labour with 4 cm of cervical dilation, with or without PPROM and planned preterm deliveries for fetal or maternal indications)	Less than 31 weeks' GA	Not specified	Not specified	Not specified	Not specified	Not specified	Not specified, though recommended for women with imminent preterm birth
Gov Pakistan 2023 [10]	Women with imminent preterm labour	Not specified	Not specified	IV	4 g over 30 min	With or without 1	Not specified	Not specified, though recommended for

Guideline ID (Country)	MgSO4 recommendation characteristics							
	Primary reason for preterm birth risk	GA	No. babies in utero	Mode	LD	MD	Repeat treatment	Time before birth
						g/hour until birth		women with imminent preterm labour
INPer 2021 (Mexico) [11]	Patients with pregnancies who will potentially birth in the next 24 hours for any of the following reasons: preterm labour, IUGR, PROM, hypertensive disorders of pregnancy. Contraindications: severe kidney and/or liver damage, myocardial damage or cardiac conduction disturbance, myasthenia gravis, shock of any nature; fetal malformations incompatible with life	26+0 to 31+6 weeks' GA	Single or multiple	IV	4 g over 20-30 min	1 g/hour for at least 12 hours, or until birth (not discontinued if used for hypertensive disorders of pregnancy); if birth does not occur within 12 hours, consider discontinuing it if birth is not imminent	If preterm birth is again imminent, treatment should be restarted; if > 6 hours passed since completion of previous dose, LD and MD should be repeated; if < 6 hours have passed, re-start MD only	Not specified, though recommended with potential delivery in next 24 hours (and re-treatment if birth is again imminent)
MoH Kenya 2024 [12]	PPROM section: before preterm birth<br><br>Preterm labour section: women at risk of imminent preterm birth	Preterm labour section: < 32 weeks' GA in Table (< 30 weeks' GA in	Not specified	IV	PPROM section: 4 g<br><br>Preterm labour section: 4 g over 20 min	PPROM section: 1 g/hour for a maximum of 24 hours<br><br>Preterm labour	Not specified	Not specified

**Guideline ID (Country)**	**MgSO4 recommendation characteristics**							
	**Primary reason for preterm birth risk**	**GA**	**No. babies in utero**	**Mode**	**LD**	**MD**	**Repeat treatment**	**Time before birth**
		algorithm; 24-30 weeks' GA in another algorithm)				section: 1 g/hour for 4 hours in one algorithm; 1 g/hour for 24 hours or until birth in another algorithm		
MoH Malaysia 2023 [13]	Women in established preterm labour where delivery is likely to occur soon	24 to 33+6 weeks' GA	Not specified	*Specialist hospitals*			Not specified	Not specified, though recommended where delivery is likely to occur soon
				IV	4 g over 15 minutes	1 g/hour until birth or for 24 hours whichever is sooner		
				District hospitals and peripheral health centres				
				IM	10 g bolus	5 g every 4 hours until birth or for 24 hours whichever is sooner		
MoH Nicaragua 2022 [14]	Mentioned in sections: Placenta praevia; PROM; newborn RDS – women in imminent labour	Placenta praevia recommendation						
		Between 28- and 32-weeks' GA	Not specified	'Zuspan regimen' [IV]	'Zuspan regimen' 4 g over 5-15 min	'Zuspan regimen' [1 g/hour]	Not specified	6-8 hours before the end of pregnancy
		PROM and newborn RDS recommendations						

**Guideline ID (Country)**	**MgSO4 recommendation characteristics**							
	**Primary reason for preterm birth risk**	**GA**	**No. babies in utero**	**Mode**	**LD**	**MD**	**Repeat treatment**	**Time before birth**
		< 32 weeks' GA	Not specified	Not specified	Not specified	Not specified	Not specified	Not specified
MoH Palestine 2024 (West Bank and Gaza) [15]	Women in established preterm labour or having a planned preterm birth within 24 hours	24 to 32 weeks' GA	Not specified	IV (assumed)	4 g over 20 min	1 g/hour until birth or for 24 hours	No immediate repeat doses	Not specified, though recommended for women in established labour or planned preterm birth within 24 hours
					6 g over 20 min	2 g/hour until birth or for 12 hours	If < 6 hours have elapsed since discontinuation of infusion, restart MD; if > 6 hours have elapsed, re-bolus and start MD	
OGSB 2019 (Bangladesh) [16]	In a section on preterm labour: women at risk of imminent preterm birth	< 32 weeks' GA	Not specified	IV	4 g over 20 min	1 g/hour until delivery or for 24 hours whichever came first	Not specified	Not specified, though recommended for women at risk of imminent preterm birth
					4 g over 30 min, or 4 g bolus	Nil		
					6 g over 20-30 min	2 g/hour (duration not specified)		
OGS MMA 2024 (Myanmar) [17]	Recommendations provided in sections on chronic hypertension, gestational hypertension, pre-eclampsia, COVID-19 – if early birth is planned	Pre-eclampsia: < 32 weeks' GA	Not specified	Not specified	Not specified	Not specified	Not specified	Not specified
		< 32 weeks		"same dose as for PET"				

Guideline ID (Country)	MgSO4 recommendation characteristics							
	**Primary reason for preterm birth risk**	**GA**	**No. babies in utero**	**Mode**	**LD**	**MD**	**Repeat treatment**	**Time before birth**
PNG O&G Society 2018 [18]	Not specified; however in a section on preterm labour		Not specified	IV and IM	14 g (4 g IV and 10 g IM)	5 g IM 6 hours post loading dose and every 6 hours until 24 hours	Not specified	Not specified
POGI 2019 (Indonesia) [19]	Patients who are expected to experience preterm birth within 24 hours	23 to 32 weeks' GA<br><br>*Note: inconsistency in lower GA, as also reported as 24	Not specified	IV	4 g over 30 minutes	1 g/hour for a maximum of 24 hours or labour occurs	Not specified	Not specified, though recommended for women expected to birth within 24 hours
PSNbM 2019 (Philippines) [20]	Where delivery of a *potentially viable* extremely preterm infant is anticipated; and where preterm labour is apparent, delivery is imminent and where there is a plan to provide resuscitation of the preterm infant	Not specified (guideline for extremely preterm infants)	Not specified	Not specified	Not specified	Not specified	Not specified	Not specified, though recommended when delivery is imminent
PUDER 2020 (Turkey) [21]	Not specified; however, guideline relates specifically to preterm labour (if delivery is expected within 24 hours) and PPROM	24+0 to 31+6 weeks' GA	Not specified	IV	4-6 g over 15-20 min	1-2 g/hour, stopped if delivery does not occur after 48 hours of treatment	Not specified	Not specified, though recommended when delivery is expected within 24 hours

<table>
<tr><th rowspan="2">Guideline ID (Country)</th><th colspan="8">MgSO4 recommendation characteristics</th></tr>
<tr><th>Primary reason for preterm birth risk</th><th>GA</th><th>No. babies in utero</th><th>Mode</th><th>LD</th><th>MD</th><th>Repeat treatment</th><th>Time before birth</th></tr>
<tr><td>SASOG 2023a (South Africa) [22]</td><td>Guideline is specific to preterm labour; confirmed preterm labour (contractions and cervical change but intact membranes), if delivery is imminent in next 24 hours</td><td>26-32 weeks’ GA</td><td>Not specified</td><td>Not specified</td><td>Not specified</td><td>Not specified</td><td>Not specified</td><td>Not specified, though recommended if delivery imminent in next 24 hours</td></tr>
<tr><td>SASOG 2023b (South Africa) [23]</td><td>Guideline is specific to PPROM</td><td>After viability, < 32 weeks</td><td>Not specified</td><td>Not specified</td><td>Not specified</td><td>Not specified</td><td>Not specified</td><td>Not specified, though recommended if delivery imminent in next 24 hours</td></tr>
<tr><td rowspan="2">SLCOG 2022 (Sri Lanka) [24]</td><td rowspan="2">Guideline is specific to PROM</td><td rowspan="2">Not clearly recommended; cites “benefit was greatest before 30 weeks of gestation” (Doyle 2009), and cites RCOG up to 33 + 6 weeks’ GA</td><td rowspan="2">Not specified</td><td colspan="5">“in recommended doses for neuroprotection as in the guidance on hypertensive disorders”</td></tr>
<tr><td>Not specified</td><td>Not specified</td><td>Not specified</td><td>Not specified</td><td>Not specified</td></tr>
<tr><td>SOGOG 2022 (Ghana) [25]</td><td>Guideline is specific to preterm caesarean section for maternal and/or fetal indications</td><td>”Severe preterm”, 28 to < 32 weeks’ GA</td><td>Not specified</td><td>IV</td><td>4 g</td><td>1 g/hour</td><td>Not specified</td><td>Ideally within 4 hours of intended delivery</td></tr>
</table>

Abbreviations: ACOG: American College of Obstetricians and Gynecologist; FASGO: Federación Argentina de Sociedades de Ginecología y Obstetricia (Argentina Federation of Obstetrics and Gynecology Societies); FECASOG: Federación Centroamericana de Asociaciones y Sociedades de Obstetricia y Ginecología (Central American Federation of Associations and Societies of Obstetrics and Gynecology); FIGO: International Federation of Gynecology and Obstetrics; FOGSI: Federation of Obstetric and Gynaecological Societies of India; g: grams; GA: gestational age; Gov: Government; IM: intramuscular; INPer: Instituto Nacional de Perinatologia (National Institute of Perinataology); ISUOG: International Society of Ultrasound in Obstetrics and Gynecology; IUGR: intrauterine growth restriction; IV: intravenous; LD: loading dose; MD: maintenance dose; min: minutes; MoH: Ministry of Health; OGSB: Obstetrical and Gynaecological Society of Bangladesh; OGS MMA: Obsetrical and Gynaecological Society, Myanmar Medical Association; PET: pre-eclampsia; PMF: Perinatal Medicine Foundation; PNG O&G Society: Papua New Guinea Obstetrics and Gynecology Society; POGI: *Perkumpulan Obstetri dan Ginekologi Indonesia* (Indonesian Society of Obstetricians and Gynecologists); PPROM: preterm pre-labour rupture of membranes; PROM: pre-labour rupture of membranes; PSNbM: Philippine Society of Newborn Medicine; PUDER: Perinatoloji Uzmanları Derneği, Turkey (Society of Specialists in Perinatology); RCOG: Royal College of Obstetricians and Gynaecologists; RDS: respiratory distress syndrome; ROM: rupture of membranes; SASOG: South African Society of Obstetricians and Gynaecologists; SLCOG: Sri Lanka College of Obstetricians and Gynaecologists; SOGOG: Society of Gynaecologists and Obstetricians of Ghana; WAPM: World Association of Perinatal Medicine; WHO: World Health Organization

References – Included Guidelines

1. Shennan A, Suff N, Jacobsson B, Simpson JL, Norman J, Grobman WA, et al. FIGO good practice recommendations on magnesium sulfate administration for preterm fetal neuroprotection. International Journal of Gynecology & Obstetrics. 2021;155(1):31-3.
2. Ubom AE, Vatish M, Barnea ER, Childbirth F, Postpartum Hemorrhage C. FIGO good practice recommendations for preterm labor and preterm prelabor rupture of membranes: Prep-for-Labor triage to minimize risks and maximize favorable outcomes. Int J Gynaecol Obstet. 2023;163 Suppl 2:40-50.
3. Lees CC, Stampalija T, Baschat A, da Silva Costa F, Ferrazzi E, Figueras F, et al. ISUOG Practice Guidelines: diagnosis and management of small-for-gestational-age fetus and fetal growth restriction. Ultrasound Obstet Gynecol. 2020;56(2):298-312.
4. Dagklis T, Akolekar R, Villalain C, Tsakiridis I, Kesrouani A, Tekay A, et al. Management of preterm labor: Clinical practice guideline and recommendation by the WAPM-World Association of Perinatal Medicine and the PMF-Perinatal Medicine Foundation. Eur J Obstet Gynecol Reprod Biol. 2023;291:196-205.
5. World Health Organization. WHO recommendations on maternal health: guidelines approved by the WHO Guidelines Review Committee [Licence: CC BY-NC-SA 3.0 IGO.]. 2017 [cited 7 December 2023]. Available from: https://www.who.int/publications/i/item/WHO-MCA-17.10.
6. Federación Argentina de Sociedades de Ginecología y Obstetricia (FASGO). [Rotura prematura de membranas: Actualizado: Consenso FASGO XXXVI] Premature Rupture of Membranes: Updated FASGO Consensus XXXVI 2022 [cited 3 June 2025]. Available from: http://fasgo.org.ar/images/Actualizacion_Consenso_de_RPM.pdf.
7. Federación Centroamericana de Asociaciones y Sociedades de Obstetricia y Ginecología (FECASOG). [Guia de practica clinica para el manejo de parto pretermino] Clinical Practice Guide for the Management of Preterm Labor 2018 [cited 7 December 2023]. Available from: https://aogcr.com/wp-content/uploads/2020/05/GPC_PARTO-PRETE%CC%81RMINO_FECASOG_IHCAI.pdf.
8. Federation of Obstetric and Gynaecological Societies of India (FOGSI). FOGSI Focus: Prevention of pre-term labour 2017 [cited 8 December 2023]. Available from: https://www.fogsi.org/wp-content/uploads/fogsi-focus/fogsi-focus-ptl.pdf.
9. Government of Nepal Ministry of Health and Population Department of Health Services Family Welfare Division. National medical standard for maternal and newborn care, Volume III: Maternal and Newborn Care 2022 [cited 14 April 2025]. Available from: https://fwd.gov.np/wp-content/uploads/2022/10/NMS-Vol-III.pdf.
10. Government of Pakistan: Ministry of National Health Services RC. Clinical protocols and guidelines for emergency obstetric and newborn care 2023 [cited 8 May 2025]. Available from: https://www.nhsrc.gov.pk/Publications.
11. National Institute of Perinatology Mexico (INPer). [Normas y procedimientos de obstetricia 2021] Regulations and obstetric procedures 2021 [cited 06 May 2025]. Available from: https://www.gob.mx/salud/inper/articulos/manual-de-normas-y-procedimientos-de-obstetricia-2021?idiom=es.
12. Ministry of Health (Kenya). National guidelines on quality obstetrics and perinatal care 2024 [cited 14 April 2025]. Available from: TBA.
13. Obstetrical & Gynaecological and Paediatric Services Unit of the Medical Services Development Section, Medical Development Division Ministry of Health Malaysia, Jawatankuasa Pengurusan dan Perkembangan O&G KKM (JPPOBG). Guideline on prevention and management of preterm birth 2023 [cited 6 May 2025]. Available from: https://www.moh.gov.my/moh/resources/Penerbitan/Perkhidmatan%20OnG%20&%20Ped/O%20&%20G/Guideline_on_Prevention_and_Management_(30Aug23).pdf.
14. Ministerio de Salud (Ministry of Health) (Nicaragua). [Normativa 109: Protocolo para la atencion de complicaciones obstetricas] Protocol for the management of obstetric complications 2022 [cited 14 April 2025]. Available from: https://www.scribd.com/document/645344222/Norma-109-17-10-2022-Revisada-y-aprobada-085854-pdf.
15. Ministry of Health of Palestine Women's Health and Development Unit. Obstetric Guidelines and Labor Ward Protocols; Updated version March, 2024 2024 [cited 8 May 2025]. Available from: https://palestine.unfpa.org/en/publications/obstetric-guidelines-and-labor-ward-protocols%C2%A0.
16. Obstetrical and Gynaecological Society of Bangladesh (OGSB). Standard clinical management protocols and flowcharts on emergency obstetric and neonatal care 2019 [cited 14 April 2025]. Available from: https://file-chittagong.portal.gov.bd/uploads/6b9a9df8-2650-4326-bbfd-55418d77e00d//66d/6ef/0b1/66d6ef0b196fa815778442.pdf.

17. Obstetrical & Gynaecological Society Myanmar Medical Association. Obstetrics and Gyanecology Management Guidelines, Second Edition 2024 [cited 14 April 2025]. Available from: https://www.mmacentral.org/societies/obstetrical-and-gynaecological-society/.
18. Papua New Guinea Society of Obstetrics and Gynaecology, Mola GDL. Manual of Standard Managements in Obstetrics and Gynaecology for Doctors, HEOs and Nurses in Papua New Guinea. 7th Edition 2016, with minor amendments 2018 [cited 27 November 2023]. Available from: https://pngpaediatricsociety.org/wp-content/uploads/2022/04/PNG-Standard-Treatment-Manual-for-Obstetrics-and-Gynaecology-7th-Edition-2018.pdf
19. Pengurus Pusat Perkumpulan Obstetri dan Ginekologi Indonesia (Indonesian Obstetrics and Gynecology Association) (POGI), Indonesian Maternal Feto Medicine Association, Indonesian Health Service. [Panduan Persalinan Preterm] Guide to Preterm Childbirth 2019 [cited 27 November 2023]. Available from: https://www.pogi.or.id/document/17/detail.
20. Wilkinson DJ, Villanueva-Uy ME, Hayden D, McTavish J, PSNbM Consensus Working Group. Decision-making around resuscitation of extremely preterm infants in the Philippines: A consensus guideline. J Paediatr Child Health. 2019;55(9):1023-8.
21. Altay M, Bayram M, Biri A, Esim Büyükbayrak E, Deren Ö, Ercan F, et al. Guideline on preterm labor and delivery by the Society of Specialists in Perinatology (Perinatoloji Uzmanlari Dernegi-PUDER), Turkey. J Clin Obstet Gynecol. 2020;30(3):118-30.
22. BetterObs™ South African Society of Obstetricians & Gynaecologists (SASOG). Premature (prelabour) rupture of membranes 2.0 2023 [cited 27 November 2023]. Available from: https://sasog.co.za/betterobs/.
23. BetterObs™ South African Society of Obstetricians & Gynaecologists (SASOG). Preterm labour with intact membranes 2.0 2023 [cited 27 November 2023]. Available from: https://sasog.co.za/betterobs/.
24. De Silva P, Lanerolle S, Dodampahala S, Silva R, Mathota C, on behalf of Sri Lanka College of Obstetricians and Gynaecologists (SLCOG). Prelabour rupture of membranes. Sri Lanka J Obstet Gynaecol. 2022;44(1):75-9.
25. Society of Gynaecologists & Obstetricians of Ghana (SOGOG). National Caesarean Section Guidelines 2022 [cited 8 December 2023]. Available from: https://www.sogog.com/guidelines/.
